# Supplementary figures and images for: Dermal formulation based on carbopol and Gum Arabic improves skin retention of indomethacin
Source: PLoS One. 2025 Jun 10;20(6):e0326051. doi: 10.1371/journal.pone.0326051 (PMC12151425; doi:10.1371/journal.pone.0326051)

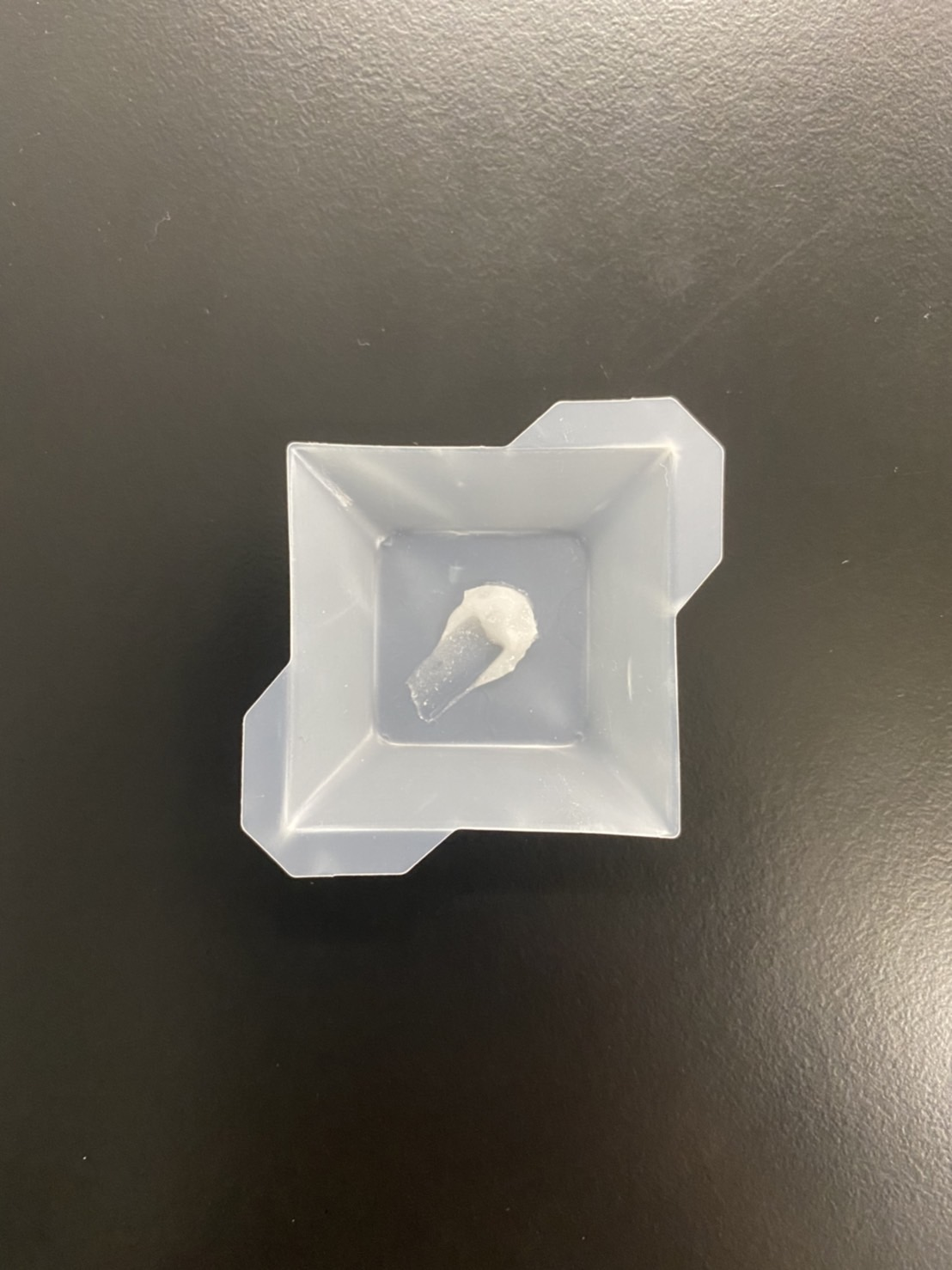

Supplement: S1 Fig — Full image of IMC-MP@GCgel shown in Fig 1D. (TIF) [file pone.0326051.s001.tif]

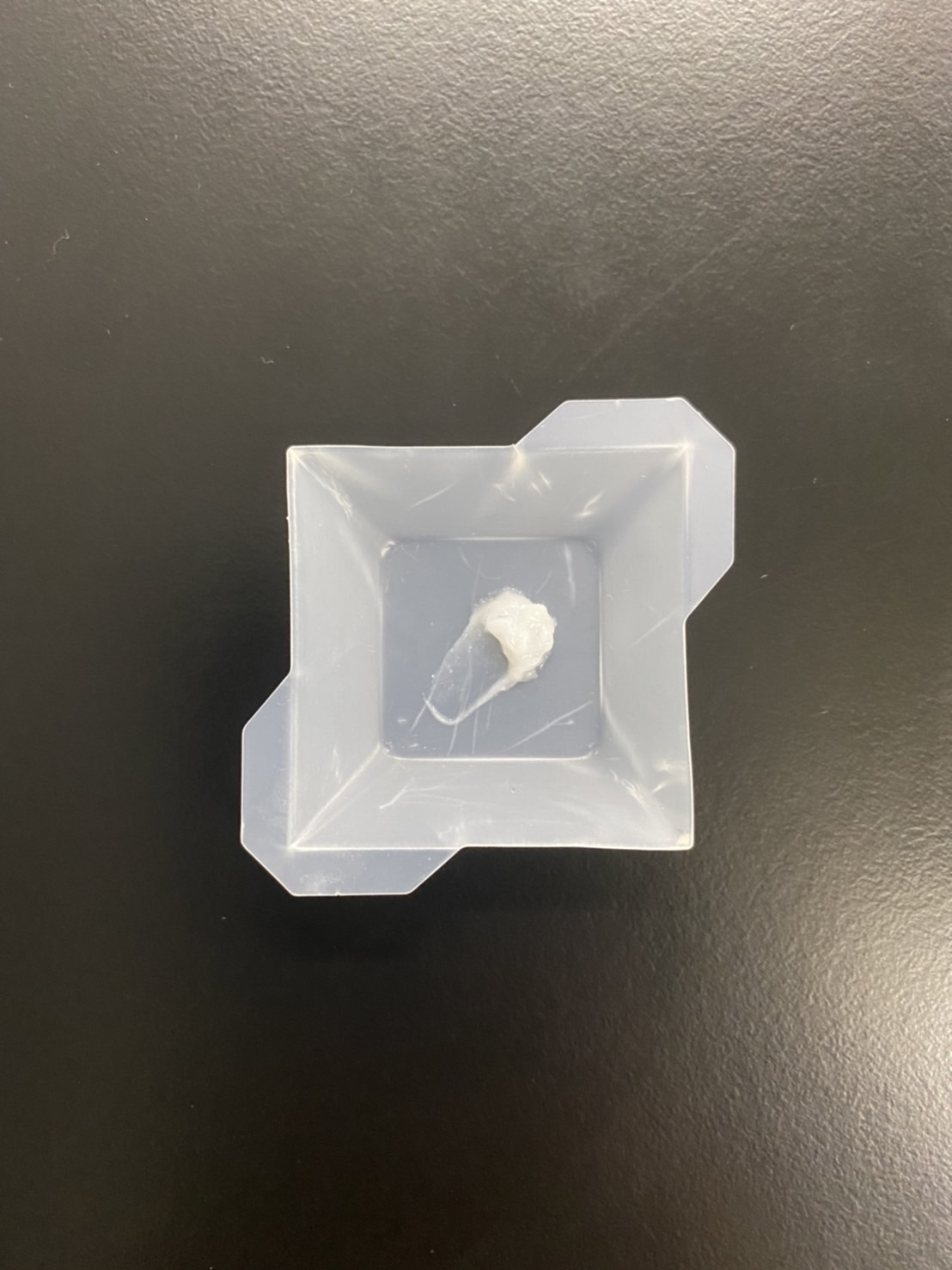

Supplement: S2 Fig — Full image of IMC-NP@GCgel shown in Fig 1D. (TIF) [file pone.0326051.s002.tif]
