# Supplementary material for: Dermal formulation based on carbopol and Gum Arabic improves skin retention of indomethacin
Source: PLoS One. 2025 Jun 10;20(6):e0326051. doi: 10.1371/journal.pone.0326051 (PMC12151425; doi:10.1371/journal.pone.0326051)
Supplement: S1 Table — Raw data in Fig 2A and 2B. (PDF) [file pone.0326051.s003.pdf]

Figure 2A

2θ (degree)	Intensity (arb. unit)
5	9500
5.02	9500
5.04	9916.67
5.06	10300
5.08	10158.3
5.1	10366.7
5.12	10233.3
5.14	10025
5.16	10366.7
5.18	10308.3
5.2	10050
5.22	10233.3
5.24	10525
5.26	10300
5.28	10100
5.3	10125
5.32	10008.3
5.34	10066.7
5.36	10133.3
5.38	10116.7
5.4	10175
5.42	10275
5.44	10358.3
5.46	10458.3
5.48	10558.3
5.5	10316.7
5.52	10216.7
5.54	10416.7
5.56	10483.3
5.58	10366.7
5.6	10200
5.62	10125
5.64	10316.7

Figure 2B

Vehicle

2θ (degree)	Intensity (arb. unit)
5	15658.3
5.02	15658.3
5.04	15950
5.06	16333.3
5.08	16258.3
5.1	16083.3
5.12	16058.3
5.14	16308.3
5.16	16750
5.18	16866.7
5.2	16466.7
5.22	16241.7
5.24	16358.3
5.26	16641.7
5.28	16716.7
5.3	16475
5.32	16841.7
5.34	16966.7
5.36	16950
5.38	16750
5.4	16891.7
5.42	17141.7
5.44	17216.7
5.46	17175
5.48	17175
5.5	17316.7
5.52	17366.7
5.54	17358.3
5.56	17200
5.58	17525
5.6	18033.3
5.62	17800
5.64	17091.7

IMC-MP@GCgel

2θ (degree)	Intensity (arb. unit)
5	9941.67
5.02	9941.67
5.04	10033.3
5.06	10141.7
5.08	10200
5.1	10300
5.12	10291.7
5.14	10408.3
5.16	10525
5.18	10208.3
5.2	10041.7
5.22	10291.7
5.24	10608.3
5.26	10533.3
5.28	10225
5.3	10041.7
5.32	9975
5.34	10025
5.36	10133.3
5.38	10233.3
5.4	10366.7
5.42	10441.7
5.44	10550
5.46	10325
5.48	10341.7
5.5	10133.3
5.52	10141.7
5.54	10158.3
5.56	10133.3
5.58	10283.3
5.6	10275
5.62	10341.7
5.64	10300

IMC-NP@GCgel

2θ (degree)	Intensity (arb. unit)
5	10591.7
5.02	10591.7
5.04	10325
5.06	10100
5.08	10150
5.1	10041.7
5.12	10158.3
5.14	10008.3
5.16	10058.3
5.18	10300
5.2	9950
5.22	9950
5.24	10216.7
5.26	10216.7
5.28	9933.33
5.3	9825
5.32	9825
5.34	10158.3
5.36	9916.67
5.38	9908.33
5.4	9983.33
5.42	10008.3
5.44	9966.67
5.46	9583.33
5.48	9616.67
5.5	9983.33
5.52	9883.33
5.54	9925
5.56	10025
5.58	9791.67
5.6	9800
5.62	10133.3
5.64	9875

5.66	10425
5.68	10316.7
5.7	10483.3
5.72	10458.3
5.74	10533.3
5.76	10591.7
5.78	10450
5.8	10500
5.82	10350
5.84	10241.7
5.86	10150
5.88	10466.7
5.9	10500
5.92	10233.3
5.94	10233.3
5.96	10183.3
5.98	10216.7
6	10575
6.02	10758.3
6.04	10466.7
6.06	10391.7
6.08	10383.3
6.1	10483.3
6.12	10233.3
6.14	10008.3
6.16	10325
6.18	10258.3
6.2	10366.7
6.22	10341.7
6.24	10025
6.26	10091.7
6.28	10175
6.3	10441.7
6.32	10466.7
6.34	10516.7
6.36	10558.3

5.66	17200
5.68	17733.3
5.7	17908.3
5.72	18066.7
5.74	17508.3
5.76	17633.3
5.78	17883.3
5.8	17558.3
5.82	17591.7
5.84	17758.3
5.86	18283.3
5.88	18575
5.9	18066.7
5.92	18208.3
5.94	18691.7
5.96	18183.3
5.98	18233.3
6	18450
6.02	18083.3
6.04	18341.7
6.06	18425
6.08	18233.3
6.1	18600
6.12	18466.7
6.14	18533.3
6.16	18850
6.18	19000
6.2	18900
6.22	18891.7
6.24	19375
6.26	19533.3
6.28	19416.7
6.3	19158.3
6.32	19066.7
6.34	19475
6.36	19783.3

5.66	10141.7
5.68	10166.7
5.7	10458.3
5.72	10483.3
5.74	10300
5.76	10366.7
5.78	10475
5.8	10483.3
5.82	10516.7
5.84	10325
5.86	10158.3
5.88	10383.3
5.9	10458.3
5.92	10616.7
5.94	10641.7
5.96	10566.7
5.98	10408.3
6	10350
6.02	10441.7
6.04	10508.3
6.06	10800
6.08	10566.7
6.1	10616.7
6.12	10666.7
6.14	10541.7
6.16	10525
6.18	10333.3
6.2	10058.3
6.22	10083.3
6.24	10541.7
6.26	10491.7
6.28	10233.3
6.3	10391.7
6.32	10116.7
6.34	10258.3
6.36	10750

5.66	9691.67
5.68	9908.33
5.7	10266.7
5.72	10241.7
5.74	9950
5.76	10058.3
5.78	10366.7
5.8	10100
5.82	10066.7
5.84	10416.7
5.86	10508.3
5.88	10141.7
5.9	9925
5.92	9983.33
5.94	10150
5.96	10283.3
5.98	10141.7
6	10116.7
6.02	10191.7
6.04	10100
6.06	9958.33
6.08	10025
6.1	10208.3
6.12	10233.3
6.14	10391.7
6.16	10116.7
6.18	10150
6.2	10391.7
6.22	10308.3
6.24	10483.3
6.26	10433.3
6.28	10175
6.3	10233.3
6.32	10325
6.34	10058.3
6.36	10300

6.38	10275
6.4	10250
6.42	10316.7
6.44	10416.7
6.46	10075
6.48	10175
6.5	10425
6.52	10458.3
6.54	10500
6.56	10491.7
6.58	10583.3
6.6	10666.7
6.62	10633.3
6.64	10458.3
6.66	10400
6.68	10266.7
6.7	10150
6.72	10166.7
6.74	10416.7
6.76	10583.3
6.78	10625
6.8	10691.7
6.82	10666.7
6.84	10366.7
6.86	10525
6.88	10875
6.9	10991.7
6.92	10741.7
6.94	10508.3
6.96	10925
6.98	10766.7
7	10616.7
7.02	10491.7
7.04	10316.7
7.06	10483.3
7.08	10625

6.38	19616.7
6.4	19316.7
6.42	19400
6.44	19391.7
6.46	19250
6.48	19550
6.5	19875
6.52	19591.7
6.54	19741.7
6.56	19683.3
6.58	19583.3
6.6	19533.3
6.62	19625
6.64	20391.7
6.66	20416.7
6.68	20175
6.7	20475
6.72	20300
6.74	20366.7
6.76	20375
6.78	20508.3
6.8	20575
6.82	20933.3
6.84	21125
6.86	20900
6.88	20708.3
6.9	21150
6.92	21391.7
6.94	21158.3
6.96	21216.7
6.98	21383.3
7	21666.7
7.02	21866.7
7.04	22100
7.06	21816.7
7.08	21425

6.38	10483.3
6.4	10316.7
6.42	10525
6.44	10508.3
6.46	10525
6.48	10658.3
6.5	10608.3
6.52	10908.3
6.54	10966.7
6.56	10741.7
6.58	10758.3
6.6	10425
6.62	10533.3
6.64	10691.7
6.66	10591.7
6.68	10758.3
6.7	10825
6.72	10941.7
6.74	10641.7
6.76	10358.3
6.78	10366.7
6.8	10425
6.82	10691.7
6.84	10808.3
6.86	10358.3
6.88	10316.7
6.9	10825
6.92	10741.7
6.94	10616.7
6.96	10758.3
6.98	10650
7	10650
7.02	10875
7.04	10891.7
7.06	10833.3
7.08	11091.7

6.38	10350
6.4	10025
6.42	10008.3
6.44	10125
6.46	10383.3
6.48	10616.7
6.5	10600
6.52	10450
6.54	10366.7
6.56	10175
6.58	10283.3
6.6	10533.3
6.62	10466.7
6.64	10558.3
6.66	10733.3
6.68	10791.7
6.7	10425
6.72	10158.3
6.74	10250
6.76	10391.7
6.78	10450
6.8	10400
6.82	10550
6.84	10591.7
6.86	10500
6.88	10575
6.9	10433.3
6.92	10550
6.94	10733.3
6.96	10758.3
6.98	10875
7	10541.7
7.02	10508.3
7.04	10825
7.06	10875
7.08	10700

7.1	10500
7.12	10533.3
7.14	10691.7
7.16	10750
7.18	10975
7.2	11075
7.22	11108.3
7.24	10800
7.26	10641.7
7.28	10841.7
7.3	11083.3
7.32	10883.3
7.34	10691.7
7.36	10808.3
7.38	10883.3
7.4	11075
7.42	10708.3
7.44	10741.7
7.46	10991.7
7.48	10766.7
7.5	10741.7
7.52	11050
7.54	11091.7
7.56	10966.7
7.58	10925
7.6	10875
7.62	11200
7.64	11083.3
7.66	10975
7.68	11091.7
7.7	11166.7
7.72	11341.7
7.74	11375
7.76	11200
7.78	11241.7
7.8	11075

7.1	21791.7
7.12	21891.7
7.14	21683.3
7.16	21775
7.18	22225
7.2	22125
7.22	21716.7
7.24	22008.3
7.26	22066.7
7.28	22141.7
7.3	22758.3
7.32	22875
7.34	22583.3
7.36	22525
7.38	22375
7.4	22341.7
7.42	22391.7
7.44	22675
7.46	22433.3
7.48	22300
7.5	22691.7
7.52	22591.7
7.54	22516.7
7.56	22900
7.58	22966.7
7.6	23175
7.62	23383.3
7.64	23600
7.66	23875
7.68	23741.7
7.7	23750
7.72	24283.3
7.74	23983.3
7.76	23725
7.78	23941.7
7.8	24300

7.1	11033.3
7.12	10583.3
7.14	10416.7
7.16	10566.7
7.18	10575
7.2	10683.3
7.22	10775
7.24	10808.3
7.26	10691.7
7.28	11000
7.3	11066.7
7.32	10750
7.34	10833.3
7.36	10950
7.38	10916.7
7.4	11033.3
7.42	10816.7
7.44	10650
7.46	10725
7.48	10691.7
7.5	11091.7
7.52	11175
7.54	11058.3
7.56	11333.3
7.58	11408.3
7.6	11350
7.62	11200
7.64	11025
7.66	10850
7.68	10925
7.7	11158.3
7.72	11566.7
7.74	11800
7.76	11583.3
7.78	11375
7.8	11241.7

7.1	10600
7.12	10600
7.14	10775
7.16	10833.3
7.18	10933.3
7.2	10616.7
7.22	10700
7.24	10841.7
7.26	10741.7
7.28	10675
7.3	10516.7
7.32	10700
7.34	10741.7
7.36	10441.7
7.38	10683.3
7.4	11016.7
7.42	10925
7.44	10816.7
7.46	10916.7
7.48	11150
7.5	11016.7
7.52	11116.7
7.54	11266.7
7.56	11183.3
7.58	11100
7.6	11050
7.62	11308.3
7.64	11241.7
7.66	10950
7.68	11000
7.7	11100
7.72	11200
7.74	11125
7.76	11166.7
7.78	11425
7.8	11308.3

7.82	11058.3
7.84	11200
7.86	11208.3
7.88	11341.7
7.9	11408.3
7.92	11258.3
7.94	11316.7
7.96	11483.3
7.98	11358.3
8	11125
8.02	11283.3
8.04	11566.7
8.06	11641.7
8.08	11208.3
8.1	10841.7
8.12	11241.7
8.14	11825
8.16	11533.3
8.18	11333.3
8.2	11483.3
8.22	11591.7
8.24	11583.3
8.26	11425
8.28	11633.3
8.3	11683.3
8.32	11233.3
8.34	11191.7
8.36	11666.7
8.38	11966.7
8.4	11950
8.42	11800
8.44	11933.3
8.46	12033.3
8.48	11833.3
8.5	11908.3
8.52	12125

7.82	24566.7
7.84	24291.7
7.86	24291.7
7.88	24183.3
7.9	23958.3
7.92	24425
7.94	24883.3
7.96	24733.3
7.98	24591.7
8	24666.7
8.02	25275
8.04	25091.7
8.06	24533.3
8.08	25083.3
8.1	25391.7
8.12	25875
8.14	26141.7
8.16	25316.7
8.18	25608.3
8.2	25725
8.22	25108.3
8.24	25591.7
8.26	25966.7
8.28	25591.7
8.3	25525
8.32	25725
8.34	25891.7
8.36	26091.7
8.38	26158.3
8.4	26550
8.42	26541.7
8.44	26525
8.46	26958.3
8.48	27416.7
8.5	27400
8.52	26958.3

7.82	10900
7.84	11083.3
7.86	11258.3
7.88	11200
7.9	11325
7.92	11541.7
7.94	11350
7.96	11483.3
7.98	11491.7
8	11075
8.02	11483.3
8.04	11591.7
8.06	11391.7
8.08	11650
8.1	11750
8.12	11558.3
8.14	11341.7
8.16	11450
8.18	11383.3
8.2	11125
8.22	11341.7
8.24	11375
8.26	11591.7
8.28	11558.3
8.3	11366.7
8.32	11400
8.34	11608.3
8.36	11950
8.38	11950
8.4	11800
8.42	11766.7
8.44	12033.3
8.46	11933.3
8.48	11883.3
8.5	11891.7
8.52	11983.3

7.82	11183.3
7.84	11416.7
7.86	11283.3
7.88	10991.7
7.9	11166.7
7.92	11133.3
7.94	11075
7.96	11116.7
7.98	11050
8	10991.7
8.02	11258.3
8.04	11333.3
8.06	11300
8.08	11333.3
8.1	11758.3
8.12	11666.7
8.14	11716.7
8.16	11900
8.18	11483.3
8.2	11500
8.22	11750
8.24	11841.7
8.26	11925
8.28	11650
8.3	11616.7
8.32	11566.7
8.34	11508.3
8.36	11691.7
8.38	11525
8.4	11575
8.42	11891.7
8.44	11941.7
8.46	11958.3
8.48	11991.7
8.5	12208.3
8.52	11975

8.54	12125
8.56	11808.3
8.58	12025
8.6	12100
8.62	12008.3
8.64	12200
8.66	12166.7
8.68	11908.3
8.7	11700
8.72	11750
8.74	11900
8.76	11766.7
8.78	11566.7
8.8	11508.3
8.82	11758.3
8.84	11983.3
8.86	11975
8.88	12033.3
8.9	12108.3
8.92	11991.7
8.94	12041.7
8.96	12050
8.98	12116.7
9	12050
9.02	12200
9.04	12716.7
9.06	12675
9.08	12591.7
9.1	12575
9.12	12466.7
9.14	12441.7
9.16	12308.3
9.18	12166.7
9.2	12658.3
9.22	13050
9.24	12866.7

8.54	27016.7
8.56	27425
8.58	27425
8.6	27175
8.62	27441.7
8.64	27933.3
8.66	28050
8.68	28258.3
8.7	27883.3
8.72	28283.3
8.74	28558.3
8.76	28108.3
8.78	27958.3
8.8	28300
8.82	28716.7
8.84	29125
8.86	28875
8.88	28358.3
8.9	28350
8.92	28675
8.94	28858.3
8.96	29050
8.98	29291.7
9	29391.7
9.02	29608.3
9.04	30233.3
9.06	30408.3
9.08	30075
9.1	30108.3
9.12	30333.3
9.14	29908.3
9.16	29416.7
9.18	29441.7
9.2	29650
9.22	30150
9.24	30091.7

8.54	12133.3
8.56	12275
8.58	12016.7
8.6	11991.7
8.62	12175
8.64	12200
8.66	12308.3
8.68	12083.3
8.7	12183.3
8.72	12116.7
8.74	11858.3
8.76	11916.7
8.78	12075
8.8	11966.7
8.82	11783.3
8.84	11816.7
8.86	12275
8.88	12400
8.9	12158.3
8.92	12266.7
8.94	12233.3
8.96	12100
8.98	12175
9	12216.7
9.02	12300
9.04	12766.7
9.06	12891.7
9.08	12475
9.1	12358.3
9.12	12550
9.14	12766.7
9.16	12841.7
9.18	13041.7
9.2	13325
9.22	13275
9.24	13600

8.54	11975
8.56	12075
8.58	12108.3
8.6	12275
8.62	12408.3
8.64	12558.3
8.66	12425
8.68	12458.3
8.7	12550
8.72	12350
8.74	12325
8.76	12158.3
8.78	11941.7
8.8	12233.3
8.82	12108.3
8.84	12175
8.86	12783.3
8.88	12683.3
8.9	12183.3
8.92	12175
8.94	12441.7
8.96	12516.7
8.98	12116.7
9	12175
9.02	12833.3
9.04	12691.7
9.06	12525
9.08	12508.3
9.1	12658.3
9.12	12725
9.14	12441.7
9.16	12416.7
9.18	12683.3
9.2	12950
9.22	12708.3
9.24	12475

9.26	12433.3
9.28	12191.7
9.3	12291.7
9.32	12691.7
9.34	12975
9.36	12841.7
9.38	13091.7
9.4	13491.7
9.42	13233.3
9.44	13283.3
9.46	13325
9.48	13316.7
9.5	13700
9.52	13800
9.54	13933.3
9.56	14266.7
9.58	14908.3
9.6	15233.3
9.62	15091.7
9.64	15625
9.66	16533.3
9.68	17141.7
9.7	17616.7
9.72	17908.3
9.74	18441.7
9.76	19275
9.78	20125
9.8	21300
9.82	22750
9.84	24100
9.86	25683.3
9.88	27475
9.9	29275
9.92	31683.3
9.94	34616.7
9.96	38000

9.26	30116.7
9.28	30558.3
9.3	30825
9.32	31100
9.34	31075
9.36	31133.3
9.38	31308.3
9.4	31558.3
9.42	31425
9.44	31375
9.46	31508.3
9.48	31650
9.5	32241.7
9.52	32525
9.54	32016.7
9.56	31900
9.58	32516.7
9.6	32900
9.62	32816.7
9.64	32533.3
9.66	32291.7
9.68	32058.3
9.7	32300
9.72	32550
9.74	32991.7
9.76	33658.3
9.78	33858.3
9.8	33866.7
9.82	33408.3
9.84	33641.7
9.86	33966.7
9.88	34041.7
9.9	34208.3
9.92	34150
9.94	34066.7
9.96	34158.3

9.26	13125
9.28	13266.7
9.3	13766.7
9.32	12925
9.34	12725
9.36	13050
9.38	13158.3
9.4	13258.3
9.42	13591.7
9.44	13916.7
9.46	13591.7
9.48	13275
9.5	13208.3
9.52	13641.7
9.54	14225
9.56	14208.3
9.58	14208.3
9.6	14925
9.62	15708.3
9.64	16183.3
9.66	16216.7
9.68	16425
9.7	17575
9.72	18100
9.74	18258.3
9.76	18925
9.78	19633.3
9.8	20658.3
9.82	21691.7
9.84	22916.7
9.86	25025
9.88	27041.7
9.9	28366.7
9.92	30166.7
9.94	32675
9.96	36266.7

9.26	12466.7
9.28	12475
9.3	12708.3
9.32	12608.3
9.34	12908.3
9.36	13291.7
9.38	13408.3
9.4	13741.7
9.42	13616.7
9.44	13341.7
9.46	13633.3
9.48	13616.7
9.5	13466.7
9.52	13383.3
9.54	13450
9.56	13633.3
9.58	14008.3
9.6	14100
9.62	14075
9.64	14483.3
9.66	14975
9.68	15158.3
9.7	14916.7
9.72	14991.7
9.74	15658.3
9.76	16250
9.78	16583.3
9.8	16825
9.82	17150
9.84	17316.7
9.86	17708.3
9.88	18558.3
9.9	19525
9.92	20350
9.94	21091.7
9.96	22075

9.98	42191.7
10	46566.7
10.02	50233.3
10.04	54250
10.06	56416.7
10.08	55950
10.1	54550
10.12	51175
10.14	45675
10.16	39266.7
10.18	33708.3
10.2	29683.3
10.22	27450
10.24	25941.7
10.26	25291.7
10.28	24491.7
10.3	22900
10.32	21525
10.34	20391.7
10.36	19333.3
10.38	18408.3
10.4	17675
10.42	16808.3
10.44	15900
10.46	15958.3
10.48	15991.7
10.5	15391.7
10.52	14708.3
10.54	14858.3
10.56	14941.7
10.58	14625
10.6	15141.7
10.62	15725
10.64	15800
10.66	16183.3
10.68	16575

9.98	34641.7
10	35025
10.02	34983.3
10.04	34741.7
10.06	34450
10.08	34816.7
10.1	35533.3
10.12	35616.7
10.14	35350
10.16	35175
10.18	35850
10.2	36441.7
10.22	35858.3
10.24	35350
10.26	35658.3
10.28	36266.7
10.3	36533.3
10.32	36041.7
10.34	36025
10.36	36908.3
10.38	37391.7
10.4	37216.7
10.42	37033.3
10.44	36733.3
10.46	37358.3
10.48	37983.3
10.5	37625
10.52	37458.3
10.54	37400
10.56	37808.3
10.58	38066.7
10.6	37925
10.62	38625
10.64	38950
10.66	38958.3
10.68	38708.3

9.98	40466.7
10	45350
10.02	50941.7
10.04	56191.7
10.06	61358.3
10.08	65950
10.1	69591.7
10.12	69916.7
10.14	65233.3
10.16	57700
10.18	48416.7
10.2	39058.3
10.22	31666.7
10.24	26716.7
10.26	23650
10.28	21766.7
10.3	21216.7
10.32	21333.3
10.34	20616.7
10.36	19541.7
10.38	18983.3
10.4	18391.7
10.42	17558.3
10.44	16975
10.46	16358.3
10.48	15891.7
10.5	15675
10.52	15333.3
10.54	15400
10.56	14991.7
10.58	14725
10.6	14725
10.62	14725
10.64	15191.7
10.66	16016.7
10.68	16133.3

9.98	22783.3
10	24125
10.02	25975
10.04	27475
10.06	28858.3
10.08	30433.3
10.1	33008.3
10.12	35733.3
10.14	38550
10.16	41375
10.18	44000
10.2	47091.7
10.22	50591.7
10.24	54108.3
10.26	57783.3
10.28	60891.7
10.3	63241.7
10.32	65050
10.34	64858.3
10.36	64275
10.38	62525
10.4	58375
10.42	53075
10.44	47250
10.46	41400
10.48	36675
10.5	33525
10.52	29825
10.54	26708.3
10.56	24333.3
10.58	22350
10.6	21041.7
10.62	20158.3
10.64	19216.7
10.66	18958.3
10.68	18816.7

10.7	16950
10.72	17216.7
10.74	17066.7
10.76	17375
10.78	17608.3
10.8	17850
10.82	18083.3
10.84	18100
10.86	18200
10.88	18758.3
10.9	19258.3
10.92	20116.7
10.94	21175
10.96	22316.7
10.98	23525
11	24358.3
11.02	26033.3
11.04	28158.3
11.06	30350
11.08	32566.7
11.1	35133.3
11.12	37866.7
11.14	40800
11.16	44608.3
11.18	48908.3
11.2	54375
11.22	60350
11.24	66275
11.26	73541.7
11.28	83241.7
11.3	95441.7
11.32	108517
11.34	123992
11.36	144933
11.38	171025
11.4	201108

10.7	38525
10.72	38708.3
10.74	38975
10.76	39708.3
10.78	39425
10.8	39366.7
10.82	39916.7
10.84	40291.7
10.86	39750
10.88	39516.7
10.9	40841.7
10.92	40883.3
10.94	40025
10.96	40508.3
10.98	41375
11	41041.7
11.02	40958.3
11.04	40750
11.06	40525
11.08	41500
11.1	41883.3
11.12	41791.7
11.14	41941.7
11.16	42191.7
11.18	42441.7
11.2	42075
11.22	42258.3
11.24	42991.7
11.26	43275
11.28	43075
11.3	42900
11.32	42933.3
11.34	42816.7
11.36	43258.3
11.38	43108.3
11.4	42916.7

10.7	16225
10.72	16833.3
10.74	16950
10.76	17558.3
10.78	17941.7
10.8	18075
10.82	18525
10.84	18500
10.86	18333.3
10.88	18633.3
10.9	18666.7
10.92	18941.7
10.94	19641.7
10.96	20341.7
10.98	20975
11	21766.7
11.02	22800
11.04	24541.7
11.06	26850
11.08	28108.3
11.1	29958.3
11.12	32441.7
11.14	34608.3
11.16	37275
11.18	39891.7
11.2	43100
11.22	46825
11.24	52008.3
11.26	58633.3
11.28	64758.3
11.3	71150
11.32	78600
11.34	88016.7
11.36	100825
11.38	116750
11.4	135058

10.7	18675
10.72	17850
10.74	17075
10.76	17341.7
10.78	17283.3
10.8	17191.7
10.82	17066.7
10.84	17133.3
10.86	17550
10.88	17608.3
10.9	17950
10.92	17908.3
10.94	17825
10.96	18191.7
10.98	18775
11	19116.7
11.02	19133.3
11.04	19241.7
11.06	19675
11.08	20016.7
11.1	20633.3
11.12	21066.7
11.14	21208.3
11.16	21633.3
11.18	22208.3
11.2	23016.7
11.22	24008.3
11.24	25758.3
11.26	27441.7
11.28	28966.7
11.3	30316.7
11.32	32141.7
11.34	34700
11.36	37333.3
11.38	40583.3
11.4	43550

11.42	234625
11.44	269033
11.46	298408
11.48	318767
11.5	327508
11.52	320850
11.54	297367
11.56	259525
11.58	211092
11.6	161042
11.62	118267
11.64	85516.7
11.66	64075
11.68	50916.7
11.7	41891.7
11.72	35208.3
11.74	29641.7
11.76	25400
11.78	23675
11.8	22841.7
11.82	21750
11.84	20991.7
11.86	20283.3
11.88	19825
11.9	19050
11.92	18600
11.94	18450
11.96	18275
11.98	18191.7
12	18133.3
12.02	18458.3
12.04	18500
12.06	18350
12.08	18366.7
12.1	18458.3
12.12	18516.7

11.42	43300
11.44	43608.3
11.46	44233.3
11.48	44358.3
11.5	44133.3
11.52	44075
11.54	44233.3
11.56	44633.3
11.58	43941.7
11.6	43958.3
11.62	45000
11.64	44633.3
11.66	44841.7
11.68	46175
11.7	46425
11.72	45900
11.74	45366.7
11.76	45458.3
11.78	46358.3
11.8	46358.3
11.82	45916.7
11.84	46458.3
11.86	47125
11.88	46683.3
11.9	46450
11.92	46575
11.94	46833.3
11.96	47533.3
11.98	47816.7
12	48091.7
12.02	48108.3
12.04	47050
12.06	46933.3
12.08	48150
12.1	48475
12.12	48266.7

11.42	158867
11.44	188642
11.46	219567
11.48	247892
11.5	272092
11.52	293150
11.54	306950
11.56	308058
11.58	290450
11.6	254492
11.62	203933
11.64	147942
11.66	102233
11.68	70133.3
11.7	48125
11.72	34925
11.74	27858.3
11.76	23550
11.78	22208.3
11.8	21916.7
11.82	21291.7
11.84	20758.3
11.86	20283.3
11.88	19941.7
11.9	19358.3
11.92	18958.3
11.94	19133.3
11.96	18600
11.98	18025
12	17958.3
12.02	17883.3
12.04	18091.7
12.06	18066.7
12.08	17833.3
12.1	17816.7
12.12	18150

11.42	46708.3
11.44	50925
11.46	55866.7
11.48	61875
11.5	68250
11.52	73850
11.54	81475
11.56	90575
11.58	99325
11.6	108883
11.62	117600
11.64	128475
11.66	139833
11.68	148517
11.7	158142
11.72	166717
11.74	171367
11.76	176008
11.78	175800
11.8	168517
11.82	158508
11.84	143950
11.86	125892
11.88	106767
11.9	89408.3
11.92	73925
11.94	60733.3
11.96	51375
11.98	44416.7
12	38000
12.02	33716.7
12.04	30808.3
12.06	28150
12.08	26225
12.1	25241.7
12.12	24541.7

12.14	18608.3
12.16	18941.7
12.18	19141.7
12.2	19433.3
12.22	19875
12.24	20608.3
12.26	21225
12.28	21633.3
12.3	21991.7
12.32	22550
12.34	23800
12.36	24775
12.38	26175
12.4	28175
12.42	29616.7
12.44	31025
12.46	33608.3
12.48	36841.7
12.5	39308.3
12.52	42275
12.54	46375
12.56	50050
12.58	52625
12.6	54875
12.62	55708.3
12.64	54408.3
12.66	52275
12.68	48533.3
12.7	43666.7
12.72	38666.7
12.74	33541.7
12.76	29075
12.78	25366.7
12.8	22608.3
12.82	20991.7
12.84	19900

12.14	48125
12.16	48125
12.18	48158.3
12.2	48708.3
12.22	48875
12.24	48941.7
12.26	49558.3
12.28	49200
12.3	48925
12.32	49583.3
12.34	50016.7
12.36	50000
12.38	50083.3
12.4	49716.7
12.42	50141.7
12.44	51075
12.46	50866.7
12.48	51158.3
12.5	51091.7
12.52	50566.7
12.54	50466.7
12.56	50983.3
12.58	51700
12.6	51816.7
12.62	51491.7
12.64	51125
12.66	51941.7
12.68	52925
12.7	52375
12.72	51658.3
12.74	52075
12.76	52800
12.78	52775
12.8	52833.3
12.82	52991.7
12.84	52975

12.14	18308.3
12.16	18475
12.18	18675
12.2	19033.3
12.22	19775
12.24	20233.3
12.26	20375
12.28	20875
12.3	21641.7
12.32	22250
12.34	22841.7
12.36	23591.7
12.38	24491.7
12.4	25408.3
12.42	27166.7
12.44	29100
12.46	30916.7
12.48	33433.3
12.5	35841.7
12.52	39516.7
12.54	44200
12.56	48191.7
12.58	52391.7
12.6	56733.3
12.62	59758.3
12.64	61100
12.66	62525
12.68	62291.7
12.7	59250
12.72	54183.3
12.74	47725
12.76	40300
12.78	33675
12.8	27858.3
12.82	23016.7
12.84	20333.3

12.14	23950
12.16	23183.3
12.18	22433.3
12.2	21775
12.22	21100
12.24	20958.3
12.26	20633.3
12.28	20408.3
12.3	20866.7
12.32	20458.3
12.34	20366.7
12.36	20941.7
12.38	21341.7
12.4	21650
12.42	21916.7
12.44	22008.3
12.46	22541.7
12.48	23225
12.5	23750
12.52	24566.7
12.54	25725
12.56	27066.7
12.58	27725
12.6	28516.7
12.62	30441.7
12.64	32400
12.66	34133.3
12.68	36033.3
12.7	38075
12.72	40366.7
12.74	42841.7
12.76	44900
12.78	47316.7
12.8	49566.7
12.82	51208.3
12.84	52783.3

12.86	18775
12.88	17916.7
12.9	17483.3
12.92	17308.3
12.94	17200
12.96	17025
12.98	16875
13	16883.3
13.02	16566.7
13.04	15958.3
13.06	15933.3
13.08	16166.7
13.1	16350
13.12	16391.7
13.14	16158.3
13.16	16150
13.18	16008.3
13.2	15858.3
13.22	16141.7
13.24	16050
13.26	15983.3
13.28	16091.7
13.3	16133.3
13.32	16341.7
13.34	16091.7
13.36	15575
13.38	15816.7
13.4	16066.7
13.42	15950
13.44	15983.3
13.46	16091.7
13.48	15975
13.5	15925
13.52	15816.7
13.54	15850
13.56	15925

12.86	53408.3
12.88	53625
12.9	53991.7
12.92	54058.3
12.94	53608.3
12.96	53991.7
12.98	54466.7
13	54750
13.02	55266.7
13.04	55066.7
13.06	55033.3
13.08	55316.7
13.1	55183.3
13.12	55391.7
13.14	56025
13.16	56350
13.18	56100
13.2	55625
13.22	56058.3
13.24	56683.3
13.26	56791.7
13.28	57458.3
13.3	57400
13.32	57291.7
13.34	58166.7
13.36	58391.7
13.38	58141.7
13.4	58466.7
13.42	58466.7
13.44	58225
13.46	58283.3
13.48	58125
13.5	59166.7
13.52	59366.7
13.54	59183.3
13.56	59425

12.86	18841.7
12.88	18166.7
12.9	18125
12.92	17750
12.94	17516.7
12.96	17366.7
12.98	17058.3
13	16791.7
13.02	16733.3
13.04	16716.7
13.06	16758.3
13.08	16891.7
13.1	16608.3
13.12	16316.7
13.14	16325
13.16	16366.7
13.18	16391.7
13.2	16325
13.22	16358.3
13.24	16333.3
13.26	16025
13.28	16233.3
13.3	16533.3
13.32	16466.7
13.34	16516.7
13.36	16350
13.38	16316.7
13.4	16708.3
13.42	16700
13.44	16391.7
13.46	16325
13.48	16175
13.5	16250
13.52	16208.3
13.54	16066.7
13.56	16016.7

12.86	53408.3
12.88	53758.3
12.9	53100
12.92	51958.3
12.94	50641.7
12.96	48241.7
12.98	44941.7
13	41533.3
13.02	37733.3
13.04	34166.7
13.06	30583.3
13.08	27200
13.1	24991.7
13.12	23350
13.14	22150
13.16	20841.7
13.18	19933.3
13.2	19583.3
13.22	19066.7
13.24	18816.7
13.26	18833.3
13.28	18408.3
13.3	18450
13.32	18841.7
13.34	18341.7
13.36	17858.3
13.38	17850
13.4	17916.7
13.42	17866.7
13.44	17683.3
13.46	17491.7
13.48	17475
13.5	17700
13.52	17950
13.54	18008.3
13.56	17758.3

13.58	15716.7
13.6	15791.7
13.62	16025
13.64	16275
13.66	16316.7
13.68	16366.7
13.7	16208.3
13.72	16158.3
13.74	16600
13.76	16766.7
13.78	16741.7
13.8	16741.7
13.82	16716.7
13.84	16408.3
13.86	16316.7
13.88	17008.3
13.9	17016.7
13.92	16408.3
13.94	15941.7
13.96	15700
13.98	15783.3
14	15875
14.02	15650
14.04	15583.3
14.06	15525
14.08	15250
14.1	15358.3
14.12	16008.3
14.14	16141.7
14.16	15883.3
14.18	15758.3
14.2	15558.3
14.22	15750
14.24	15983.3
14.26	15925
14.28	15700

13.58	59391.7
13.6	60508.3
13.62	60358.3
13.64	59550
13.66	60000
13.68	60916.7
13.7	61558.3
13.72	61208.3
13.74	61050
13.76	61208.3
13.78	61016.7
13.8	61508.3
13.82	61625
13.84	61750
13.86	62608.3
13.88	62233.3
13.9	62050
13.92	63175
13.94	63433.3
13.96	63283.3
13.98	63733.3
14	63675
14.02	63441.7
14.04	63600
14.06	63691.7
14.08	64441.7
14.1	64783.3
14.12	64283.3
14.14	64783.3
14.16	65125
14.18	64375
14.2	64908.3
14.22	65991.7
14.24	65850
14.26	66025
14.28	66450

13.58	16283.3
13.6	16425
13.62	16133.3
13.64	16666.7
13.66	16633.3
13.68	16400
13.7	16175
13.72	16041.7
13.74	16933.3
13.76	17166.7
13.78	16950
13.8	17033.3
13.82	17000
13.84	17116.7
13.86	17116.7
13.88	17066.7
13.9	17125
13.92	16900
13.94	16541.7
13.96	16133.3
13.98	16183.3
14	16758.3
14.02	16708.3
14.04	16391.7
14.06	16066.7
14.08	15641.7
14.1	16083.3
14.12	16233.3
14.14	15841.7
14.16	16000
14.18	16291.7
14.2	16091.7
14.22	15941.7
14.24	15958.3
14.26	15883.3
14.28	15775

13.58	17608.3
13.6	17725
13.62	17858.3
13.64	18208.3
13.66	18508.3
13.68	18191.7
13.7	18041.7
13.72	18141.7
13.74	18241.7
13.76	18541.7
13.78	18116.7
13.8	17608.3
13.82	17500
13.84	17783.3
13.86	17966.7
13.88	17983.3
13.9	18408.3
13.92	18350
13.94	18033.3
13.96	18358.3
13.98	18866.7
14	18733.3
14.02	18258.3
14.04	18375
14.06	18691.7
14.08	18850
14.1	18691.7
14.12	18158.3
14.14	18241.7
14.16	18733.3
14.18	18566.7
14.2	17900
14.22	17791.7
14.24	18066.7
14.26	18383.3
14.28	18425

14.3	15825
14.32	15908.3
14.34	15991.7
14.36	15983.3
14.38	15791.7
14.4	15825
14.42	15525
14.44	15658.3
14.46	15891.7
14.48	15766.7
14.5	15758.3
14.52	15783.3
14.54	15991.7
14.56	16091.7
14.58	16025
14.6	16041.7
14.62	16116.7
14.64	15983.3
14.66	15966.7
14.68	16125
14.7	16016.7
14.72	15933.3
14.74	16158.3
14.76	16425
14.78	16475
14.8	16716.7
14.82	16641.7
14.84	16883.3
14.86	16883.3
14.88	16658.3
14.9	16991.7
14.92	17225
14.94	17325
14.96	16808.3
14.98	16983.3
15	17175

14.3	66208.3
14.32	66775
14.34	67225
14.36	67141.7
14.38	68166.7
14.4	68575
14.42	68450
14.44	67966.7
14.46	67058.3
14.48	67675
14.5	68675
14.52	69008.3
14.54	69391.7
14.56	69416.7
14.58	69766.7
14.6	69383.3
14.62	69116.7
14.64	69258.3
14.66	69783.3
14.68	70516.7
14.7	70783.3
14.72	71366.7
14.74	71516.7
14.76	70775
14.78	70541.7
14.8	71041.7
14.82	71875
14.84	72733.3
14.86	72833.3
14.88	72650
14.9	72816.7
14.92	72916.7
14.94	73116.7
14.96	73958.3
14.98	74275
15	75041.7

14.3	15941.7
14.32	16225
14.34	15925
14.36	15825
14.38	16025
14.4	15891.7
14.42	16091.7
14.44	16066.7
14.46	15950
14.48	16125
14.5	16041.7
14.52	16141.7
14.54	16366.7
14.56	16141.7
14.58	15933.3
14.6	15866.7
14.62	15966.7
14.64	16325
14.66	16358.3
14.68	16233.3
14.7	16091.7
14.72	16500
14.74	16675
14.76	16650
14.78	16675
14.8	16875
14.82	16875
14.84	16891.7
14.86	17241.7
14.88	17475
14.9	17700
14.92	17708.3
14.94	17725
14.96	17941.7
14.98	17925
15	17675

14.3	17741.7
14.32	18008.3
14.34	18333.3
14.36	18208.3
14.38	18716.7
14.4	18633.3
14.42	18283.3
14.44	18408.3
14.46	18400
14.48	18291.7
14.5	18300
14.52	18725
14.54	18725
14.56	18325
14.58	18475
14.6	18325
14.62	18275
14.64	18716.7
14.66	18658.3
14.68	18541.7
14.7	18433.3
14.72	18458.3
14.74	18725
14.76	18675
14.78	18675
14.8	18958.3
14.82	18991.7
14.84	18600
14.86	18141.7
14.88	18025
14.9	18358.3
14.92	18758.3
14.94	18808.3
14.96	19125
14.98	19325
15	19275

15.02	16600
15.04	16641.7
15.06	16941.7
15.08	16808.3
15.1	16941.7
15.12	16958.3
15.14	17141.7
15.16	17533.3
15.18	17691.7
15.2	17825
15.22	17433.3
15.24	17641.7
15.26	17750
15.28	17325
15.3	17516.7
15.32	17825
15.34	18241.7
15.36	19050
15.38	19300
15.4	19450
15.42	19741.7
15.44	20575
15.46	21383.3
15.48	22341.7
15.5	23416.7
15.52	24266.7
15.54	25150
15.56	26483.3
15.58	27266.7
15.6	26983.3
15.62	26791.7
15.64	26258.3
15.66	25683.3
15.68	24408.3
15.7	22858.3
15.72	21250

15.02	75625
15.04	75241.7
15.06	75091.7
15.08	74525
15.1	74408.3
15.12	75108.3
15.14	75691.7
15.16	75966.7
15.18	76758.3
15.2	77116.7
15.22	76375
15.24	76225
15.26	76933.3
15.28	77775
15.3	77675
15.32	78000
15.34	78800
15.36	79083.3
15.38	78941.7
15.4	78825
15.42	79750
15.44	79708.3
15.46	79241.7
15.48	79241.7
15.5	80091.7
15.52	80566.7
15.54	80291.7
15.56	80516.7
15.58	80825
15.6	81375
15.62	81991.7
15.64	82158.3
15.66	82308.3
15.68	82716.7
15.7	82966.7
15.72	83133.3

15.02	17758.3
15.04	17550
15.06	17683.3
15.08	17775
15.1	17300
15.12	17191.7
15.14	17216.7
15.16	17316.7
15.18	17708.3
15.2	17933.3
15.22	18166.7
15.24	18341.7
15.26	17883.3
15.28	17808.3
15.3	18250
15.32	18291.7
15.34	18358.3
15.36	18750
15.38	18883.3
15.4	19283.3
15.42	19750
15.44	19600
15.46	19858.3
15.48	20683.3
15.5	21433.3
15.52	22591.7
15.54	23383.3
15.56	23916.7
15.58	24850
15.6	25891.7
15.62	27008.3
15.64	27525
15.66	28141.7
15.68	27783.3
15.7	26300
15.72	24633.3

15.02	19458.3
15.04	19508.3
15.06	19416.7
15.08	19450
15.1	19633.3
15.12	19841.7
15.14	19925
15.16	19616.7
15.18	19808.3
15.2	20033.3
15.22	19891.7
15.24	20041.7
15.26	20291.7
15.28	20233.3
15.3	20091.7
15.32	20391.7
15.34	20558.3
15.36	20166.7
15.38	20225
15.4	20566.7
15.42	20275
15.44	20116.7
15.46	20083.3
15.48	20000
15.5	20408.3
15.52	20933.3
15.54	21066.7
15.56	21416.7
15.58	21900
15.6	22183.3
15.62	22133.3
15.64	22091.7
15.66	22550
15.68	23250
15.7	23675
15.72	23916.7

15.74	20075
15.76	19633.3
15.78	19050
15.8	18808.3
15.82	18958.3
15.84	19016.7
15.86	19116.7
15.88	19350
15.9	19475
15.92	19833.3
15.94	20208.3
15.96	20775
15.98	21333.3
16	22183.3
16.02	23125
16.04	23641.7
16.06	24333.3
16.08	25125
16.1	26283.3
16.12	28008.3
16.14	29166.7
16.16	30491.7
16.18	32658.3
16.2	34975
16.22	37400
16.24	40825
16.26	45116.7
16.28	49775
16.3	56008.3
16.32	63908.3
16.34	73408.3
16.36	85125
16.38	99200
16.4	116192
16.42	135067
16.44	157267

15.74	83366.7
15.76	83141.7
15.78	83400
15.8	83183.3
15.82	83508.3
15.84	84391.7
15.86	85050
15.88	86083.3
15.9	86183.3
15.92	86066.7
15.94	86550
15.96	86366.7
15.98	86158.3
16	86808.3
16.02	87366.7
16.04	87500
16.06	87958.3
16.08	88350
16.1	87416.7
16.12	87575
16.14	88525
16.16	88825
16.18	89983.3
16.2	90575
16.22	89316.7
16.24	89458.3
16.26	90158.3
16.28	89900
16.3	90141.7
16.32	89758.3
16.34	89241.7
16.36	90141.7
16.38	91091.7
16.4	91791.7
16.42	92083.3
16.44	91525

15.74	22625
15.76	21625
15.78	21041.7
15.8	20175
15.82	19458.3
15.84	19358.3
15.86	19366.7
15.88	19208.3
15.9	19716.7
15.92	20125
15.94	20266.7
15.96	21191.7
15.98	21950
16	22100
16.02	22566.7
16.04	23591.7
16.06	24458.3
16.08	25216.7
16.1	25950
16.12	26783.3
16.14	28041.7
16.16	29400
16.18	30741.7
16.2	32475
16.22	34825
16.24	37650
16.26	41033.3
16.28	45308.3
16.3	50025
16.32	55300
16.34	61825
16.36	69291.7
16.38	78125
16.4	89741.7
16.42	103333
16.44	119225

15.74	24175
15.76	24433.3
15.78	25291.7
15.8	25891.7
15.82	25783.3
15.84	25458.3
15.86	25425
15.88	26016.7
15.9	26300
15.92	26058.3
15.94	25716.7
15.96	25675
15.98	25233.3
16	24683.3
16.02	24433.3
16.04	24341.7
16.06	24308.3
16.08	24133.3
16.1	24466.7
16.12	24700
16.14	24900
16.16	25525
16.18	25841.7
16.2	26216.7
16.22	26891.7
16.24	27841.7
16.26	28933.3
16.28	29883.3
16.3	30933.3
16.32	32500
16.34	34200
16.36	36058.3
16.38	38241.7
16.4	40491.7
16.42	43600
16.44	47225

16.46	181692
16.48	205542
16.5	229042
16.52	248242
16.54	262767
16.56	270842
16.58	265758
16.6	249833
16.62	229433
16.64	204200
16.66	175925
16.68	152492
16.7	134958
16.72	124408
16.74	121250
16.76	123100
16.78	128292
16.8	136183
16.82	145142
16.84	152592
16.86	158992
16.88	162125
16.9	161517
16.92	158958
16.94	152708
16.96	142200
16.98	130458
17	118533
17.02	108075
17.04	104233
17.06	105217
17.08	108283
17.1	116592
17.12	126542
17.14	134375
17.16	140817

16.46	92383.3
16.48	92950
16.5	92683.3
16.52	93666.7
16.54	93658.3
16.56	93833.3
16.58	95200
16.6	95025
16.62	94208.3
16.64	93833.3
16.66	93833.3
16.68	95216.7
16.7	96350
16.72	96400
16.74	95958.3
16.76	96925
16.78	97525
16.8	96966.7
16.82	97033.3
16.84	97058.3
16.86	97083.3
16.88	97625
16.9	98883.3
16.92	99691.7
16.94	100075
16.96	100050
16.98	99983.3
17	99983.3
17.02	99608.3
17.04	99758.3
17.06	101017
17.08	100967
17.1	100408
17.12	101067
17.14	100717
17.16	100592

16.46	138733
16.48	161525
16.5	186017
16.52	209367
16.54	233267
16.56	255908
16.58	272958
16.6	283033
16.62	282667
16.64	268125
16.66	239525
16.68	206175
16.7	172650
16.72	143117
16.74	125567
16.76	118142
16.78	117858
16.8	124233
16.82	131717
16.84	140058
16.86	149500
16.88	158583
16.9	168150
16.92	175267
16.94	178050
16.96	174008
16.98	161958
17	144342
17.02	126025
17.04	110942
17.06	102158
17.08	100450
17.1	104850
17.12	113042
17.14	123567
17.16	135458

16.46	51191.7
16.48	56266.7
16.5	61766.7
16.52	67608.3
16.54	74566.7
16.56	83233.3
16.58	92975
16.6	102800
16.62	113208
16.64	123525
16.66	135008
16.68	147525
16.7	160233
16.72	171992
16.74	182442
16.76	192800
16.78	201017
16.8	207117
16.82	209983
16.84	209792
16.86	205658
16.88	196083
16.9	184783
16.92	173617
16.94	162017
16.96	151467
16.98	143658
17	139183
17.02	137417
17.04	137575
17.06	137733
17.08	138925
17.1	141242
17.12	142092
17.14	144342
17.16	147658

17.18	142842
17.2	139492
17.22	131642
17.24	118408
17.26	100850
17.28	82741.7
17.3	67133.3
17.32	54058.3
17.34	43416.7
17.36	37050
17.38	32791.7
17.4	29491.7
17.42	27925
17.44	27308.3
17.46	26525
17.48	26341.7
17.5	26766.7
17.52	26450
17.54	26100
17.56	25475
17.58	24891.7
17.6	24650
17.62	24625
17.64	24458.3
17.66	24000
17.68	23608.3
17.7	23150
17.72	23341.7
17.74	23750
17.76	23725
17.78	23641.7
17.8	23491.7
17.82	23033.3
17.84	23108.3
17.86	23650
17.88	23808.3

17.18	100517
17.2	100342
17.22	101200
17.24	101958
17.26	101942
17.28	102417
17.3	103383
17.32	103308
17.34	103217
17.36	103608
17.38	103367
17.4	103100
17.42	103983
17.44	104225
17.46	103408
17.48	102708
17.5	103533
17.52	105483
17.54	105650
17.56	105117
17.58	105225
17.6	105183
17.62	105650
17.64	105700
17.66	106267
17.68	107358
17.7	107083
17.72	106508
17.74	106450
17.76	107358
17.78	108208
17.8	107650
17.82	107517
17.84	107875
17.86	108692
17.88	109067

17.18	146158
17.2	154033
17.22	157275
17.24	155450
17.26	146242
17.28	128775
17.3	107083
17.32	84233.3
17.34	63858.3
17.36	48441.7
17.38	38441.7
17.4	32641.7
17.42	29283.3
17.44	27950
17.46	27425
17.48	27058.3
17.5	27208.3
17.52	26808.3
17.54	26650
17.56	26741.7
17.58	26591.7
17.6	26191.7
17.62	26225
17.64	26041.7
17.66	25650
17.68	25450
17.7	24908.3
17.72	24066.7
17.74	24016.7
17.76	24141.7
17.78	23900
17.8	24041.7
17.82	24866.7
17.84	25091.7
17.86	24850
17.88	24691.7

17.18	148542
17.2	147525
17.22	145350
17.24	142308
17.26	138642
17.28	135167
17.3	131342
17.32	128433
17.34	128175
17.36	129142
17.38	130692
17.4	130442
17.42	129483
17.44	127925
17.46	124742
17.48	119600
17.5	112542
17.52	104250
17.54	93741.7
17.56	82608.3
17.58	72016.7
17.6	62508.3
17.62	55325
17.64	49175
17.66	44283.3
17.68	41183.3
17.7	38525
17.72	36608.3
17.74	35500
17.76	34416.7
17.78	33716.7
17.8	33183.3
17.82	32391.7
17.84	32125
17.86	31341.7
17.88	30883.3

17.9	24166.7
17.92	24608.3
17.94	25275
17.96	25941.7
17.98	26733.3
18	27383.3
18.02	28241.7
18.04	28950
18.06	29841.7
18.08	31858.3
18.1	34058.3
18.12	36258.3
18.14	38666.7
18.16	41891.7
18.18	45433.3
18.2	48783.3
18.22	52166.7
18.24	56250
18.26	61041.7
18.28	65741.7
18.3	70691.7
18.32	76633.3
18.34	83650
18.36	91516.7
18.38	99741.7
18.4	106942
18.42	114042
18.44	119533
18.46	120492
18.48	118117
18.5	111475
18.52	100400
18.54	87666.7
18.56	73808.3
18.58	61591.7
18.6	51533.3

17.9	109142
17.92	108875
17.94	108542
17.96	109250
17.98	109467
18	109683
18.02	109600
18.04	109658
18.06	109658
18.08	109333
18.1	108758
18.12	109517
18.14	110717
18.16	110442
18.18	109608
18.2	109533
18.22	110008
18.24	110050
18.26	109700
18.28	110008
18.3	111233
18.32	111900
18.34	111017
18.36	110383
18.38	111392
18.4	112075
18.42	111850
18.44	111750
18.46	112267
18.48	112633
18.5	111892
18.52	111750
18.54	111183
18.56	111358
18.58	112192
18.6	112008

17.9	24841.7
17.92	25558.3
17.94	25775
17.96	26125
17.98	26691.7
18	27183.3
18.02	27791.7
18.04	28650
18.06	29550
18.08	30600
18.1	32258.3
18.12	33783.3
18.14	36016.7
18.16	38341.7
18.18	40691.7
18.2	43908.3
18.22	47166.7
18.24	50625
18.26	53658.3
18.28	56350
18.3	59533.3
18.32	62725
18.34	66775
18.36	70508.3
18.38	74775
18.4	80358.3
18.42	85008.3
18.44	90358.3
18.46	95883.3
18.48	99400
18.5	99483.3
18.52	96108.3
18.54	90000
18.56	81858.3
18.58	70808.3
18.6	58950

17.9	30666.7
17.92	30416.7
17.94	30400
17.96	30333.3
17.98	30425
18	30816.7
18.02	30525
18.04	30191.7
18.06	30550
18.08	30508.3
18.1	31166.7
18.12	31758.3
18.14	32175
18.16	32650
18.18	33075
18.2	33391.7
18.22	33900
18.24	34683.3
18.26	35900
18.28	37250
18.3	38266.7
18.32	40225
18.34	42016.7
18.36	43466.7
18.38	45525
18.4	47575
18.42	49266.7
18.44	51408.3
18.46	53550
18.48	55566.7
18.5	58108.3
18.52	60100
18.54	61966.7
18.56	63841.7
18.58	64691.7
18.6	65300

18.62	43525
18.64	38341.7
18.66	34883.3
18.68	32350
18.7	30916.7
18.72	29983.3
18.74	29983.3
18.76	30375
18.78	30866.7
18.8	32066.7
18.82	32950
18.84	33783.3
18.86	34883.3
18.88	36883.3
18.9	38625
18.92	41133.3
18.94	44900
18.96	48525
18.98	53033.3
19	58525
19.02	64625
19.04	71808.3
19.06	80191.7
19.08	89025
19.1	100125
19.12	112658
19.14	124117
19.16	135833
19.18	146275
19.2	154400
19.22	160867
19.24	166117
19.26	172050
19.28	179483
19.3	190058
19.32	206275

18.62	112592
18.64	111975
18.66	111575
18.68	112050
18.7	111967
18.72	112292
18.74	112433
18.76	112108
18.78	113000
18.8	114142
18.82	114150
18.84	114192
18.86	114042
18.88	114117
18.9	113933
18.92	113933
18.94	113317
18.96	112625
18.98	111775
19	111792
19.02	113175
19.04	113150
19.06	112567
19.08	111958
19.1	112483
19.12	113067
19.14	112392
19.16	111875
19.18	112458
19.2	112367
19.22	113092
19.24	112858
19.26	111892
19.28	112858
19.3	113475
19.32	112442

18.62	49425
18.64	41350
18.66	35583.3
18.68	32541.7
18.7	30800
18.72	30208.3
18.74	30275
18.76	30016.7
18.78	30566.7
18.8	31491.7
18.82	31741.7
18.84	32408.3
18.86	33891.7
18.88	35325
18.9	37333.3
18.92	39491.7
18.94	41700
18.96	44175
18.98	47283.3
19	51291.7
19.02	55208.3
19.04	61058.3
19.06	67833.3
19.08	75425
19.1	84141.7
19.12	93516.7
19.14	105625
19.16	118192
19.18	129767
19.2	140892
19.22	151058
19.24	158058
19.26	162325
19.28	164142
19.3	163150
19.32	164067

18.62	65958.3
18.64	66516.7
18.66	66841.7
18.68	66600
18.7	66575
18.72	65775
18.74	63775
18.76	62033.3
18.78	59666.7
18.8	56733.3
18.82	53433.3
18.84	49841.7
18.86	47041.7
18.88	44408.3
18.9	42150
18.92	40600
18.94	39391.7
18.96	38475
18.98	39000
19	39691.7
19.02	39525
19.04	40308.3
19.06	41716.7
19.08	42775
19.1	44475
19.12	46700
19.14	49233.3
19.16	51791.7
19.18	54608.3
19.2	57833.3
19.22	62283.3
19.24	68066.7
19.26	73700
19.28	79608.3
19.3	84658.3
19.32	90450

19.34	228925
19.36	258175
19.38	292917
19.4	334133
19.42	372967
19.44	405933
19.46	430725
19.48	442367
19.5	438925
19.52	417233
19.54	383050
19.56	333942
19.58	275108
19.6	218117
19.62	164183
19.64	120692
19.66	90400
19.68	69241.7
19.7	55450
19.72	47025
19.74	42075
19.76	39000
19.78	37375
19.8	35741.7
19.82	34566.7
19.84	34016.7
19.86	32908.3
19.88	32550
19.9	32658.3
19.92	32425
19.94	32600
19.96	32966.7
19.98	33591.7
20	35208.3
20.02	37300
20.04	39258.3

19.34	111367
19.36	111417
19.38	112525
19.4	112808
19.42	112317
19.44	112100
19.46	111958
19.48	111883
19.5	111408
19.52	111617
19.54	111858
19.56	111250
19.58	110833
19.6	110792
19.62	111008
19.64	111817
19.66	111983
19.68	111550
19.7	111242
19.72	110700
19.74	111183
19.76	111125
19.78	110525
19.8	110092
19.82	109208
19.84	109017
19.86	109725
19.88	110892
19.9	111867
19.92	111542
19.94	111233
19.96	111067
19.98	109517
20	108983
20.02	110217
20.04	110392

19.34	167275
19.36	174717
19.38	186758
19.4	204917
19.42	228717
19.44	255458
19.46	285408
19.48	315000
19.5	341533
19.52	359633
19.54	363833
19.56	350467
19.58	319750
19.6	272192
19.62	217292
19.64	163975
19.66	118800
19.68	85483.3
19.7	62750
19.72	49450
19.74	42350
19.76	39183.3
19.78	37383.3
19.8	35691.7
19.82	34950
19.84	34391.7
19.86	33983.3
19.88	33900
19.9	33066.7
19.92	32783.3
19.94	33241.7
19.96	33708.3
19.98	34425
20	35675
20.02	36841.7
20.04	37866.7

19.34	97358.3
19.36	104050
19.38	110067
19.4	115992
19.42	122667
19.44	128075
19.46	133292
19.48	139567
19.5	143117
19.52	145483
19.54	149075
19.56	151267
19.58	155042
19.6	158633
19.62	159575
19.64	161908
19.66	165733
19.68	168558
19.7	169775
19.72	171142
19.74	169592
19.76	166217
19.78	160433
19.8	150542
19.82	139683
19.84	127042
19.86	112983
19.88	99700
19.9	87683.3
19.92	76900
19.94	68083.3
19.96	61183.3
19.98	54875
20	50375
20.02	47341.7
20.04	45008.3

20.06	41591.7
20.08	44141.7
20.1	47466.7
20.12	50950
20.14	54600
20.16	58775
20.18	63325
20.2	67391.7
20.22	69450
20.24	69200
20.26	67483.3
20.28	65491.7
20.3	61875
20.32	56208.3
20.34	50825
20.36	46316.7
20.38	42483.3
20.4	39083.3
20.42	36991.7
20.44	36008.3
20.46	35425
20.48	36025
20.5	38200
20.52	39275
20.54	40333.3
20.56	43025
20.58	46475
20.6	50391.7
20.62	55316.7
20.64	60700
20.66	65691.7
20.68	70491.7
20.7	74575
20.72	76625
20.74	78191.7
20.76	77941.7

20.06	109633
20.08	109467
20.1	108925
20.12	108675
20.14	108525
20.16	108367
20.18	109600
20.2	110200
20.22	108917
20.24	108442
20.26	108442
20.28	108192
20.3	108250
20.32	108375
20.34	108508
20.36	108233
20.38	108200
20.4	109058
20.42	108833
20.44	107675
20.46	107908
20.48	108050
20.5	107400
20.52	107767
20.54	108200
20.56	107425
20.58	107083
20.6	107758
20.62	107617
20.64	106642
20.66	106383
20.68	106350
20.7	106167
20.72	106400
20.74	106483
20.76	105700

20.06	39633.3
20.08	41575
20.1	44366.7
20.12	48541.7
20.14	51858.3
20.16	55516.7
20.18	60683.3
20.2	65508.3
20.22	69625
20.24	72850
20.26	75733.3
20.28	76758.3
20.3	75341.7
20.32	71725
20.34	65975
20.36	58908.3
20.38	51941.7
20.4	45733.3
20.42	40558.3
20.44	37791.7
20.46	35766.7
20.48	34375
20.5	34083.3
20.52	34333.3
20.54	35475
20.56	36683.3
20.58	37450
20.6	38783.3
20.62	41575
20.64	44491.7
20.66	46483.3
20.68	49983.3
20.7	54575
20.72	57533.3
20.74	60216.7
20.76	62575

20.06	43383.3
20.08	41841.7
20.1	41508.3
20.12	40783.3
20.14	40108.3
20.16	40425
20.18	41475
20.2	42275
20.22	43333.3
20.24	45050
20.26	46766.7
20.28	48791.7
20.3	50591.7
20.32	52808.3
20.34	55416.7
20.36	57666.7
20.38	58891.7
20.4	60883.3
20.42	63566.7
20.44	64291.7
20.46	65433.3
20.48	67500
20.5	67425
20.52	65908.3
20.54	64675
20.56	63316.7
20.58	60441.7
20.6	56558.3
20.62	53100
20.64	50450
20.66	47908.3
20.68	45300
20.7	44008.3
20.72	43241.7
20.74	42383.3
20.76	41700

20.78	73825
20.8	68591.7
20.82	62291.7
20.84	54758.3
20.86	48158.3
20.88	42791.7
20.9	38575
20.92	36683.3
20.94	35291.7
20.96	35250
20.98	35833.3
21	36575
21.02	37508.3
21.04	38783.3
21.06	40516.7
21.08	42716.7
21.1	45550
21.12	48050
21.14	51083.3
21.16	55341.7
21.18	59308.3
21.2	62683.3
21.22	65691.7
21.24	69783.3
21.26	73816.7
21.28	77141.7
21.3	80591.7
21.32	82325
21.34	84425
21.36	87691.7
21.38	91300
21.4	97341.7
21.42	106633
21.44	118817
21.46	134317
21.48	155092

20.78	105425
20.8	106483
20.82	106533
20.84	106725
20.86	105858
20.88	105183
20.9	105875
20.92	105375
20.94	104458
20.96	103800
20.98	104392
21	104533
21.02	104308
21.04	104225
21.06	104433
21.08	103875
21.1	103650
21.12	104600
21.14	104500
21.16	103758
21.18	103292
21.2	102967
21.22	102942
21.24	102958
21.26	102908
21.28	104083
21.3	103892
21.32	102875
21.34	102883
21.36	102692
21.38	102658
21.4	102967
21.42	102417
21.44	101542
21.46	100800
21.48	100700

20.78	63941.7
20.8	64250
20.82	61400
20.84	56958.3
20.86	52175
20.88	46800
20.9	41850
20.92	37625
20.94	35325
20.96	34750
20.98	34966.7
21	35783.3
21.02	36525
21.04	37025
21.06	38033.3
21.08	39383.3
21.1	41041.7
21.12	42891.7
21.14	44775
21.16	47383.3
21.18	50708.3
21.2	54633.3
21.22	58475
21.24	61666.7
21.26	65283.3
21.28	69300
21.3	74408.3
21.32	78250
21.34	80358.3
21.36	83550
21.38	86766.7
21.4	91216.7
21.42	97291.7
21.44	103867
21.46	112775
21.48	124492

20.78	41666.7
20.8	41841.7
20.82	42091.7
20.84	42458.3
20.86	43675
20.88	44466.7
20.9	44783.3
20.92	45441.7
20.94	45891.7
20.96	46408.3
20.98	46991.7
21	46891.7
21.02	46866.7
21.04	46750
21.06	46225
21.08	45416.7
21.1	43991.7
21.12	43733.3
21.14	43566.7
21.16	42100
21.18	41391.7
21.2	42000
21.22	42925
21.24	43266.7
21.26	43350
21.28	44791.7
21.3	46516.7
21.32	48250
21.34	49783.3
21.36	51666.7
21.38	54758.3
21.4	56541.7
21.42	58225
21.44	60950
21.46	63483.3
21.48	66333.3

21.5	181767
21.52	212125
21.54	249625
21.56	295842
21.58	344883
21.6	397408
21.62	452867
21.64	504975
21.66	547583
21.68	580892
21.7	597725
21.72	592067
21.74	559675
21.76	502733
21.78	432808
21.8	355075
21.82	280708
21.84	217942
21.86	167900
21.88	130683
21.9	104208
21.92	86025
21.94	72875
21.96	62616.7
21.98	55566.7
22	50150
22.02	46575
22.04	44475
22.06	41525
22.08	38625
22.1	37083.3
22.12	35200
22.14	34016.7
22.16	32891.7
22.18	32491.7
22.2	33100

21.5	101425
21.52	101867
21.54	101542
21.56	101492
21.58	101350
21.6	100658
21.62	100358
21.64	100250
21.66	100192
21.68	100150
21.7	99483.3
21.72	99833.3
21.74	99608.3
21.76	99225
21.78	100450
21.8	100058
21.82	98475
21.84	98800
21.86	98758.3
21.88	98425
21.9	99225
21.92	98875
21.94	98475
21.96	98875
21.98	98491.7
22	98033.3
22.02	97891.7
22.04	97250
22.06	97216.7
22.08	97166.7
22.1	96708.3
22.12	95975
22.14	96600
22.16	97883.3
22.18	97241.7
22.2	96091.7

21.5	139992
21.52	159317
21.54	184250
21.56	215942
21.58	250742
21.6	288525
21.62	329767
21.64	376208
21.66	424483
21.68	471808
21.7	516258
21.72	550708
21.74	566358
21.76	554617
21.78	517625
21.8	459000
21.82	383225
21.84	303617
21.86	231617
21.88	174758
21.9	132817
21.92	105292
21.94	87191.7
21.96	72941.7
21.98	63775
22	56483.3
22.02	50691.7
22.04	46408.3
22.06	42891.7
22.08	40375
22.1	38483.3
22.12	37241.7
22.14	35683.3
22.16	34133.3
22.18	33475
22.2	33241.7

21.5	69991.7
21.52	74691.7
21.54	78241.7
21.56	82066.7
21.58	88641.7
21.6	95175
21.62	102842
21.64	112017
21.66	122725
21.68	133833
21.7	145683
21.72	160783
21.74	176625
21.76	193508
21.78	211058
21.8	226867
21.82	244475
21.84	262283
21.86	277275
21.88	291492
21.9	303375
21.92	311108
21.94	314617
21.96	313208
21.98	305692
22	290833
22.02	271842
22.04	247875
22.06	220458
22.08	193592
22.1	168125
22.12	144950
22.14	125658
22.16	109150
22.18	96000
22.2	84341.7

22.22	32316.7
22.24	32141.7
22.26	32358.3
22.28	32375
22.3	32491.7
22.32	32266.7
22.34	32508.3
22.36	33391.7
22.38	34191.7
22.4	35158.3
22.42	36191.7
22.44	37466.7
22.46	39508.3
22.48	42758.3
22.5	45708.3
22.52	49233.3
22.54	54100
22.56	59958.3
22.58	67000
22.6	74550
22.62	83083.3
22.64	92683.3
22.66	102300
22.68	112400
22.7	121500
22.72	126892
22.74	130300
22.76	131200
22.78	127658
22.8	121383
22.82	111958
22.84	102050
22.86	94683.3
22.88	90625
22.9	88108.3
22.92	88375

22.22	96591.7
22.24	97333.3
22.26	96125
22.28	95825
22.3	96175
22.32	95858.3
22.34	95325
22.36	94683.3
22.38	95225
22.4	95000
22.42	94091.7
22.44	94150
22.46	93875
22.48	93541.7
22.5	93800
22.52	94100
22.54	93891.7
22.56	93075
22.58	93291.7
22.6	93808.3
22.62	93216.7
22.64	93050
22.66	92283.3
22.68	92258.3
22.7	92900
22.72	92366.7
22.74	91325
22.76	91408.3
22.78	92200
22.8	92191.7
22.82	92091.7
22.84	92358.3
22.86	91875
22.88	91125
22.9	91733.3
22.92	92025

22.22	32533.3
22.24	32333.3
22.26	32658.3
22.28	32350
22.3	32450
22.32	32891.7
22.34	33050
22.36	32833.3
22.38	32883.3
22.4	33341.7
22.42	34341.7
22.44	35366.7
22.46	36800
22.48	38616.7
22.5	40700
22.52	43341.7
22.54	46025
22.56	49241.7
22.58	53258.3
22.6	59016.7
22.62	65216.7
22.64	71141.7
22.66	79033.3
22.68	87066.7
22.7	94916.7
22.72	104150
22.74	112000
22.76	117125
22.78	120542
22.8	121825
22.82	118908
22.84	112417
22.86	103983
22.88	94841.7
22.9	87316.7
22.92	84308.3

22.22	74958.3
22.24	67916.7
22.26	61283.3
22.28	56100
22.3	52166.7
22.32	48625
22.34	46308.3
22.36	45158.3
22.38	43008.3
22.4	40950
22.42	40191.7
22.44	39408.3
22.46	39000
22.48	38308.3
22.5	37908.3
22.52	37983.3
22.54	38308.3
22.56	38808.3
22.58	39275
22.6	39875
22.62	41175
22.64	42741.7
22.66	43941.7
22.68	45033.3
22.7	46025
22.72	48258.3
22.74	51458.3
22.76	54058.3
22.78	56383.3
22.8	59958.3
22.82	63425
22.84	65275
22.86	68833.3
22.88	72283.3
22.9	74808.3
22.92	78850

22.94	93325
22.96	100300
22.98	105950
23	111308
23.02	117142
23.04	120208
23.06	118933
23.08	114242
23.1	106667
23.12	96591.7
23.14	85450
23.16	74833.3
23.18	64650
23.2	57141.7
23.22	52458.3
23.24	48433.3
23.26	44591.7
23.28	42341.7
23.3	40950
23.32	39450
23.34	37408.3
23.36	35533.3
23.38	34383.3
23.4	33475
23.42	32741.7
23.44	31941.7
23.46	31883.3
23.48	31958.3
23.5	33033.3
23.52	34116.7
23.54	34908.3
23.56	35825
23.58	37475
23.6	40566.7
23.62	44308.3
23.64	48308.3

22.94	91591.7
22.96	91650
22.98	91258.3
23	91016.7
23.02	90425
23.04	90366.7
23.06	90808.3
23.08	90691.7
23.1	90383.3
23.12	89241.7
23.14	88866.7
23.16	88908.3
23.18	88425
23.2	88166.7
23.22	88200
23.24	88666.7
23.26	89566.7
23.28	88983.3
23.3	87908.3
23.32	87933.3
23.34	87908.3
23.36	87550
23.38	87675
23.4	86883.3
23.42	86691.7
23.44	87016.7
23.46	87241.7
23.48	87383.3
23.5	87083.3
23.52	86650
23.54	86075
23.56	85900
23.58	85350
23.6	85750
23.62	86300
23.64	85916.7

22.94	84208.3
22.96	86208.3
22.98	90975
23	98475
23.02	105892
23.04	112192
23.06	118517
23.08	123150
23.1	123725
23.12	119833
23.14	111692
23.16	99850
23.18	86650
23.2	73883.3
23.22	62566.7
23.24	53716.7
23.26	48275
23.28	44316.7
23.3	41450
23.32	39358.3
23.34	37858.3
23.36	37475
23.38	36275
23.4	34791.7
23.42	33658.3
23.44	32175
23.46	31516.7
23.48	31641.7
23.5	31600
23.52	31291.7
23.54	31891.7
23.56	32858.3
23.58	33908.3
23.6	35575
23.62	38258.3
23.64	40900

22.94	82291.7
22.96	84308.3
22.98	85625
23	87508.3
23.02	88491.7
23.04	87983.3
23.06	87700
23.08	86741.7
23.1	85650
23.12	85025
23.14	84358.3
23.16	82908.3
23.18	83016.7
23.2	84025
23.22	84316.7
23.24	85658.3
23.26	87258.3
23.28	86958.3
23.3	86625
23.32	86058.3
23.34	84783.3
23.36	82716.7
23.38	79266.7
23.4	74208.3
23.42	69008.3
23.44	63916.7
23.46	58508.3
23.48	54283.3
23.5	50316.7
23.52	47383.3
23.54	44750
23.56	42441.7
23.58	40783.3
23.6	39308.3
23.62	38583.3
23.64	37408.3

23.66	52191.7
23.68	57825
23.7	66041.7
23.72	75191.7
23.74	85708.3
23.76	96825
23.78	108308
23.8	121483
23.82	133717
23.84	143875
23.86	150967
23.88	153942
23.9	153325
23.92	147133
23.94	136725
23.96	124042
23.98	106708
24	87883.3
24.02	71550
24.04	57750
24.06	47266.7
24.08	39658.3
24.1	35375
24.12	33141.7
24.14	31950
24.16	31091.7
24.18	29933.3
24.2	29200
24.22	29058.3
24.24	28258.3
24.26	27683.3
24.28	28158.3
24.3	27925
24.32	27266.7
24.34	27041.7
24.36	26300

23.66	86016.7
23.68	85116.7
23.7	85558.3
23.72	86100
23.74	85483.3
23.76	85841.7
23.78	85316.7
23.8	84591.7
23.82	85008.3
23.84	85016.7
23.86	84091.7
23.88	84100
23.9	83991.7
23.92	84100
23.94	83700
23.96	83175
23.98	83625
24	83483.3
24.02	82466.7
24.04	82266.7
24.06	82483.3
24.08	82250
24.1	81991.7
24.12	82358.3
24.14	82650
24.16	82958.3
24.18	82308.3
24.2	82266.7
24.22	82241.7
24.24	82241.7
24.26	81833.3
24.28	81400
24.3	81233.3
24.32	81391.7
24.34	81208.3
24.36	81033.3

23.66	43075
23.68	46341.7
23.7	50750
23.72	55916.7
23.74	61666.7
23.76	68100
23.78	76033.3
23.8	85808.3
23.82	95433.3
23.84	105725
23.86	117742
23.88	128767
23.9	137917
23.92	143475
23.94	143992
23.96	139975
23.98	128792
24	112342
24.02	93833.3
24.04	75083.3
24.06	59516.7
24.08	47683.3
24.1	39891.7
24.12	34950
24.14	31941.7
24.16	30625
24.18	29833.3
24.2	29508.3
24.22	29291.7
24.24	29208.3
24.26	28883.3
24.28	27725
24.3	27525
24.32	27233.3
24.34	26841.7
24.36	26483.3

23.66	36308.3
23.68	35808.3
23.7	35866.7
23.72	36100
23.74	36066.7
23.76	36625
23.78	37241.7
23.8	37975
23.82	39966.7
23.84	41650
23.86	43333.3
23.88	45475
23.9	48116.7
23.92	50750
23.94	53191.7
23.96	56466.7
23.98	59416.7
24	62216.7
24.02	66100
24.04	69508.3
24.06	72375
24.08	74525
24.1	75691.7
24.12	77016.7
24.14	76850
24.16	76883.3
24.18	76008.3
24.2	73083.3
24.22	69141.7
24.24	64850
24.26	59975
24.28	55025
24.3	50158.3
24.32	45783.3
24.34	41925
24.36	38975

24.38	25808.3
24.4	26100
24.42	26108.3
24.44	26000
24.46	26175
24.48	25975
24.5	26300
24.52	26416.7
24.54	26066.7
24.56	26058.3
24.58	26100
24.6	26608.3
24.62	26766.7
24.64	26841.7
24.66	26675
24.68	26716.7
24.7	26508.3
24.72	26333.3
24.74	26591.7
24.76	26191.7
24.78	25916.7
24.8	26483.3
24.82	26758.3
24.84	27208.3
24.86	27000
24.88	26691.7
24.9	27008.3
24.92	26808.3
24.94	26908.3
24.96	27500
24.98	27300
25	27650
25.02	28108.3
25.04	28025
25.06	28466.7
25.08	29491.7

24.38	81541.7
24.4	81166.7
24.42	81458.3
24.44	80958.3
24.46	79958.3
24.48	80083.3
24.5	80091.7
24.52	80275
24.54	80525
24.56	80008.3
24.58	79183.3
24.6	78983.3
24.62	79200
24.64	79383.3
24.66	79800
24.68	79975
24.7	78908.3
24.72	78308.3
24.74	78475
24.76	78558.3
24.78	79408.3
24.8	79175
24.82	78133.3
24.84	78366.7
24.86	78983.3
24.88	78733.3
24.9	78158.3
24.92	77158.3
24.94	77433.3
24.96	77925
24.98	76900
25	76808.3
25.02	76891.7
25.04	77416.7
25.06	77475
25.08	76966.7

24.38	26241.7
24.4	26450
24.42	26125
24.44	25750
24.46	25991.7
24.48	26150
24.5	26183.3
24.52	26416.7
24.54	26158.3
24.56	26291.7
24.58	26458.3
24.6	26616.7
24.62	27058.3
24.64	27000
24.66	26658.3
24.68	26441.7
24.7	26675
24.72	26550
24.74	26358.3
24.76	26500
24.78	26766.7
24.8	26483.3
24.82	26191.7
24.84	26616.7
24.86	26833.3
24.88	26841.7
24.9	26833.3
24.92	26875
24.94	27308.3
24.96	27716.7
24.98	27716.7
25	28041.7
25.02	28116.7
25.04	28300
25.06	28650
25.08	29075

24.38	36750
24.4	35283.3
24.42	33733.3
24.44	32916.7
24.46	32750
24.48	31708.3
24.5	30766.7
24.52	30533.3
24.54	30358.3
24.56	29358.3
24.58	29200
24.6	29491.7
24.62	29408.3
24.64	29491.7
24.66	29041.7
24.68	29025
24.7	29133.3
24.72	29100
24.74	28783.3
24.76	28766.7
24.78	28791.7
24.8	29008.3
24.82	29000
24.84	29300
24.86	29591.7
24.88	29333.3
24.9	29183.3
24.92	29033.3
24.94	29025
24.96	28750
24.98	28466.7
25	28116.7
25.02	28533.3
25.04	29133.3
25.06	29500
25.08	29033.3

25.1	30633.3
25.12	31541.7
25.14	33516.7
25.16	35950
25.18	38516.7
25.2	41183.3
25.22	44458.3
25.24	48683.3
25.26	53141.7
25.28	57691.7
25.3	62033.3
25.32	66966.7
25.34	72850
25.36	79141.7
25.38	84408.3
25.4	88066.7
25.42	89541.7
25.44	89158.3
25.46	87291.7
25.48	84133.3
25.5	80583.3
25.52	75841.7
25.54	71466.7
25.56	69016.7
25.58	66491.7
25.6	64816.7
25.62	62508.3
25.64	60558.3
25.66	58500
25.68	56383.3
25.7	54391.7
25.72	52741.7
25.74	49883.3
25.76	47283.3
25.78	46300
25.8	45458.3

25.1	76700
25.12	76316.7
25.14	76475
25.16	77008.3
25.18	76408.3
25.2	76166.7
25.22	75925
25.24	75683.3
25.26	75800
25.28	75450
25.3	75225
25.32	74766.7
25.34	73766.7
25.36	74483.3
25.38	74766.7
25.4	75133.3
25.42	76091.7
25.44	75658.3
25.46	75300
25.48	74925
25.5	74741.7
25.52	75141.7
25.54	74641.7
25.56	74266.7
25.58	74225
25.6	74266.7
25.62	74700
25.64	74550
25.66	74033.3
25.68	73591.7
25.7	73016.7
25.72	72866.7
25.74	73425
25.76	73400
25.78	72758.3
25.8	73841.7

25.1	29533.3
25.12	30283.3
25.14	31600
25.16	32841.7
25.18	34175
25.2	36400
25.22	39225
25.24	42458.3
25.26	45908.3
25.28	49891.7
25.3	54508.3
25.32	59666.7
25.34	65150
25.36	71508.3
25.38	77691.7
25.4	83791.7
25.42	89058.3
25.44	92483.3
25.46	95275
25.48	96075
25.5	94533.3
25.52	91700
25.54	87366.7
25.56	82325
25.58	77775
25.6	73916.7
25.62	72058.3
25.64	70783.3
25.66	68583.3
25.68	66983.3
25.7	64858.3
25.72	61925
25.74	59225
25.76	56041.7
25.78	53566.7
25.8	51191.7

25.1	29166.7
25.12	29816.7
25.14	30266.7
25.16	30441.7
25.18	30291.7
25.2	30058.3
25.22	30558.3
25.24	31366.7
25.26	31791.7
25.28	32800
25.3	33425
25.32	34558.3
25.34	35758.3
25.36	36833.3
25.38	38791.7
25.4	40616.7
25.42	42941.7
25.44	44908.3
25.46	46783.3
25.48	48825
25.5	51725
25.52	54475
25.54	57016.7
25.56	58808.3
25.58	61625
25.6	65616.7
25.62	67483.3
25.64	69508.3
25.66	71358.3
25.68	71241.7
25.7	71016.7
25.72	71433.3
25.74	71408.3
25.76	70941.7
25.78	69300
25.8	67166.7

25.82	44708.3
25.84	45083.3
25.86	46408.3
25.88	48750
25.9	51716.7
25.92	55133.3
25.94	59150
25.96	63650
25.98	68583.3
26	72733.3
26.02	76116.7
26.04	79733.3
26.06	82933.3
26.08	86225
26.1	88516.7
26.12	88450
26.14	87200
26.16	86541.7
26.18	88050
26.2	90050
26.22	95991.7
26.24	104583
26.26	115375
26.28	129067
26.3	145283
26.32	166100
26.34	189358
26.36	215358
26.38	243783
26.4	271500
26.42	299117
26.44	323108
26.46	341442
26.48	356408
26.5	361642
26.52	355933

25.82	73733.3
25.84	73041.7
25.86	73366.7
25.88	73225
25.9	72975
25.92	73233.3
25.94	73383.3
25.96	73483.3
25.98	72966.7
26	72125
26.02	71783.3
26.04	72166.7
26.06	72691.7
26.08	72525
26.1	72325
26.12	72158.3
26.14	72075
26.16	72091.7
26.18	71625
26.2	72241.7
26.22	71741.7
26.24	71041.7
26.26	71825
26.28	71966.7
26.3	71508.3
26.32	70800
26.34	72050
26.36	70525
26.38	69475
26.4	70500
26.42	71475
26.44	72158.3
26.46	72491.7
26.48	72583.3
26.5	72825
26.52	72483.3

25.82	49083.3
25.84	48608.3
25.86	48191.7
25.88	48025
25.9	49525
25.92	52033.3
25.94	55691.7
25.96	59483.3
25.98	62991.7
26	67283.3
26.02	73166.7
26.04	78941.7
26.06	83183.3
26.08	87958.3
26.1	93583.3
26.12	97641.7
26.14	99508.3
26.16	100142
26.18	98000
26.2	95608.3
26.22	95916.7
26.24	97550
26.26	102000
26.28	108792
26.3	118817
26.32	132683
26.34	150442
26.36	169383
26.38	190500
26.4	213033
26.42	236967
26.44	266517
26.46	296225
26.48	324800
26.5	352250
26.52	373233

25.82	66191.7
25.84	65850
25.86	63941.7
25.88	61883.3
25.9	61291.7
25.92	60458.3
25.94	59016.7
25.96	57691.7
25.98	57141.7
26	55983.3
26.02	54166.7
26.04	53350
26.06	53516.7
26.08	53066.7
26.1	53666.7
26.12	54725
26.14	56008.3
26.16	57000
26.18	59216.7
26.2	62616.7
26.22	65508.3
26.24	67708.3
26.26	69825
26.28	73858.3
26.3	77983.3
26.32	80250
26.34	82483.3
26.36	85650
26.38	88366.7
26.4	91250
26.42	94408.3
26.44	97833.3
26.46	101742
26.48	106650
26.5	111633
26.52	117825

26.54	337150
26.56	306175
26.58	269833
26.6	230517
26.62	192917
26.64	163125
26.66	140550
26.68	126700
26.7	119658
26.72	119708
26.74	123258
26.76	127475
26.78	131858
26.8	132667
26.82	129400
26.84	125092
26.86	119533
26.88	109842
26.9	100358
26.92	90275
26.94	79575
26.96	70875
26.98	63208.3
27	57141.7
27.02	53541.7
27.04	52000
27.06	51766.7
27.08	52416.7
27.1	55066.7
27.12	58808.3
27.14	62591.7
27.16	67991.7
27.18	74366.7
27.2	81933.3
27.22	89333.3
27.24	97683.3

26.54	72591.7
26.56	72450
26.58	72650
26.6	72866.7
26.62	72516.7
26.64	71450
26.66	70516.7
26.68	71091.7
26.7	71958.3
26.72	70766.7
26.74	69566.7
26.76	69008.3
26.78	68991.7
26.8	69575
26.82	68991.7
26.84	68100
26.86	68133.3
26.88	68933.3
26.9	68841.7
26.92	67341.7
26.94	67241.7
26.96	67716.7
26.98	67866.7
27	67708.3
27.02	67275
27.04	67216.7
27.06	67958.3
27.08	68683.3
27.1	68300
27.12	67433.3
27.14	67825
27.16	67700
27.18	67066.7
27.2	66883.3
27.22	66516.7
27.24	66375

26.54	386417
26.56	381900
26.58	358025
26.6	320358
26.62	274192
26.64	225567
26.66	182000
26.68	149733
26.7	126683
26.72	113733
26.74	109100
26.76	111458
26.78	115775
26.8	120533
26.82	124550
26.84	126942
26.86	128167
26.88	124625
26.9	118683
26.92	110117
26.94	99158.3
26.96	87091.7
26.98	78083.3
27	69633.3
27.02	61600
27.04	56233.3
27.06	52475
27.08	50833.3
27.1	50325
27.12	50216.7
27.14	52291.7
27.16	55525
27.18	58766.7
27.2	62591.7
27.22	66825
27.24	72458.3

26.54	127375
26.56	137558
26.58	149583
26.6	160858
26.62	171575
26.64	184683
26.66	194650
26.68	204842
26.7	214492
26.72	223792
26.74	229575
26.76	229542
26.78	228567
26.8	223517
26.82	213867
26.84	200175
26.86	183125
26.88	166600
26.9	152067
26.92	137358
26.94	124867
26.96	114592
26.98	107608
27	103683
27.02	100700
27.04	97966.7
27.06	96033.3
27.08	94341.7
27.1	91916.7
27.12	87983.3
27.14	83141.7
27.16	79741.7
27.18	75883.3
27.2	70625
27.22	66125
27.24	62516.7

27.26	105142
27.28	111775
27.3	118625
27.32	122150
27.34	123583
27.36	124092
27.38	121475
27.4	114275
27.42	104725
27.44	94683.3
27.46	84441.7
27.48	73391.7
27.5	62875
27.52	54466.7
27.54	46975
27.56	41941.7
27.58	38758.3
27.6	35708.3
27.62	33566.7
27.64	32275
27.66	31891.7
27.68	31433.3
27.7	31183.3
27.72	31325
27.74	31650
27.76	31291.7
27.78	31866.7
27.8	32275
27.82	32875
27.84	34516.7
27.86	36250
27.88	38041.7
27.9	40358.3
27.92	43083.3
27.94	46058.3
27.96	49475

27.26	67333.3
27.28	66750
27.3	66925
27.32	67391.7
27.34	66750
27.36	67058.3
27.38	67200
27.4	66966.7
27.42	66016.7
27.44	65625
27.46	66233.3
27.48	65841.7
27.5	65858.3
27.52	65925
27.54	64883.3
27.56	64633.3
27.58	65050
27.6	65591.7
27.62	66100
27.64	65983.3
27.66	65408.3
27.68	65191.7
27.7	65408.3
27.72	64975
27.74	64991.7
27.76	65241.7
27.78	65108.3
27.8	65450
27.82	65275
27.84	65241.7
27.86	65358.3
27.88	65550
27.9	64925
27.92	64916.7
27.94	64941.7
27.96	64316.7

27.26	78750
27.28	85358.3
27.3	92150
27.32	98316.7
27.34	104375
27.36	108775
27.38	110975
27.4	110875
27.42	108625
27.44	103367
27.46	95666.7
27.48	86600
27.5	76666.7
27.52	67625
27.54	59233.3
27.56	51550
27.58	45325
27.6	40125
27.62	35625
27.64	32925
27.66	32025
27.68	31666.7
27.7	31425
27.72	31450
27.74	31191.7
27.76	30991.7
27.78	31258.3
27.8	32325
27.82	33016.7
27.84	33250
27.86	34633.3
27.88	35891.7
27.9	36683.3
27.92	39000
27.94	41241.7
27.96	44016.7

27.26	59741.7
27.28	57575
27.3	55933.3
27.32	54158.3
27.34	53075
27.36	52541.7
27.38	53508.3
27.4	54975
27.42	56125
27.44	57416.7
27.46	59183.3
27.48	61016.7
27.5	62041.7
27.52	63658.3
27.54	65000
27.56	65816.7
27.58	66875
27.6	66608.3
27.62	66500
27.64	66458.3
27.66	65400
27.68	63208.3
27.7	61141.7
27.72	59166.7
27.74	56550
27.76	52608.3
27.78	49691.7
27.8	47050
27.82	43625
27.84	41191.7
27.86	39383.3
27.88	37958.3
27.9	36416.7
27.92	34800
27.94	34333.3
27.96	34316.7

27.98	53400
28	58641.7
28.02	63600
28.04	69658.3
28.06	75458.3
28.08	81416.7
28.1	87683.3
28.12	93675
28.14	98533.3
28.16	101400
28.18	102300
28.2	102833
28.22	100692
28.24	94200
28.26	85333.3
28.28	76175
28.3	67708.3
28.32	59625
28.34	53325
28.36	47925
28.38	43608.3
28.4	41633.3
28.42	40891.7
28.44	41591.7
28.46	42841.7
28.48	44058.3
28.5	45633.3
28.52	47708.3
28.54	50541.7
28.56	53633.3
28.58	56608.3
28.6	58483.3
28.62	60708.3
28.64	63225
28.66	64258.3
28.68	64683.3

27.98	63866.7
28	63641.7
28.02	64308.3
28.04	64200
28.06	63883.3
28.08	64066.7
28.1	63633.3
28.12	63733.3
28.14	64175
28.16	63250
28.18	62516.7
28.2	63850
28.22	64883.3
28.24	64816.7
28.26	63800
28.28	63541.7
28.3	63808.3
28.32	63550
28.34	63508.3
28.36	63150
28.38	63058.3
28.4	63675
28.42	63366.7
28.44	62716.7
28.46	62891.7
28.48	63008.3
28.5	63158.3
28.52	63391.7
28.54	62641.7
28.56	62358.3
28.58	62375
28.6	63000
28.62	62958.3
28.64	62541.7
28.66	62566.7
28.68	63000

27.98	46775
28	50200
28.02	54266.7
28.04	57791.7
28.06	62975
28.08	67791.7
28.1	72125
28.12	77558.3
28.14	84041.7
28.16	90566.7
28.18	94108.3
28.2	95450
28.22	95975
28.24	94150
28.26	90650
28.28	86050
28.3	77941.7
28.32	68416.7
28.34	60791.7
28.36	54166.7
28.38	48175
28.4	44150
28.42	42166.7
28.44	40875
28.46	40508.3
28.48	41525
28.5	44041.7
28.52	45841.7
28.54	47466.7
28.56	50941.7
28.58	53691.7
28.6	57508.3
28.62	60958.3
28.64	64250
28.66	67383.3
28.68	69433.3

27.98	34366.7
28	34833.3
28.02	35175
28.04	35808.3
28.06	36575
28.08	37033.3
28.1	38383.3
28.12	39241.7
28.14	40475
28.16	42991.7
28.18	45300
28.2	46691.7
28.22	48700
28.24	51083.3
28.26	52775
28.28	54575
28.3	56008.3
28.32	57791.7
28.34	59158.3
28.36	60025
28.38	61416.7
28.4	62125
28.42	62983.3
28.44	62533.3
28.46	61416.7
28.48	59466.7
28.5	57016.7
28.52	54650
28.54	53308.3
28.56	50900
28.58	48533.3
28.6	46716.7
28.62	45683.3
28.64	44825
28.66	44541.7
28.68	44925

28.7	64075
28.72	62375
28.74	59850
28.76	57008.3
28.78	54283.3
28.8	52775
28.82	52300
28.84	52016.7
28.86	51933.3
28.88	52850
28.9	55366.7
28.92	60108.3
28.94	67083.3
28.96	74150
28.98	82508.3
29	93425
29.02	107142
29.04	122808
29.06	141550
29.08	161567
29.1	182500
29.12	204583
29.14	223717
29.16	243683
29.18	261175
29.2	271725
29.22	273750
29.24	270825
29.26	259350
29.28	240142
29.3	216192
29.32	187525
29.34	159608
29.36	133017
29.38	109267
29.4	91025

28.7	62966.7
28.72	62191.7
28.74	62558.3
28.76	62291.7
28.78	62100
28.8	62450
28.82	61933.3
28.84	62908.3
28.86	62816.7
28.88	61933.3
28.9	62275
28.92	61700
28.94	61325
28.96	61658.3
28.98	61333.3
29	61141.7
29.02	61125
29.04	61333.3
29.06	60941.7
29.08	60741.7
29.1	60425
29.12	60716.7
29.14	61066.7
29.16	60325
29.18	60025
29.2	60758.3
29.22	60550
29.24	60133.3
29.26	60325
29.28	60016.7
29.3	60608.3
29.32	60658.3
29.34	59883.3
29.36	59908.3
29.38	60166.7
29.4	59116.7

28.7	70750
28.72	71675
28.74	69775
28.76	66783.3
28.78	63266.7
28.8	58375
28.82	55808.3
28.84	53391.7
28.86	51041.7
28.88	49916.7
28.9	50200
28.92	51300
28.94	52408.3
28.96	54691.7
28.98	58716.7
29	65041.7
29.02	71691.7
29.04	78533.3
29.06	87475
29.08	97433.3
29.1	108092
29.12	119675
29.14	134625
29.16	149950
29.18	164733
29.2	179292
29.22	193692
29.24	205892
29.26	213358
29.28	215450
29.3	210933
29.32	200233
29.34	181325
29.36	159375
29.38	133550
29.4	110692

28.7	45116.7
28.72	45008.3
28.74	46041.7
28.76	47100
28.78	48216.7
28.8	50691.7
28.82	51925
28.84	53908.3
28.86	55941.7
28.88	57275
28.9	59191.7
28.92	60016.7
28.94	59808.3
28.96	59508.3
28.98	59333.3
29	58608.3
29.02	57583.3
29.04	56016.7
29.06	54416.7
29.08	53283.3
29.1	52675
29.12	52183.3
29.14	51775
29.16	52225
29.18	53275
29.2	55583.3
29.22	57158.3
29.24	59766.7
29.26	63775
29.28	66358.3
29.3	69308.3
29.32	73491.7
29.34	77275
29.36	80550
29.38	83066.7
29.4	86141.7

29.42	76316.7
29.44	64625
29.46	56458.3
29.48	49616.7
29.5	44800
29.52	41475
29.54	39533.3
29.56	38041.7
29.58	37225
29.6	37325
29.62	36233.3
29.64	35475
29.66	35633.3
29.68	35425
29.7	35041.7
29.72	34516.7
29.74	33716.7
29.76	33325
29.78	32383.3
29.8	32450
29.82	32616.7
29.84	33050
29.86	32758.3
29.88	33158.3
29.9	34958.3
29.92	36750
29.94	38583.3
29.96	41233.3
29.98	43900
30	47558.3
30.02	51966.7
30.04	56408.3
30.06	61775
30.08	68558.3
30.1	75766.7
30.12	81991.7

29.42	58900
29.44	59675
29.46	59525
29.48	59458.3
29.5	60083.3
29.52	59700
29.54	59150
29.56	59216.7
29.58	58925
29.6	58400
29.62	59116.7
29.64	59900
29.66	59741.7
29.68	59391.7
29.7	59475
29.72	59325
29.74	59283.3
29.76	58266.7
29.78	57991.7
29.8	58891.7
29.82	58116.7
29.84	58558.3
29.86	58733.3
29.88	57783.3
29.9	58008.3
29.92	58016.7
29.94	57983.3
29.96	58991.7
29.98	58616.7
30	58116.7
30.02	57925
30.04	57616.7
30.06	57575
30.08	57233.3
30.1	56816.7
30.12	57175

29.42	92975
29.44	78458.3
29.46	65091.7
29.48	55583.3
29.5	49600
29.52	45525
29.54	41766.7
29.56	39150
29.58	37258.3
29.6	36433.3
29.62	36250
29.64	36300
29.66	35383.3
29.68	35341.7
29.7	35775
29.72	36016.7
29.74	35491.7
29.76	34341.7
29.78	33441.7
29.8	33416.7
29.82	32616.7
29.84	32316.7
29.86	32400
29.88	32200
29.9	32650
29.92	33741.7
29.94	35591.7
29.96	36833.3
29.98	38750
30	41441.7
30.02	44333.3
30.04	48375
30.06	51908.3
30.08	56441.7
30.1	62333.3
30.12	68125

29.42	88383.3
29.44	90400
29.46	92816.7
29.48	92566.7
29.5	91633.3
29.52	90041.7
29.54	87041.7
29.56	82683.3
29.58	77850
29.6	72475
29.62	66725
29.64	61608.3
29.66	57475
29.68	53108.3
29.7	49008.3
29.72	46850
29.74	44191.7
29.76	42266.7
29.78	40950
29.8	39150
29.82	38091.7
29.84	38200
29.86	38100
29.88	37775
29.9	37550
29.92	37733.3
29.94	38425
29.96	39533.3
29.98	39591.7
30	39958.3
30.02	41283.3
30.04	42083.3
30.06	43291.7
30.08	45041.7
30.1	47300
30.12	49808.3

30.14	88500
30.16	94433.3
30.18	100900
30.2	107500
30.22	112183
30.24	113508
30.26	113933
30.28	113908
30.3	111900
30.32	107908
30.34	102617
30.36	96108.3
30.38	88066.7
30.4	81166.7
30.42	74458.3
30.44	69116.7
30.46	65600
30.48	64200
30.5	63425
30.52	64183.3
30.54	67008.3
30.56	69008.3
30.58	72375
30.6	74825
30.62	75391.7
30.64	76200
30.66	76808.3
30.68	75141.7
30.7	71100
30.72	66125
30.74	59641.7
30.76	53933.3
30.78	48725
30.8	43608.3
30.82	39741.7
30.84	36608.3

30.14	56725
30.16	56408.3
30.18	56891.7
30.2	57025
30.22	56450
30.24	56775
30.26	57225
30.28	57816.7
30.3	57791.7
30.32	57566.7
30.34	57158.3
30.36	56741.7
30.38	56575
30.4	55666.7
30.42	55750
30.44	56350
30.46	57150
30.48	56658.3
30.5	56616.7
30.52	56483.3
30.54	56391.7
30.56	56541.7
30.58	56983.3
30.6	56908.3
30.62	56075
30.64	55775
30.66	55983.3
30.68	55600
30.7	55416.7
30.72	55575
30.74	55708.3
30.76	56441.7
30.78	56066.7
30.8	55166.7
30.82	54883.3
30.84	55658.3

30.14	74308.3
30.16	80875
30.18	87700
30.2	94008.3
30.22	99958.3
30.24	105150
30.26	110208
30.28	112550
30.3	113475
30.32	113967
30.34	112075
30.36	108950
30.38	104600
30.4	98733.3
30.42	90533.3
30.44	81225
30.46	73500
30.48	67233.3
30.5	62991.7
30.52	61250
30.54	59608.3
30.56	59758.3
30.58	60216.7
30.6	61500
30.62	64425
30.64	67308.3
30.66	68800
30.68	69083.3
30.7	69891.7
30.72	68600
30.74	64183.3
30.76	59741.7
30.78	54283.3
30.8	48691.7
30.82	43716.7
30.84	39658.3

30.14	51708.3
30.16	53591.7
30.18	55758.3
30.2	57666.7
30.22	59491.7
30.24	62633.3
30.26	65208.3
30.28	66516.7
30.3	68583.3
30.32	70508.3
30.34	71616.7
30.36	73116.7
30.38	73558.3
30.4	74775
30.42	75475
30.44	76916.7
30.46	78441.7
30.48	77966.7
30.5	78625
30.52	78258.3
30.54	76691.7
30.56	76300
30.58	74775
30.6	74550
30.62	72441.7
30.64	69658.3
30.66	66250
30.68	63133.3
30.7	59758.3
30.72	57100
30.74	55558.3
30.76	54083.3
30.78	52966.7
30.8	51916.7
30.82	51000
30.84	49983.3

30.86	33533.3
30.88	32408.3
30.9	31241.7
30.92	30733.3
30.94	30016.7
30.96	29683.3
30.98	29733.3
31	29675
31.02	29983.3
31.04	29275
31.06	29225
31.08	28983.3
31.1	29208.3
31.12	29250
31.14	29158.3
31.16	28758.3
31.18	28358.3
31.2	27950
31.22	27658.3
31.24	28041.7
31.26	28841.7
31.28	29591.7
31.3	30216.7
31.32	30450
31.34	31175
31.36	31975
31.38	32658.3
31.4	33733.3
31.42	33633.3
31.44	33725
31.46	33741.7
31.48	34216.7
31.5	34383.3
31.52	34583.3
31.54	34508.3
31.56	34350

30.86	55183.3
30.88	54733.3
30.9	55258.3
30.92	55533.3
30.94	55975
30.96	55500
30.98	55683.3
31	55175
31.02	54333.3
31.04	55008.3
31.06	55316.7
31.08	55558.3
31.1	55200
31.12	54491.7
31.14	54741.7
31.16	54933.3
31.18	55325
31.2	55175
31.22	54550
31.24	54691.7
31.26	54100
31.28	54550
31.3	54333.3
31.32	53800
31.34	54641.7
31.36	55266.7
31.38	55350
31.4	54333.3
31.42	54558.3
31.44	54591.7
31.46	54200
31.48	53750
31.5	53708.3
31.52	53950
31.54	53858.3
31.56	54508.3

30.86	36566.7
30.88	34058.3
30.9	33266.7
30.92	32416.7
30.94	31466.7
30.96	31400
30.98	31450
31	30950
31.02	30758.3
31.04	30050
31.06	30000
31.08	29858.3
31.1	29991.7
31.12	30175
31.14	29541.7
31.16	29783.3
31.18	29608.3
31.2	28925
31.22	28491.7
31.24	28908.3
31.26	29291.7
31.28	29316.7
31.3	29175
31.32	29741.7
31.34	30433.3
31.36	30983.3
31.38	31250
31.4	31791.7
31.42	32666.7
31.44	33333.3
31.46	34041.7
31.48	34258.3
31.5	34650
31.52	34391.7
31.54	34600
31.56	34441.7

30.86	48466.7
30.88	47983.3
30.9	47783.3
30.92	46183.3
30.94	45608.3
30.96	44850
30.98	43441.7
31	41775
31.02	39800
31.04	38483.3
31.06	37516.7
31.08	36083.3
31.1	34675
31.12	34200
31.14	33400
31.16	32608.3
31.18	32200
31.2	32175
31.22	31500
31.24	30375
31.26	30150
31.28	30033.3
31.3	29300
31.32	28991.7
31.34	29450
31.36	29608.3
31.38	29425
31.4	29150
31.42	29416.7
31.44	29433.3
31.46	29508.3
31.48	29600
31.5	29600
31.52	29316.7
31.54	29466.7
31.56	29033.3

31.58	34300
31.6	34083.3
31.62	33150
31.64	32366.7
31.66	31808.3
31.68	30375
31.7	30008.3
31.72	29066.7
31.74	27358.3
31.76	27133.3
31.78	26458.3
31.8	25750
31.82	25000
31.84	24458.3
31.86	24250
31.88	23750
31.9	23733.3
31.92	23500
31.94	23575
31.96	23408.3
31.98	23850
32	23891.7
32.02	23800
32.04	23616.7
32.06	23400
32.08	23866.7
32.1	24008.3
32.12	23691.7
32.14	23241.7
32.16	24100
32.18	24608.3
32.2	25291.7
32.22	26200
32.24	27266.7
32.26	28825
32.28	29591.7

31.58	54933.3
31.6	54225
31.62	54500
31.64	53991.7
31.66	53883.3
31.68	53833.3
31.7	53616.7
31.72	53633.3
31.74	53600
31.76	53608.3
31.78	53191.7
31.8	53250
31.82	53208.3
31.84	52941.7
31.86	53533.3
31.88	53058.3
31.9	52391.7
31.92	52791.7
31.94	53183.3
31.96	53066.7
31.98	53433.3
32	53725
32.02	52633.3
32.04	53133.3
32.06	53300
32.08	52875
32.1	52525
32.12	52133.3
32.14	52375
32.16	52658.3
32.18	52950
32.2	52633.3
32.22	52566.7
32.24	53358.3
32.26	53400
32.28	52983.3

31.58	34366.7
31.6	34108.3
31.62	33783.3
31.64	33458.3
31.66	33000
31.68	32600
31.7	31658.3
31.72	30716.7
31.74	29866.7
31.76	29483.3
31.78	28091.7
31.8	27633.3
31.82	27058.3
31.84	26491.7
31.86	25941.7
31.88	25058.3
31.9	24433.3
31.92	24883.3
31.94	24525
31.96	23625
31.98	23300
32	23608.3
32.02	23641.7
32.04	23641.7
32.06	23408.3
32.08	23366.7
32.1	23641.7
32.12	23508.3
32.14	24075
32.16	24183.3
32.18	24658.3
32.2	25166.7
32.22	25441.7
32.24	26500
32.26	27641.7
32.28	28625

31.58	29100
31.6	30408.3
31.62	31066.7
31.64	30883.3
31.66	31316.7
31.68	31166.7
31.7	31375
31.72	31716.7
31.74	31933.3
31.76	31841.7
31.78	31875
31.8	31775
31.82	31066.7
31.84	30183.3
31.86	29100
31.88	28708.3
31.9	29100
31.92	28216.7
31.94	28216.7
31.96	27791.7
31.98	27541.7
32	26950
32.02	26875
32.04	26766.7
32.06	25883.3
32.08	25825
32.1	25916.7
32.12	25650
32.14	24783.3
32.16	24841.7
32.18	24800
32.2	24700
32.22	24533.3
32.24	24300
32.26	24200
32.28	24183.3

32.3	31258.3
32.32	33475
32.34	35158.3
32.36	37358.3
32.38	39350
32.4	42008.3
32.42	44283.3
32.44	46541.7
32.46	48483.3
32.48	50150
32.5	51841.7
32.52	51975
32.54	52866.7
32.56	54100
32.58	52891.7
32.6	52008.3
32.62	49575
32.64	47158.3
32.66	45258.3
32.68	44075
32.7	43341.7
32.72	41991.7
32.74	41141.7
32.76	41583.3
32.78	41500
32.8	42741.7
32.82	43958.3
32.84	44250
32.86	45300
32.88	45333.3
32.9	45341.7
32.92	44700
32.94	43041.7
32.96	42075
32.98	39750
33	38500

32.3	52975
32.32	53108.3
32.34	51983.3
32.36	51466.7
32.38	51808.3
32.4	51650
32.42	52216.7
32.44	52766.7
32.46	52333.3
32.48	51483.3
32.5	51991.7
32.52	52483.3
32.54	51233.3
32.56	51358.3
32.58	51733.3
32.6	51891.7
32.62	51816.7
32.64	52033.3
32.66	51725
32.68	51533.3
32.7	52200
32.72	51900
32.74	51341.7
32.76	51275
32.78	52058.3
32.8	50891.7
32.82	51041.7
32.84	51541.7
32.86	51491.7
32.88	52316.7
32.9	51800
32.92	51333.3
32.94	50800
32.96	50516.7
32.98	51100
33	50825

32.3	29941.7
32.32	31525
32.34	32958.3
32.36	34950
32.38	36858.3
32.4	38358.3
32.42	41466.7
32.44	44150
32.46	46658.3
32.48	49116.7
32.5	51216.7
32.52	53000
32.54	54691.7
32.56	56558.3
32.58	57116.7
32.6	57383.3
32.62	56866.7
32.64	54841.7
32.66	52700
32.68	50483.3
32.7	48416.7
32.72	45758.3
32.74	43333.3
32.76	42991.7
32.78	42216.7
32.8	42525
32.82	42450
32.84	41983.3
32.86	42758.3
32.88	44691.7
32.9	45158.3
32.92	45433.3
32.94	45750
32.96	44941.7
32.98	43508.3
33	41300

32.3	24258.3
32.32	24483.3
32.34	24433.3
32.36	24966.7
32.38	25391.7
32.4	25375
32.42	25875
32.44	26516.7
32.46	27016.7
32.48	28375
32.5	29316.7
32.52	30291.7
32.54	30658.3
32.56	31833.3
32.58	33058.3
32.6	34425
32.62	36258.3
32.64	37108.3
32.66	38308.3
32.68	39608.3
32.7	41366.7
32.72	42508.3
32.74	43358.3
32.76	44358.3
32.78	45875
32.8	46441.7
32.82	46433.3
32.84	46200
32.86	45750
32.88	45358.3
32.9	44833.3
32.92	43791.7
32.94	42150
32.96	41100
32.98	40633.3
33	40508.3

33.02	36466.7
33.04	34391.7
33.06	33266.7
33.08	32316.7
33.1	32283.3
33.12	32700
33.14	33391.7
33.16	34433.3
33.18	36075
33.2	38258.3
33.22	39608.3
33.24	42083.3
33.26	45841.7
33.28	49283.3
33.3	54016.7
33.32	59250
33.34	64250
33.36	68891.7
33.38	74341.7
33.4	79575
33.42	82858.3
33.44	85616.7
33.46	85691.7
33.48	83600
33.5	79066.7
33.52	73466.7
33.54	67866.7
33.56	62700
33.58	58500
33.6	54958.3
33.62	51550
33.64	49458.3
33.66	48333.3
33.68	48225
33.7	47441.7
33.72	46541.7

33.02	50616.7
33.04	50900
33.06	50866.7
33.08	51058.3
33.1	51200
33.12	51033.3
33.14	50183.3
33.16	50208.3
33.18	50175
33.2	50700
33.22	50491.7
33.24	50708.3
33.26	50383.3
33.28	50391.7
33.3	50158.3
33.32	50450
33.34	50533.3
33.36	50358.3
33.38	50066.7
33.4	50091.7
33.42	50641.7
33.44	50300
33.46	50158.3
33.48	50308.3
33.5	50875
33.52	50525
33.54	50100
33.56	50691.7
33.58	51391.7
33.6	51366.7
33.62	51050
33.64	50258.3
33.66	49816.7
33.68	50191.7
33.7	50733.3
33.72	50391.7

33.02	39000
33.04	37150
33.06	36208.3
33.08	34691.7
33.1	33191.7
33.12	33225
33.14	33900
33.16	34183.3
33.18	34341.7
33.2	35008.3
33.22	35675
33.24	37150
33.26	38783.3
33.28	40916.7
33.3	43008.3
33.32	44516.7
33.34	47133.3
33.36	50775
33.38	55866.7
33.4	60416.7
33.42	65350
33.44	68050
33.46	70666.7
33.48	72616.7
33.5	73475
33.52	73775
33.54	72083.3
33.56	67833.3
33.58	61816.7
33.6	57516.7
33.62	55358.3
33.64	52358.3
33.66	50550
33.68	48833.3
33.7	48775
33.72	48100

33.02	38875
33.04	38241.7
33.06	38575
33.08	37941.7
33.1	37683.3
33.12	38300
33.14	38083.3
33.16	37358.3
33.18	36425
33.2	35808.3
33.22	35516.7
33.24	34741.7
33.26	34458.3
33.28	33641.7
33.3	33608.3
33.32	33391.7
33.34	32991.7
33.36	33000
33.38	31825
33.4	32441.7
33.42	32616.7
33.44	33083.3
33.46	33558.3
33.48	33408.3
33.5	34866.7
33.52	36241.7
33.54	36791.7
33.56	37633.3
33.58	39100
33.6	40616.7
33.62	41758.3
33.64	43033.3
33.66	43908.3
33.68	45125
33.7	45633.3
33.72	45716.7

33.74	45350
33.76	44483.3
33.78	44850
33.8	45216.7
33.82	45175
33.84	46441.7
33.86	48916.7
33.88	50758.3
33.9	54000
33.92	56441.7
33.94	60333.3
33.96	62750
33.98	64258.3
34	65575
34.02	66358.3
34.04	64425
34.06	62233.3
34.08	59200
34.1	56775
34.12	55200
34.14	52383.3
34.16	50733.3
34.18	49316.7
34.2	48550
34.22	47116.7
34.24	46550
34.26	45833.3
34.28	46000
34.3	46425
34.32	46066.7
34.34	46191.7
34.36	45458.3
34.38	45075
34.4	44866.7
34.42	43716.7
34.44	43725

33.74	50283.3
33.76	51016.7
33.78	50750
33.8	49666.7
33.82	49683.3
33.84	49383.3
33.86	49483.3
33.88	49658.3
33.9	49558.3
33.92	49708.3
33.94	49433.3
33.96	49600
33.98	50375
34	50400
34.02	49408.3
34.04	49200
34.06	48816.7
34.08	49075
34.1	48733.3
34.12	49100
34.14	49625
34.16	48883.3
34.18	49016.7
34.2	48966.7
34.22	48975
34.24	48591.7
34.26	48116.7
34.28	48266.7
34.3	49250
34.32	49225
34.34	49041.7
34.36	48666.7
34.38	48483.3
34.4	49133.3
34.42	48491.7
34.44	48633.3

33.74	47908.3
33.76	47416.7
33.78	46150
33.8	46383.3
33.82	46166.7
33.84	46216.7
33.86	47475
33.88	49091.7
33.9	51366.7
33.92	53566.7
33.94	56958.3
33.96	61175
33.98	65033.3
34	68425
34.02	71575
34.04	71533.3
34.06	71500
34.08	69016.7
34.1	66350
34.12	63208.3
34.14	60483.3
34.16	57791.7
34.18	55516.7
34.2	52925
34.22	49900
34.24	48875
34.26	48325
34.28	47883.3
34.3	47941.7
34.32	48175
34.34	48425
34.36	48233.3
34.38	47983.3
34.4	46800
34.42	46083.3
34.44	45325

33.74	45175
33.76	44475
33.78	43916.7
33.8	43725
33.82	42641.7
33.84	42525
33.86	42400
33.88	42108.3
33.9	41425
33.92	40358.3
33.94	39666.7
33.96	39391.7
33.98	39583.3
34	40333.3
34.02	40366.7
34.04	41375
34.06	42025
34.08	43125
34.1	44741.7
34.12	45275
34.14	46191.7
34.16	48308.3
34.18	49841.7
34.2	50958.3
34.22	51458.3
34.24	52633.3
34.26	53050
34.28	53241.7
34.3	54241.7
34.32	52775
34.34	50966.7
34.36	50150
34.38	49466.7
34.4	48183.3
34.42	46808.3
34.44	46033.3

34.46	43166.7
34.48	43475
34.5	44241.7
34.52	46075
34.54	46300
34.56	47800
34.58	48883.3
34.6	49008.3
34.62	49816.7
34.64	50450
34.66	50625
34.68	51166.7
34.7	51591.7
34.72	52783.3
34.74	53333.3
34.76	54300
34.78	54975
34.8	55008.3
34.82	53150
34.84	51908.3
34.86	49475
34.88	45391.7
34.9	41891.7
34.92	38858.3
34.94	35608.3
34.96	33141.7
34.98	30583.3
35	29366.7
35.02	28133.3
35.04	27650
35.06	28191.7
35.08	29100
35.1	29933.3
35.12	30416.7
35.14	31391.7
35.16	32400

34.46	48058.3
34.48	48400
34.5	48633.3
34.52	48733.3
34.54	48658.3
34.56	48925
34.58	48041.7
34.6	48275
34.62	48433.3
34.64	48933.3
34.66	49366.7
34.68	49141.7
34.7	49108.3
34.72	48916.7
34.74	48591.7
34.76	47808.3
34.78	47775
34.8	48483.3
34.82	48350
34.84	48166.7
34.86	47725
34.88	48250
34.9	48425
34.92	47816.7
34.94	47408.3
34.96	47441.7
34.98	47800
35	47233.3
35.02	47258.3
35.04	48075
35.06	48716.7
35.08	47416.7
35.1	47291.7
35.12	47825
35.14	47391.7
35.16	47333.3

34.46	43741.7
34.48	43233.3
34.5	43500
34.52	43175
34.54	43150
34.56	45033.3
34.58	45800
34.6	46208.3
34.62	46850
34.64	48516.7
34.66	48425
34.68	48000
34.7	49358.3
34.72	50150
34.74	50325
34.76	51866.7
34.78	52958.3
34.8	53633.3
34.82	54566.7
34.84	55275
34.86	53841.7
34.88	51091.7
34.9	48958.3
34.92	45108.3
34.94	40408.3
34.96	36966.7
34.98	34850
35	32375
35.02	29991.7
35.04	29108.3
35.06	27916.7
35.08	27716.7
35.1	28425
35.12	27883.3
35.14	28475
35.16	29550

34.46	45050
34.48	44650
34.5	43916.7
34.52	42900
34.54	42425
34.56	41366.7
34.58	41816.7
34.6	41508.3
34.62	40750
34.64	41000
34.66	40208.3
34.68	40225
34.7	39308.3
34.72	38491.7
34.74	38933.3
34.76	39458.3
34.78	39250
34.8	38875
34.82	39633.3
34.84	39383.3
34.86	39575
34.88	39858.3
34.9	40491.7
34.92	40933.3
34.94	41625
34.96	40800
34.98	40975
35	41166.7
35.02	41341.7
35.04	41133.3
35.06	40508.3
35.08	40191.7
35.1	39316.7
35.12	38591.7
35.14	37900
35.16	36608.3

35.18	34175
35.2	35741.7
35.22	36700
35.24	38141.7
35.26	39066.7
35.28	40333.3
35.3	40675
35.32	40333.3
35.34	39600
35.36	38133.3
35.38	36541.7
35.4	35441.7
35.42	33950
35.44	33408.3
35.46	32758.3
35.48	33016.7
35.5	33416.7
35.52	34016.7
35.54	35175
35.56	34658.3
35.58	35600
35.6	35825
35.62	35600
35.64	34991.7
35.66	33866.7
35.68	32383.3
35.7	30566.7
35.72	28158.3
35.74	25891.7
35.76	24833.3
35.78	23541.7
35.8	22383.3
35.82	22225
35.84	21650
35.86	21225
35.88	21233.3

35.18	47341.7
35.2	47575
35.22	47650
35.24	47541.7
35.26	47391.7
35.28	46400
35.3	47433.3
35.32	48158.3
35.34	47891.7
35.36	47966.7
35.38	47066.7
35.4	47416.7
35.42	47558.3
35.44	47850
35.46	47383.3
35.48	47075
35.5	46800
35.52	46883.3
35.54	47716.7
35.56	47366.7
35.58	47525
35.6	46925
35.62	46708.3
35.64	47433.3
35.66	47508.3
35.68	47158.3
35.7	46550
35.72	47258.3
35.74	46516.7
35.76	47125
35.78	47200
35.8	46700
35.82	47241.7
35.84	47500
35.86	47016.7
35.88	46183.3

35.18	30475
35.2	31550
35.22	31800
35.24	34133.3
35.26	35541.7
35.28	36050
35.3	37500
35.32	39091.7
35.34	39975
35.36	40050
35.38	38333.3
35.4	36425
35.42	35058.3
35.44	34600
35.46	33383.3
35.48	32475
35.5	31525
35.52	31225
35.54	31675
35.56	31500
35.58	32950
35.6	33716.7
35.62	33933.3
35.64	34400
35.66	33675
35.68	33658.3
35.7	32400
35.72	30533.3
35.74	28925
35.76	27183.3
35.78	26025
35.8	24666.7
35.82	23241.7
35.84	22208.3
35.86	22475
35.88	22283.3

35.18	35900
35.2	34241.7
35.22	33008.3
35.24	32383.3
35.26	31158.3
35.28	30908.3
35.3	30475
35.32	30108.3
35.34	30083.3
35.36	29958.3
35.38	29275
35.4	29000
35.42	29241.7
35.44	29541.7
35.46	29800
35.48	30241.7
35.5	30508.3
35.52	29933.3
35.54	29866.7
35.56	29825
35.58	29516.7
35.6	29866.7
35.62	29200
35.64	29008.3
35.66	28650
35.68	28183.3
35.7	28208.3
35.72	28508.3
35.74	27716.7
35.76	27500
35.78	27416.7
35.8	27375
35.82	27475
35.84	27700
35.86	28091.7
35.88	27833.3

35.9	21525
35.92	21716.7
35.94	21325
35.96	21933.3
35.98	22116.7
36	22450
36.02	22425
36.04	23183.3
36.06	23716.7
36.08	23791.7
36.1	23600
36.12	24575
36.14	24808.3
36.16	24866.7
36.18	24866.7
36.2	24341.7
36.22	23708.3
36.24	23000
36.26	22658.3
36.28	22708.3
36.3	22625
36.32	22591.7
36.34	22841.7
36.36	22558.3
36.38	22841.7
36.4	23358.3
36.42	24158.3
36.44	25075
36.46	25666.7
36.48	26550
36.5	27683.3
36.52	28016.7
36.54	28191.7
36.56	28800
36.58	29850
36.6	29041.7

35.9	46608.3
35.92	46408.3
35.94	45825
35.96	46625
35.98	46283.3
36	46716.7
36.02	46991.7
36.04	46091.7
36.06	45933.3
36.08	45816.7
36.1	46616.7
36.12	47066.7
36.14	47125
36.16	45491.7
36.18	46150
36.2	46050
36.22	46433.3
36.24	45950
36.26	45383.3
36.28	45708.3
36.3	45850
36.32	45641.7
36.34	45233.3
36.36	46133.3
36.38	45816.7
36.4	45350
36.42	45850
36.44	45916.7
36.46	46058.3
36.48	46216.7
36.5	45675
36.52	45216.7
36.54	45608.3
36.56	45316.7
36.58	45333.3
36.6	45758.3

35.9	21350
35.92	21533.3
35.94	21683.3
35.96	22166.7
35.98	22116.7
36	21733.3
36.02	22208.3
36.04	22550
36.06	22741.7
36.08	23108.3
36.1	23475
36.12	24108.3
36.14	24525
36.16	24841.7
36.18	24891.7
36.2	24891.7
36.22	25533.3
36.24	25125
36.26	24641.7
36.28	24250
36.3	24383.3
36.32	23041.7
36.34	23333.3
36.36	23325
36.38	23025
36.4	23850
36.42	24241.7
36.44	24950
36.46	24708.3
36.48	25475
36.5	26625
36.52	26941.7
36.54	28291.7
36.56	29091.7
36.58	29041.7
36.6	29300

35.9	26791.7
35.92	26158.3
35.94	26000
35.96	25725
35.98	25491.7
36	24616.7
36.02	24425
36.04	23750
36.06	22683.3
36.08	22125
36.1	22275
36.12	22308.3
36.14	21841.7
36.16	21458.3
36.18	21583.3
36.2	21791.7
36.22	21533.3
36.24	22083.3
36.26	22008.3
36.28	22216.7
36.3	21725
36.32	22075
36.34	22266.7
36.36	22933.3
36.38	23141.7
36.4	22400
36.42	22491.7
36.44	22750
36.46	22700
36.48	22691.7
36.5	23041.7
36.52	23183.3
36.54	22983.3
36.56	22958.3
36.58	22675
36.6	22975

36.62	28900
36.64	29116.7
36.66	27708.3
36.68	26041.7
36.7	25558.3
36.72	24850
36.74	23708.3
36.76	23158.3
36.78	23025
36.8	23000
36.82	23116.7
36.84	23100
36.86	22975
36.88	23491.7
36.9	23908.3
36.92	24300
36.94	25766.7
36.96	25708.3
36.98	27150
37	28975
37.02	30175
37.04	32591.7
37.06	35091.7
37.08	37933.3
37.1	40575
37.12	43858.3
37.14	47816.7
37.16	52191.7
37.18	56708.3
37.2	60658.3
37.22	65341.7
37.24	69308.3
37.26	73041.7
37.28	76200
37.3	78175
37.32	80391.7

36.62	45391.7
36.64	45300
36.66	45925
36.68	45708.3
36.7	44858.3
36.72	45591.7
36.74	45208.3
36.76	44283.3
36.78	45058.3
36.8	46333.3
36.82	45258.3
36.84	45341.7
36.86	44691.7
36.88	44616.7
36.9	44683.3
36.92	44966.7
36.94	45416.7
36.96	44633.3
36.98	44566.7
37	44716.7
37.02	45108.3
37.04	44266.7
37.06	44208.3
37.08	44900
37.1	44975
37.12	44000
37.14	43650
37.16	43925
37.18	44541.7
37.2	44408.3
37.22	43925
37.24	43941.7
37.26	44083.3
37.28	43958.3
37.3	44408.3
37.32	44033.3

36.62	29825
36.64	30283.3
36.66	29108.3
36.68	28741.7
36.7	27583.3
36.72	26150
36.74	24625
36.76	24783.3
36.78	24141.7
36.8	23308.3
36.82	23416.7
36.84	24158.3
36.86	23966.7
36.88	24041.7
36.9	23750
36.92	24825
36.94	25141.7
36.96	25900
36.98	27441.7
37	28508.3
37.02	29533.3
37.04	31366.7
37.06	32641.7
37.08	35150
37.1	37608.3
37.12	40291.7
37.14	43675
37.16	47300
37.18	51291.7
37.2	55466.7
37.22	60491.7
37.24	63158.3
37.26	66933.3
37.28	70483.3
37.3	75475
37.32	79166.7

36.62	23358.3
36.64	23308.3
36.66	23375
36.68	23458.3
36.7	23775
36.72	24425
36.74	24883.3
36.76	24908.3
36.78	24908.3
36.8	25591.7
36.82	25908.3
36.84	25141.7
36.86	25708.3
36.88	25575
36.9	25575
36.92	24733.3
36.94	24341.7
36.96	24733.3
36.98	24341.7
37	23750
37.02	23583.3
37.04	23991.7
37.06	23133.3
37.08	23633.3
37.1	24283.3
37.12	24175
37.14	24583.3
37.16	25141.7
37.18	26241.7
37.2	26891.7
37.22	27033.3
37.24	28675
37.26	28950
37.28	31025
37.3	32925
37.32	34975

37.34	81225
37.36	79983.3
37.38	77875
37.4	74616.7
37.42	70258.3
37.44	64466.7
37.46	60216.7
37.48	54325
37.5	49491.7
37.52	45866.7
37.54	41766.7
37.56	38908.3
37.58	36108.3
37.6	34291.7
37.62	32608.3
37.64	30250
37.66	28958.3
37.68	28225
37.7	27425
37.72	26433.3
37.74	26550
37.76	26091.7
37.78	26200
37.8	25950
37.82	25308.3
37.84	25241.7
37.86	25250
37.88	25008.3
37.9	24091.7
37.92	23625
37.94	22633.3
37.96	21508.3
37.98	21491.7
38	20691.7
38.02	20450
38.04	19850

37.34	44858.3
37.36	44491.7
37.38	44008.3
37.4	43758.3
37.42	43558.3
37.44	43925
37.46	42866.7
37.48	43133.3
37.5	44250
37.52	43608.3
37.54	44658.3
37.56	43750
37.58	43791.7
37.6	43316.7
37.62	42983.3
37.64	43133.3
37.66	43775
37.68	43183.3
37.7	43066.7
37.72	42900
37.74	43216.7
37.76	43266.7
37.78	43491.7
37.8	42900
37.82	43975
37.84	43150
37.86	43758.3
37.88	43283.3
37.9	44083.3
37.92	43625
37.94	43508.3
37.96	44075
37.98	43933.3
38	43200
38.02	42341.7
38.04	42958.3

37.34	81083.3
37.36	83850
37.38	84941.7
37.4	84950
37.42	83383.3
37.44	79208.3
37.46	73700
37.48	67958.3
37.5	60891.7
37.52	55983.3
37.54	51941.7
37.56	47283.3
37.58	43041.7
37.6	39308.3
37.62	36141.7
37.64	33083.3
37.66	31150
37.68	29733.3
37.7	28833.3
37.72	28250
37.74	27033.3
37.76	26900
37.78	26475
37.8	26408.3
37.82	26308.3
37.84	25916.7
37.86	26700
37.88	26050
37.9	25891.7
37.92	25375
37.94	24650
37.96	23750
37.98	23166.7
38	22225
38.02	21466.7
38.04	21283.3

37.34	36808.3
37.36	37916.7
37.38	40500
37.4	43725
37.42	45841.7
37.44	46883.3
37.46	49891.7
37.48	52591.7
37.5	54291.7
37.52	55716.7
37.54	57850
37.56	58441.7
37.58	58858.3
37.6	58891.7
37.62	59450
37.64	58266.7
37.66	56466.7
37.68	54675
37.7	53333.3
37.72	50058.3
37.74	46633.3
37.76	44483.3
37.78	41133.3
37.8	39116.7
37.82	36858.3
37.84	34758.3
37.86	32166.7
37.88	30658.3
37.9	28875
37.92	28216.7
37.94	27375
37.96	26500
37.98	25925
38	25458.3
38.02	24708.3
38.04	24183.3

38.06	20041.7
38.08	19841.7
38.1	20491.7
38.12	20700
38.14	19933.3
38.16	20566.7
38.18	21200
38.2	20891.7
38.22	21408.3
38.24	21566.7
38.26	22175
38.28	23033.3
38.3	23833.3
38.32	24150
38.34	24691.7
38.36	25400
38.38	26250
38.4	25766.7
38.42	26250
38.44	26383.3
38.46	25925
38.48	25566.7
38.5	25841.7
38.52	25458.3
38.54	25250
38.56	25558.3
38.58	25300
38.6	24441.7
38.62	25475
38.64	25016.7
38.66	24825
38.68	25183.3
38.7	26283.3
38.72	27358.3
38.74	27566.7
38.76	28941.7

38.06	42691.7
38.08	42675
38.1	42541.7
38.12	42933.3
38.14	42791.7
38.16	42991.7
38.18	43633.3
38.2	43391.7
38.22	43266.7
38.24	42875
38.26	42850
38.28	42525
38.3	43383.3
38.32	42425
38.34	42591.7
38.36	43025
38.38	42741.7
38.4	42700
38.42	42466.7
38.44	42583.3
38.46	42116.7
38.48	42333.3
38.5	42233.3
38.52	42658.3
38.54	42391.7
38.56	41558.3
38.58	42041.7
38.6	42016.7
38.62	43100
38.64	42375
38.66	41725
38.68	42050
38.7	42050
38.72	41491.7
38.74	41500
38.76	41066.7

38.06	20741.7
38.08	20508.3
38.1	20375
38.12	20641.7
38.14	20850
38.16	20758.3
38.18	21350
38.2	21791.7
38.22	22416.7
38.24	22875
38.26	23183.3
38.28	23766.7
38.3	23841.7
38.32	24850
38.34	24775
38.36	25341.7
38.38	26066.7
38.4	26883.3
38.42	27425
38.44	27808.3
38.46	27825
38.48	27408.3
38.5	27083.3
38.52	27091.7
38.54	27166.7
38.56	27316.7
38.58	27016.7
38.6	26191.7
38.62	25891.7
38.64	25808.3
38.66	25691.7
38.68	26491.7
38.7	26166.7
38.72	27216.7
38.74	28058.3
38.76	28950

38.06	24566.7
38.08	23858.3
38.1	23575
38.12	23000
38.14	22866.7
38.16	23275
38.18	22491.7
38.2	22225
38.22	21408.3
38.24	21533.3
38.26	21366.7
38.28	20783.3
38.3	20900
38.32	21141.7
38.34	21133.3
38.36	21175
38.38	21175
38.4	21141.7
38.42	21591.7
38.44	21641.7
38.46	22225
38.48	22791.7
38.5	23033.3
38.52	23150
38.54	23891.7
38.56	23666.7
38.58	24000
38.6	24508.3
38.62	24150
38.64	25241.7
38.66	24641.7
38.68	24383.3
38.7	24691.7
38.72	24483.3
38.74	24408.3
38.76	24566.7

38.78	29841.7
38.8	31025
38.82	31058.3
38.84	31675
38.86	32291.7
38.88	32891.7
38.9	33583.3
38.92	34241.7
38.94	34275
38.96	34166.7
38.98	33766.7
39	33900
39.02	33008.3
39.04	32241.7
39.06	30608.3
39.08	29341.7
39.1	28741.7
39.12	29683.3
39.14	30341.7
39.16	29783.3
39.18	30550
39.2	30500
39.22	30258.3
39.24	30375
39.26	31091.7
39.28	32791.7
39.3	34133.3
39.32	35258.3
39.34	36725
39.36	37208.3
39.38	39116.7
39.4	40458.3
39.42	41616.7
39.44	43483.3
39.46	43650
39.48	44116.7

38.78	41408.3
38.8	41625
38.82	41466.7
38.84	42591.7
38.86	41900
38.88	42025
38.9	41866.7
38.92	41191.7
38.94	41133.3
38.96	42341.7
38.98	41233.3
39	41116.7
39.02	42075
39.04	41850
39.06	41125
39.08	42325
39.1	41833.3
39.12	41200
39.14	40766.7
39.16	41683.3
39.18	41733.3
39.2	41900
39.22	42033.3
39.24	41600
39.26	40925
39.28	41008.3
39.3	41225
39.32	40633.3
39.34	41800
39.36	41425
39.38	40950
39.4	41225
39.42	40883.3
39.44	40700
39.46	41233.3
39.48	41808.3

38.78	29991.7
38.8	30625
38.82	31366.7
38.84	31958.3
38.86	33208.3
38.88	34600
38.9	34625
38.92	35600
38.94	35666.7
38.96	35650
38.98	35550
39	35158.3
39.02	34775
39.04	34166.7
39.06	32691.7
39.08	31308.3
39.1	30616.7
39.12	30341.7
39.14	30783.3
39.16	31133.3
39.18	31008.3
39.2	30633.3
39.22	30316.7
39.24	30925
39.26	31583.3
39.28	31225
39.3	31891.7
39.32	31966.7
39.34	32891.7
39.36	33233.3
39.38	33525
39.4	35125
39.42	36450
39.44	38216.7
39.46	38825
39.48	39766.7

38.78	23900
38.8	24491.7
38.82	24858.3
38.84	24400
38.86	24491.7
38.88	25100
38.9	25333.3
38.92	25333.3
38.94	26100
38.96	25841.7
38.98	26700
39	27650
39.02	27916.7
39.04	28575
39.06	28475
39.08	28725
39.1	29658.3
39.12	29075
39.14	29875
39.16	29533.3
39.18	30083.3
39.2	29800
39.22	29275
39.24	29766.7
39.26	28533.3
39.28	28683.3
39.3	28233.3
39.32	28958.3
39.34	27991.7
39.36	27675
39.38	27275
39.4	26991.7
39.42	26625
39.44	26591.7
39.46	27075
39.48	27166.7

39.5	43308.3
39.52	41033.3
39.54	38816.7
39.56	37550
39.58	35216.7
39.6	32925
39.62	31208.3
39.64	29891.7
39.66	29083.3
39.68	28683.3
39.7	29375
39.72	29091.7
39.74	30458.3
39.76	31483.3
39.78	32750
39.8	33858.3
39.82	35550
39.84	36933.3
39.86	38191.7
39.88	39658.3
39.9	39708.3
39.92	41108.3
39.94	41300
39.96	43283.3
39.98	44000
40	43425
40.02	42216.7
40.04	40733.3
40.06	40233.3
40.08	37775
40.1	35575
40.12	33575
40.14	30533.3
40.16	28883.3
40.18	26175
40.2	24883.3

39.5	40750
39.52	41291.7
39.54	41191.7
39.56	41950
39.58	41058.3
39.6	41675
39.62	40383.3
39.64	40625
39.66	41200
39.68	40741.7
39.7	39900
39.72	40016.7
39.74	40633.3
39.76	40891.7
39.78	41433.3
39.8	40608.3
39.82	40733.3
39.84	41141.7
39.86	40366.7
39.88	40350
39.9	40841.7
39.92	41141.7
39.94	41283.3
39.96	40658.3
39.98	40391.7
40	40041.7
40.02	40100
40.04	39316.7
40.06	39933.3
40.08	40141.7
40.1	40675
40.12	39775
40.14	39633.3
40.16	39841.7
40.18	39733.3
40.2	40108.3

39.5	39425
39.52	39658.3
39.54	38158.3
39.56	37541.7
39.58	36650
39.6	33683.3
39.62	32066.7
39.64	31158.3
39.66	30325
39.68	29733.3
39.7	29466.7
39.72	30908.3
39.74	30350
39.76	30716.7
39.78	31425
39.8	33141.7
39.82	34241.7
39.84	35808.3
39.86	36683.3
39.88	38083.3
39.9	38958.3
39.92	39558.3
39.94	40691.7
39.96	40000
39.98	40858.3
40	41066.7
40.02	42025
40.04	42291.7
40.06	41700
40.08	39416.7
40.1	38566.7
40.12	36566.7
40.14	33850
40.16	32508.3
40.18	30683.3
40.2	28600

39.5	27125
39.52	27766.7
39.54	27441.7
39.56	27516.7
39.58	28058.3
39.6	27316.7
39.62	28108.3
39.64	28975
39.66	28775
39.68	28966.7
39.7	28091.7
39.72	28366.7
39.74	28050
39.76	27475
39.78	27700
39.8	27741.7
39.82	27758.3
39.84	27225
39.86	27100
39.88	26725
39.9	26616.7
39.92	26275
39.94	26500
39.96	27716.7
39.98	28333.3
40	28550
40.02	28883.3
40.04	29741.7
40.06	30900
40.08	31575
40.1	31850
40.12	32266.7
40.14	33150
40.16	32525
40.18	31850
40.2	32133.3

40.22	23525
40.24	22158.3
40.26	21433.3
40.28	20300
40.3	19775
40.32	19741.7
40.34	19366.7
40.36	19191.7
40.38	18891.7
40.4	18766.7
40.42	19191.7
40.44	19091.7
40.46	19316.7
40.48	19375
40.5	19900
40.52	19783.3
40.54	19800
40.56	19741.7
40.58	20900
40.6	21475
40.62	22350
40.64	22133.3
40.66	22591.7
40.68	23316.7
40.7	24008.3
40.72	24541.7
40.74	24925
40.76	25141.7
40.78	25833.3
40.8	25191.7
40.82	25233.3
40.84	25558.3
40.86	24275
40.88	24000
40.9	23900
40.92	23616.7

40.22	40391.7
40.24	40266.7
40.26	41016.7
40.28	40483.3
40.3	40175
40.32	40250
40.34	40058.3
40.36	39550
40.38	41175
40.4	39825
40.42	39875
40.44	40008.3
40.46	40733.3
40.48	40058.3
40.5	40300
40.52	39791.7
40.54	38775
40.56	39500
40.58	39700
40.6	39725
40.62	39816.7
40.64	39916.7
40.66	40525
40.68	40383.3
40.7	39566.7
40.72	39333.3
40.74	39716.7
40.76	39075
40.78	39675
40.8	40216.7
40.82	39875
40.84	39675
40.86	39341.7
40.88	38891.7
40.9	38658.3
40.92	39616.7

40.22	27125
40.24	25158.3
40.26	23666.7
40.28	22033.3
40.3	21625
40.32	20708.3
40.34	20091.7
40.36	20116.7
40.38	19666.7
40.4	19958.3
40.42	18658.3
40.44	19775
40.46	19366.7
40.48	19883.3
40.5	19741.7
40.52	20508.3
40.54	20583.3
40.56	20216.7
40.58	20233.3
40.6	20350
40.62	21358.3
40.64	20725
40.66	21091.7
40.68	21958.3
40.7	23116.7
40.72	22691.7
40.74	23233.3
40.76	23533.3
40.78	23808.3
40.8	23933.3
40.82	25150
40.84	25075
40.86	24208.3
40.88	24041.7
40.9	24191.7
40.92	23633.3

40.22	32233.3
40.24	31816.7
40.26	31025
40.28	31008.3
40.3	30300
40.32	29941.7
40.34	29150
40.36	28208.3
40.38	27341.7
40.4	26216.7
40.42	25175
40.44	23866.7
40.46	22641.7
40.48	22541.7
40.5	22050
40.52	21125
40.54	20550
40.56	21016.7
40.58	20483.3
40.6	20116.7
40.62	19575
40.64	19350
40.66	19691.7
40.68	19966.7
40.7	19608.3
40.72	19558.3
40.74	19733.3
40.76	19641.7
40.78	19941.7
40.8	19833.3
40.82	19333.3
40.84	19841.7
40.86	20125
40.88	19783.3
40.9	20475
40.92	20483.3

40.94	23766.7
40.96	23391.7
40.98	23216.7
41	23033.3
41.02	22341.7
41.04	22683.3
41.06	22775
41.08	23225
41.1	23000
41.12	23966.7
41.14	23266.7
41.16	24241.7
41.18	24150
41.2	24191.7
41.22	24308.3
41.24	24366.7
41.26	25150
41.28	25475
41.3	26300
41.32	26541.7
41.34	28050
41.36	28783.3
41.38	29658.3
41.4	29858.3
41.42	31400
41.44	32691.7
41.46	33100
41.48	34508.3
41.5	34158.3
41.52	33725
41.54	32366.7
41.56	31958.3
41.58	31116.7
41.6	29950
41.62	27850
41.64	26391.7

40.94	38858.3
40.96	39075
40.98	38966.7
41	38875
41.02	38858.3
41.04	38825
41.06	38475
41.08	39850
41.1	39425
41.12	38725
41.14	38775
41.16	38975
41.18	39358.3
41.2	38150
41.22	39225
41.24	39608.3
41.26	39483.3
41.28	37991.7
41.3	38391.7
41.32	39050
41.34	38908.3
41.36	38475
41.38	38491.7
41.4	38475
41.42	39650
41.44	39525
41.46	38391.7
41.48	39091.7
41.5	38675
41.52	38650
41.54	38675
41.56	38816.7
41.58	39050
41.6	38033.3
41.62	39316.7
41.64	38091.7

40.94	22950
40.96	23066.7
40.98	22700
41	22916.7
41.02	23125
41.04	23425
41.06	22558.3
41.08	23233.3
41.1	23366.7
41.12	23975
41.14	23900
41.16	24566.7
41.18	24766.7
41.2	24933.3
41.22	24900
41.24	24500
41.26	24975
41.28	25400
41.3	26150
41.32	26541.7
41.34	27041.7
41.36	28216.7
41.38	28416.7
41.4	29608.3
41.42	30816.7
41.44	31433.3
41.46	31858.3
41.48	32183.3
41.5	33250
41.52	34250
41.54	33325
41.56	34033.3
41.58	33450
41.6	32533.3
41.62	30241.7
41.64	29058.3

40.94	20533.3
40.96	21125
40.98	21575
41	20508.3
41.02	21525
41.04	20650
41.06	21066.7
41.08	21183.3
41.1	21141.7
41.12	21516.7
41.14	21450
41.16	21033.3
41.18	21166.7
41.2	20733.3
41.22	20591.7
41.24	21516.7
41.26	20941.7
41.28	20825
41.3	21016.7
41.32	22383.3
41.34	22050
41.36	21683.3
41.38	22083.3
41.4	22416.7
41.42	23283.3
41.44	23108.3
41.46	22916.7
41.48	24091.7
41.5	23850
41.52	24508.3
41.54	24958.3
41.56	24683.3
41.58	25016.7
41.6	25558.3
41.62	26058.3
41.64	26991.7

41.66	25458.3
41.68	24600
41.7	23300
41.72	22466.7
41.74	21816.7
41.76	21366.7
41.78	21958.3
41.8	20550
41.82	21300
41.84	22633.3
41.86	22175
41.88	22300
41.9	22450
41.92	23225
41.94	23166.7
41.96	23958.3
41.98	24275
42	24825
42.02	24600
42.04	24500
42.06	24775
42.08	25091.7
42.1	25025
42.12	24816.7
42.14	24966.7
42.16	25075
42.18	24633.3
42.2	24291.7
42.22	23933.3
42.24	24133.3
42.26	24925
42.28	24816.7
42.3	25341.7
42.32	26466.7
42.34	26525
42.36	25950

41.66	38700
41.68	38466.7
41.7	39425
41.72	38141.7
41.74	38000
41.76	38775
41.78	39041.7
41.8	38425
41.82	38025
41.84	38416.7
41.86	38083.3
41.88	37533.3
41.9	38566.7
41.92	39400
41.94	38625
41.96	37725
41.98	37550
42	37908.3
42.02	37816.7
42.04	39058.3
42.06	37900
42.08	38050
42.1	37941.7
42.12	37833.3
42.14	38366.7
42.16	38741.7
42.18	37500
42.2	37508.3
42.22	37150
42.24	38391.7
42.26	38650
42.28	39041.7
42.3	38391.7
42.32	38575
42.34	37791.7
42.36	38125

41.66	28016.7
41.68	26208.3
41.7	25425
41.72	24108.3
41.74	23608.3
41.76	23258.3
41.78	22933.3
41.8	22283.3
41.82	21908.3
41.84	21700
41.86	21666.7
41.88	22791.7
41.9	22816.7
41.92	22591.7
41.94	23791.7
41.96	23775
41.98	24875
42	24666.7
42.02	25600
42.04	25175
42.06	25950
42.08	25583.3
42.1	26308.3
42.12	25891.7
42.14	25816.7
42.16	26550
42.18	25825
42.2	25958.3
42.22	25808.3
42.24	25066.7
42.26	25275
42.28	25750
42.3	25525
42.32	25733.3
42.34	26225
42.36	26775

41.66	26041.7
41.68	26616.7
41.7	26800
41.72	26441.7
41.74	26833.3
41.76	26566.7
41.78	26758.3
41.8	27083.3
41.82	26625
41.84	25191.7
41.86	25075
41.88	24725
41.9	24341.7
41.92	23341.7
41.94	23000
41.96	21958.3
41.98	22891.7
42	22591.7
42.02	21791.7
42.04	21658.3
42.06	22475
42.08	22308.3
42.1	21991.7
42.12	21425
42.14	20691.7
42.16	21175
42.18	22033.3
42.2	22516.7
42.22	22925
42.24	23058.3
42.26	23425
42.28	24025
42.3	23266.7
42.32	22325
42.34	22783.3
42.36	23541.7

42.38	25683.3
42.4	25433.3
42.42	26333.3
42.44	26175
42.46	25758.3
42.48	26333.3
42.5	26200
42.52	27366.7
42.54	27366.7
42.56	28008.3
42.58	28675
42.6	30008.3
42.62	30825
42.64	31108.3
42.66	32658.3
42.68	33058.3
42.7	33475
42.72	34425
42.74	35133.3
42.76	35525
42.78	35533.3
42.8	35000
42.82	34583.3
42.84	34025
42.86	33700
42.88	33666.7
42.9	34033.3
42.92	34125
42.94	34483.3
42.96	35533.3
42.98	35275
43	35825
43.02	36316.7
43.04	36241.7
43.06	36625
43.08	36583.3

42.38	39016.7
42.4	38300
42.42	38158.3
42.44	38341.7
42.46	38233.3
42.48	37633.3
42.5	38408.3
42.52	37625
42.54	37466.7
42.56	37191.7
42.58	37625
42.6	38075
42.62	37641.7
42.64	36975
42.66	37558.3
42.68	37308.3
42.7	37216.7
42.72	36525
42.74	37558.3
42.76	37200
42.78	36258.3
42.8	36983.3
42.82	36850
42.84	37091.7
42.86	37550
42.88	37241.7
42.9	38050
42.92	37258.3
42.94	37333.3
42.96	36508.3
42.98	37025
43	35675
43.02	36325
43.04	36766.7
43.06	36950
43.08	36800

42.38	25991.7
42.4	27016.7
42.42	26766.7
42.44	26375
42.46	27250
42.48	26508.3
42.5	26808.3
42.52	27391.7
42.54	27641.7
42.56	27833.3
42.58	28608.3
42.6	29300
42.62	30050
42.64	30208.3
42.66	31641.7
42.68	33216.7
42.7	35116.7
42.72	35316.7
42.74	36758.3
42.76	37158.3
42.78	38275
42.8	38775
42.82	38425
42.84	37858.3
42.86	37208.3
42.88	36700
42.9	35783.3
42.92	34908.3
42.94	35566.7
42.96	35933.3
42.98	36741.7
43	36108.3
43.02	36591.7
43.04	36816.7
43.06	37308.3
43.08	37808.3

42.38	22883.3
42.4	23658.3
42.42	23208.3
42.44	23183.3
42.46	23483.3
42.48	23316.7
42.5	23600
42.52	23241.7
42.54	23391.7
42.56	23500
42.58	23158.3
42.6	23575
42.62	24150
42.64	23966.7
42.66	24666.7
42.68	24150
42.7	24466.7
42.72	25575
42.74	25391.7
42.76	25900
42.78	26466.7
42.8	25725
42.82	27575
42.84	27541.7
42.86	28858.3
42.88	29066.7
42.9	28975
42.92	30008.3
42.94	30525
42.96	30633.3
42.98	31508.3
43	32125
43.02	32416.7
43.04	32291.7
43.06	32591.7
43.08	32091.7

43.1	36966.7
43.12	36075
43.14	35825
43.16	35483.3
43.18	34991.7
43.2	34658.3
43.22	34258.3
43.24	33316.7
43.26	31800
43.28	31275
43.3	30125
43.32	28966.7
43.34	28208.3
43.36	27125
43.38	26375
43.4	27166.7
43.42	26700
43.44	25516.7
43.46	26950
43.48	26775
43.5	26908.3
43.52	28075
43.54	28441.7
43.56	30300
43.58	31766.7
43.6	33025
43.62	33975
43.64	33650
43.66	34158.3
43.68	34208.3
43.7	34058.3
43.72	32725
43.74	32941.7
43.76	31500
43.78	31783.3
43.8	30383.3

43.1	36283.3
43.12	36200
43.14	37075
43.16	37208.3
43.18	37616.7
43.2	36858.3
43.22	37050
43.24	37608.3
43.26	36908.3
43.28	36608.3
43.3	36833.3
43.32	36691.7
43.34	36266.7
43.36	35733.3
43.38	35566.7
43.4	36000
43.42	36841.7
43.44	35866.7
43.46	36366.7
43.48	35900
43.5	36033.3
43.52	36466.7
43.54	35725
43.56	36908.3
43.58	35933.3
43.6	36333.3
43.62	36450
43.64	36500
43.66	36291.7
43.68	35441.7
43.7	35500
43.72	36008.3
43.74	36000
43.76	35691.7
43.78	35641.7
43.8	36083.3

43.1	37383.3
43.12	37341.7
43.14	36558.3
43.16	38250
43.18	37350
43.2	37375
43.22	37391.7
43.24	37083.3
43.26	36250
43.28	35358.3
43.3	34458.3
43.32	32700
43.34	30375
43.36	29808.3
43.38	29550
43.4	28933.3
43.42	27925
43.44	27433.3
43.46	27166.7
43.48	26675
43.5	26650
43.52	27300
43.54	27466.7
43.56	29325
43.58	30250
43.6	31483.3
43.62	31600
43.64	33691.7
43.66	33875
43.68	35300
43.7	35408.3
43.72	35533.3
43.74	33666.7
43.76	33616.7
43.78	31691.7
43.8	31558.3

43.1	31175
43.12	30383.3
43.14	30941.7
43.16	31341.7
43.18	31525
43.2	30716.7
43.22	31175
43.24	31150
43.26	31066.7
43.28	30616.7
43.3	30308.3
43.32	30275
43.34	30791.7
43.36	30575
43.38	30725
43.4	31108.3
43.42	31125
43.44	29975
43.46	31091.7
43.48	30408.3
43.5	29966.7
43.52	30358.3
43.54	30033.3
43.56	28650
43.58	27783.3
43.6	28175
43.62	26558.3
43.64	26033.3
43.66	25866.7
43.68	25183.3
43.7	24741.7
43.72	25125
43.74	25008.3
43.76	24683.3
43.78	25491.7
43.8	24650

43.82	29566.7
43.84	28391.7
43.86	27466.7
43.88	25958.3
43.9	24916.7
43.92	24866.7
43.94	23766.7
43.96	22833.3
43.98	22050
44	20800
44.02	21016.7
44.04	20800
44.06	20275
44.08	18583.3
44.1	18900
44.12	18808.3
44.14	18333.3
44.16	17783.3
44.18	18266.7
44.2	17791.7
44.22	18708.3
44.24	18333.3
44.26	18158.3
44.28	18483.3
44.3	18400
44.32	17991.7
44.34	18508.3
44.36	18400
44.38	18558.3
44.4	18550
44.42	19050
44.44	18741.7
44.46	18666.7
44.48	18491.7
44.5	18966.7
44.52	18966.7

43.82	35925
43.84	35633.3
43.86	35616.7
43.88	34908.3
43.9	35958.3
43.92	35708.3
43.94	35541.7
43.96	35225
43.98	35491.7
44	35950
44.02	35750
44.04	35425
44.06	35816.7
44.08	35225
44.1	35125
44.12	34966.7
44.14	35450
44.16	34808.3
44.18	35150
44.2	34283.3
44.22	35150
44.24	35241.7
44.26	34775
44.28	35775
44.3	34708.3
44.32	34591.7
44.34	34141.7
44.36	34441.7
44.38	34783.3
44.4	34975
44.42	34750
44.44	34491.7
44.46	34241.7
44.48	34200
44.5	33891.7
44.52	35183.3

43.82	30416.7
43.84	29566.7
43.86	28466.7
43.88	28400
43.9	27333.3
43.92	26341.7
43.94	25508.3
43.96	25066.7
43.98	23100
44	22716.7
44.02	21341.7
44.04	22125
44.06	21433.3
44.08	20866.7
44.1	20658.3
44.12	20083.3
44.14	19175
44.16	19041.7
44.18	19725
44.2	18691.7
44.22	18941.7
44.24	19450
44.26	19350
44.28	19041.7
44.3	18591.7
44.32	18725
44.34	18275
44.36	18125
44.38	18183.3
44.4	19116.7
44.42	19500
44.44	19033.3
44.46	18991.7
44.48	19516.7
44.5	19750
44.52	19850

43.82	25250
43.84	26266.7
43.86	26258.3
43.88	26466.7
43.9	26816.7
43.92	26366.7
43.94	27100
43.96	26691.7
43.98	26850
44	26533.3
44.02	25133.3
44.04	25400
44.06	24291.7
44.08	24083.3
44.1	23675
44.12	24275
44.14	23541.7
44.16	23225
44.18	21600
44.2	22200
44.22	20841.7
44.24	21250
44.26	20733.3
44.28	20541.7
44.3	19783.3
44.32	19283.3
44.34	19558.3
44.36	18575
44.38	18750
44.4	19158.3
44.42	18341.7
44.44	18158.3
44.46	18966.7
44.48	18925
44.5	17916.7
44.52	18708.3

44.54	19366.7
44.56	19616.7
44.58	19916.7
44.6	20108.3
44.62	20016.7
44.64	20758.3
44.66	21391.7
44.68	21433.3
44.7	21850
44.72	23025
44.74	23441.7
44.76	24025
44.78	24733.3
44.8	24408.3
44.82	24783.3
44.84	24725
44.86	25600
44.88	25858.3
44.9	25391.7
44.92	25583.3
44.94	26550
44.96	26408.3
44.98	26766.7
45	26608.3
45.02	26975
45.04	26383.3
45.06	25108.3
45.08	24858.3
45.1	25258.3
45.12	24100
45.14	24083.3
45.16	23358.3
45.18	22750
45.2	22816.7
45.22	22433.3
45.24	22058.3

44.54	34600
44.56	34533.3
44.58	34425
44.6	33558.3
44.62	33950
44.64	32916.7
44.66	34016.7
44.68	34450
44.7	34358.3
44.72	34266.7
44.74	33875
44.76	34291.7
44.78	34325
44.8	33016.7
44.82	33583.3
44.84	34000
44.86	32625
44.88	34066.7
44.9	33583.3
44.92	33841.7
44.94	34400
44.96	33266.7
44.98	33716.7
45	33866.7
45.02	33275
45.04	32916.7
45.06	33508.3
45.08	33908.3
45.1	32900
45.12	33091.7
45.14	33166.7
45.16	32708.3
45.18	33175
45.2	33216.7
45.22	32408.3
45.24	33400

44.54	20408.3
44.56	19975
44.58	19858.3
44.6	20650
44.62	20533.3
44.64	20700
44.66	20483.3
44.68	21150
44.7	21183.3
44.72	22066.7
44.74	22783.3
44.76	23975
44.78	23575
44.8	25041.7
44.82	24583.3
44.84	25133.3
44.86	25383.3
44.88	26000
44.9	26016.7
44.92	26133.3
44.94	26408.3
44.96	26258.3
44.98	26741.7
45	26983.3
45.02	27025
45.04	27300
45.06	26391.7
45.08	27066.7
45.1	26933.3
45.12	26316.7
45.14	25550
45.16	24875
45.18	24291.7
45.2	24216.7
45.22	23608.3
45.24	22975

44.54	18108.3
44.56	18683.3
44.58	18358.3
44.6	18466.7
44.62	18366.7
44.64	18358.3
44.66	18391.7
44.68	18591.7
44.7	18500
44.72	18791.7
44.74	18733.3
44.76	18875
44.78	19066.7
44.8	19358.3
44.82	19775
44.84	19566.7
44.86	20150
44.88	19725
44.9	20066.7
44.92	20200
44.94	21325
44.96	21225
44.98	21433.3
45	21916.7
45.02	22491.7
45.04	22091.7
45.06	22525
45.08	23016.7
45.1	22966.7
45.12	22500
45.14	23041.7
45.16	23333.3
45.18	22516.7
45.2	23158.3
45.22	23575
45.24	22708.3

45.26	21975
45.28	21233.3
45.3	22075
45.32	22333.3
45.34	22550
45.36	22650
45.38	23300
45.4	24208.3
45.42	24733.3
45.44	25450
45.46	26033.3
45.48	26866.7
45.5	26625
45.52	27583.3
45.54	27883.3
45.56	27416.7
45.58	27108.3
45.6	26166.7
45.62	25750
45.64	24100
45.66	24250
45.68	22058.3
45.7	22083.3
45.72	21525
45.74	20400
45.76	20325
45.78	20075
45.8	20075
45.82	20408.3
45.84	20133.3
45.86	20641.7
45.88	19925
45.9	20358.3
45.92	20358.3
45.94	20991.7
45.96	22100

45.26	32450
45.28	32741.7
45.3	32566.7
45.32	33775
45.34	32825
45.36	32658.3
45.38	32958.3
45.4	32533.3
45.42	33083.3
45.44	32091.7
45.46	32166.7
45.48	32283.3
45.5	32091.7
45.52	32100
45.54	32983.3
45.56	32258.3
45.58	32258.3
45.6	32308.3
45.62	32125
45.64	32683.3
45.66	32316.7
45.68	32600
45.7	32816.7
45.72	31866.7
45.74	32733.3
45.76	32283.3
45.78	32283.3
45.8	32333.3
45.82	32391.7
45.84	31608.3
45.86	32125
45.88	32300
45.9	32450
45.92	31783.3
45.94	32675
45.96	32250

45.26	22975
45.28	23008.3
45.3	22983.3
45.32	23183.3
45.34	22983.3
45.36	23633.3
45.38	23941.7
45.4	23516.7
45.42	24783.3
45.44	25550
45.46	26275
45.48	26333.3
45.5	26600
45.52	27208.3
45.54	27108.3
45.56	27183.3
45.58	27483.3
45.6	27800
45.62	27666.7
45.64	27133.3
45.66	24791.7
45.68	24691.7
45.7	23758.3
45.72	23675
45.74	23441.7
45.76	22300
45.78	21483.3
45.8	21375
45.82	20816.7
45.84	20975
45.86	20875
45.88	21225
45.9	21333.3
45.92	21383.3
45.94	21858.3
45.96	22700

45.26	23941.7
45.28	23750
45.3	23075
45.32	23175
45.34	23016.7
45.36	22658.3
45.38	22566.7
45.4	22166.7
45.42	22000
45.44	21875
45.46	21900
45.48	21400
45.5	22358.3
45.52	22166.7
45.54	22425
45.56	22091.7
45.58	22716.7
45.6	22525
45.62	22816.7
45.64	22975
45.66	23183.3
45.68	23091.7
45.7	24200
45.72	24000
45.74	23441.7
45.76	23383.3
45.78	24208.3
45.8	23133.3
45.82	22841.7
45.84	23050
45.86	22916.7
45.88	22683.3
45.9	21700
45.92	21616.7
45.94	20900
45.96	20908.3

45.98	21983.3
46	22608.3
46.02	23575
46.04	24233.3
46.06	25025
46.08	25300
46.1	26841.7
46.12	26525
46.14	25791.7
46.16	26408.3
46.18	26916.7
46.2	27541.7
46.22	27916.7
46.24	27966.7
46.26	29150
46.28	28666.7
46.3	29141.7
46.32	29516.7
46.34	29350
46.36	29808.3
46.38	29900
46.4	30166.7
46.42	29425
46.44	29366.7
46.46	29908.3
46.48	30275
46.5	30108.3
46.52	30050
46.54	30566.7
46.56	30258.3
46.58	29658.3
46.6	31416.7
46.62	31441.7
46.64	30683.3
46.66	31283.3
46.68	31341.7

45.98	31483.3
46	31216.7
46.02	31450
46.04	31500
46.06	32541.7
46.08	32458.3
46.1	31291.7
46.12	31958.3
46.14	32100
46.16	31033.3
46.18	31216.7
46.2	30491.7
46.22	30816.7
46.24	31533.3
46.26	31341.7
46.28	30866.7
46.3	31575
46.32	31933.3
46.34	31008.3
46.36	30891.7
46.38	31141.7
46.4	31133.3
46.42	31050
46.44	30891.7
46.46	31050
46.48	31283.3
46.5	30550
46.52	30183.3
46.54	30608.3
46.56	31066.7
46.58	30833.3
46.6	30891.7
46.62	30375
46.64	31650
46.66	30291.7
46.68	30358.3

45.98	23333.3
46	22575
46.02	23566.7
46.04	24983.3
46.06	25900
46.08	27016.7
46.1	26566.7
46.12	27350
46.14	28466.7
46.16	28133.3
46.18	27616.7
46.2	27766.7
46.22	28741.7
46.24	29008.3
46.26	27775
46.28	28558.3
46.3	28741.7
46.32	28433.3
46.34	28591.7
46.36	29291.7
46.38	29358.3
46.4	28491.7
46.42	28450
46.44	28800
46.46	28175
46.48	29300
46.5	29216.7
46.52	29450
46.54	29258.3
46.56	29341.7
46.58	29391.7
46.6	30050
46.62	30641.7
46.64	30175
46.66	31333.3
46.68	31600

45.98	20366.7
46	20866.7
46.02	20075
46.04	19250
46.06	19641.7
46.08	19283.3
46.1	20250
46.12	19766.7
46.14	19983.3
46.16	20858.3
46.18	20458.3
46.2	20433.3
46.22	21308.3
46.24	22091.7
46.26	22233.3
46.28	22933.3
46.3	22708.3
46.32	23783.3
46.34	23650
46.36	23400
46.38	23733.3
46.4	24458.3
46.42	24391.7
46.44	24441.7
46.46	24350
46.48	24258.3
46.5	24191.7
46.52	24066.7
46.54	24600
46.56	24050
46.58	23550
46.6	23650
46.62	23841.7
46.64	23808.3
46.66	23566.7
46.68	23483.3

46.7	31075
46.72	30725
46.74	29800
46.76	28925
46.78	28541.7
46.8	26866.7
46.82	26441.7
46.84	25425
46.86	24608.3
46.88	24641.7
46.9	24266.7
46.92	24008.3
46.94	23558.3
46.96	22450
46.98	23533.3
47	23591.7
47.02	23133.3
47.04	23733.3
47.06	23808.3
47.08	24816.7
47.1	25425
47.12	25833.3
47.14	26216.7
47.16	26883.3
47.18	27166.7
47.2	28500
47.22	29391.7
47.24	29991.7
47.26	31625
47.28	32500
47.3	34783.3
47.32	34908.3
47.34	35350
47.36	36108.3
47.38	35491.7
47.4	34616.7

46.7	30033.3
46.72	30525
46.74	30675
46.76	31366.7
46.78	30200
46.8	30575
46.82	30566.7
46.84	30708.3
46.86	30316.7
46.88	30900
46.9	29833.3
46.92	30600
46.94	30333.3
46.96	29741.7
46.98	29325
47	30116.7
47.02	29758.3
47.04	30133.3
47.06	29908.3
47.08	30500
47.1	29558.3
47.12	29333.3
47.14	30500
47.16	29958.3
47.18	30383.3
47.2	29633.3
47.22	28991.7
47.24	29425
47.26	30183.3
47.28	29350
47.3	30216.7
47.32	30458.3
47.34	30341.7
47.36	29925
47.38	29725
47.4	29300

46.7	31383.3
46.72	31650
46.74	31516.7
46.76	31533.3
46.78	30800
46.8	30691.7
46.82	28641.7
46.84	27400
46.86	26800
46.88	25725
46.9	25508.3
46.92	24383.3
46.94	23591.7
46.96	23608.3
46.98	22633.3
47	22966.7
47.02	23266.7
47.04	23025
47.06	23383.3
47.08	23291.7
47.1	23816.7
47.12	24016.7
47.14	24800
47.16	24950
47.18	25891.7
47.2	25750
47.22	26000
47.24	26500
47.26	28500
47.28	28758.3
47.3	29458.3
47.32	30600
47.34	31258.3
47.36	30908.3
47.38	32075
47.4	32291.7

46.7	23191.7
46.72	24050
46.74	24308.3
46.76	24258.3
46.78	23941.7
46.8	24483.3
46.82	24058.3
46.84	24283.3
46.86	24666.7
46.88	24866.7
46.9	25025
46.92	25008.3
46.94	24725
46.96	25275
46.98	25216.7
47	25566.7
47.02	24741.7
47.04	24800
47.06	24166.7
47.08	23833.3
47.1	22608.3
47.12	23058.3
47.14	23266.7
47.16	21983.3
47.18	21491.7
47.2	21141.7
47.22	20666.7
47.24	21000
47.26	20825
47.28	20441.7
47.3	21633.3
47.32	20841.7
47.34	20633.3
47.36	20208.3
47.38	20825
47.4	22333.3

47.42	33241.7
47.44	32283.3
47.46	31750
47.48	30500
47.5	28716.7
47.52	28233.3
47.54	27641.7
47.56	25583.3
47.58	24841.7
47.6	23066.7
47.62	21916.7
47.64	21791.7
47.66	20883.3
47.68	20616.7
47.7	20766.7
47.72	20450
47.74	19808.3
47.76	19675
47.78	18858.3
47.8	19516.7
47.82	19433.3
47.84	19583.3
47.86	19300
47.88	19716.7
47.9	19500
47.92	19783.3
47.94	20908.3
47.96	20033.3
47.98	19908.3
48	19958.3
48.02	20016.7
48.04	20291.7
48.06	19608.3
48.08	19591.7
48.1	19958.3
48.12	19458.3

47.42	29708.3
47.44	29266.7
47.46	29083.3
47.48	29441.7
47.5	29433.3
47.52	28816.7
47.54	29275
47.56	28683.3
47.58	28925
47.6	29658.3
47.62	29225
47.64	29566.7
47.66	29375
47.68	29608.3
47.7	28983.3
47.72	28666.7
47.74	29216.7
47.76	28566.7
47.78	27941.7
47.8	29341.7
47.82	28166.7
47.84	28600
47.86	28700
47.88	28625
47.9	28391.7
47.92	29175
47.94	28275
47.96	28766.7
47.98	28141.7
48	28091.7
48.02	28600
48.04	28508.3
48.06	28450
48.08	28300
48.1	28066.7
48.12	27758.3

47.42	32791.7
47.44	31116.7
47.46	30291.7
47.48	30166.7
47.5	30291.7
47.52	28558.3
47.54	28033.3
47.56	26225
47.58	25325
47.6	23900
47.62	23075
47.64	22108.3
47.66	22283.3
47.68	21066.7
47.7	20883.3
47.72	20475
47.74	20283.3
47.76	19850
47.78	19050
47.8	18991.7
47.82	19516.7
47.84	19141.7
47.86	19125
47.88	18941.7
47.9	19358.3
47.92	19950
47.94	20050
47.96	19325
47.98	20058.3
48	19933.3
48.02	19283.3
48.04	20033.3
48.06	19075
48.08	19416.7
48.1	19516.7
48.12	19425

47.42	20933.3
47.44	21175
47.46	21108.3
47.48	21683.3
47.5	22075
47.52	22750
47.54	22891.7
47.56	22858.3
47.58	22150
47.6	23200
47.62	22925
47.64	23091.7
47.66	22883.3
47.68	22616.7
47.7	22333.3
47.72	21875
47.74	22325
47.76	20916.7
47.78	21350
47.8	20925
47.82	20191.7
47.84	19958.3
47.86	19591.7
47.88	19841.7
47.9	19383.3
47.92	19116.7
47.94	19750
47.96	18658.3
47.98	17966.7
48	18200
48.02	17983.3
48.04	18125
48.06	18050
48.08	17525
48.1	17650
48.12	17591.7

48.14	18983.3
48.16	19225
48.18	18758.3
48.2	19408.3
48.22	19408.3
48.24	19616.7
48.26	20233.3
48.28	20100
48.3	21550
48.32	21900
48.34	22783.3
48.36	23491.7
48.38	24475
48.4	25641.7
48.42	26350
48.44	28450
48.46	29733.3
48.48	30458.3
48.5	31808.3
48.52	33550
48.54	34900
48.56	35375
48.58	35658.3
48.6	35841.7
48.62	35325
48.64	34950
48.66	33933.3
48.68	32400
48.7	31700
48.72	30500
48.74	29100
48.76	27900
48.78	27550
48.8	26200
48.82	25700
48.84	26316.7

48.14	27900
48.16	28041.7
48.18	28400
48.2	28208.3
48.22	28358.3
48.24	27633.3
48.26	27983.3
48.28	29166.7
48.3	28325
48.32	28583.3
48.34	27941.7
48.36	27575
48.38	27816.7
48.4	28225
48.42	28175
48.44	28558.3
48.46	28608.3
48.48	27383.3
48.5	27983.3
48.52	27816.7
48.54	26841.7
48.56	28666.7
48.58	28066.7
48.6	27808.3
48.62	27841.7
48.64	27750
48.66	27525
48.68	27600
48.7	27458.3
48.72	27283.3
48.74	27491.7
48.76	27283.3
48.78	27258.3
48.8	27683.3
48.82	27700
48.84	26441.7

48.14	19075
48.16	19541.7
48.18	19800
48.2	19983.3
48.22	19141.7
48.24	20191.7
48.26	19475
48.28	20250
48.3	20275
48.32	20541.7
48.34	21866.7
48.36	22425
48.38	22800
48.4	24116.7
48.42	25483.3
48.44	26291.7
48.46	27491.7
48.48	28425
48.5	29825
48.52	30691.7
48.54	31700
48.56	33158.3
48.58	33708.3
48.6	34608.3
48.62	35008.3
48.64	36458.3
48.66	35008.3
48.68	34216.7
48.7	33283.3
48.72	32500
48.74	30900
48.76	30291.7
48.78	28866.7
48.8	28891.7
48.82	28458.3
48.84	26125

48.14	17658.3
48.16	17925
48.18	17475
48.2	17591.7
48.22	17400
48.24	17416.7
48.26	17258.3
48.28	17858.3
48.3	17658.3
48.32	18058.3
48.34	17691.7
48.36	17975
48.38	18050
48.4	17800
48.42	18425
48.44	18175
48.46	18100
48.48	19350
48.5	18600
48.52	18500
48.54	19158.3
48.56	19858.3
48.58	20116.7
48.6	20708.3
48.62	20183.3
48.64	22308.3
48.66	22950
48.68	23116.7
48.7	23616.7
48.72	24116.7
48.74	23950
48.76	25100
48.78	25158.3
48.8	24825
48.82	25950
48.84	26116.7

48.86	25616.7
48.88	24683.3
48.9	26041.7
48.92	25508.3
48.94	26641.7
48.96	26925
48.98	28175
49	28833.3
49.02	28708.3
49.04	29966.7
49.06	30433.3
49.08	30425
49.1	30050
49.12	30450
49.14	29400
49.16	27791.7
49.18	28450
49.2	27316.7
49.22	26675
49.24	26975
49.26	25716.7
49.28	25350
49.3	25783.3
49.32	25341.7
49.34	25175
49.36	26066.7
49.38	25400
49.4	26050
49.42	26275
49.44	25641.7
49.46	25725
49.48	25183.3
49.5	25633.3
49.52	26533.3
49.54	26266.7
49.56	27216.7

48.86	27650
48.88	26933.3
48.9	27241.7
48.92	27325
48.94	26933.3
48.96	27258.3
48.98	27883.3
49	27433.3
49.02	27550
49.04	27033.3
49.06	26708.3
49.08	26383.3
49.1	26491.7
49.12	26491.7
49.14	26558.3
49.16	26558.3
49.18	26558.3
49.2	26441.7
49.22	27008.3
49.24	26650
49.26	26250
49.28	26341.7
49.3	26558.3
49.32	26816.7
49.34	26808.3
49.36	26558.3
49.38	26158.3
49.4	26225
49.42	26716.7
49.44	26883.3
49.46	26225
49.48	26091.7
49.5	26216.7
49.52	27208.3
49.54	26183.3
49.56	26283.3

48.86	25816.7
48.88	25275
48.9	25325
48.92	25675
48.94	25558.3
48.96	26408.3
48.98	27366.7
49	27775
49.02	28791.7
49.04	28166.7
49.06	29850
49.08	30508.3
49.1	31308.3
49.12	31266.7
49.14	30708.3
49.16	29808.3
49.18	28741.7
49.2	28608.3
49.22	27708.3
49.24	27291.7
49.26	27758.3
49.28	25958.3
49.3	25941.7
49.32	25883.3
49.34	26091.7
49.36	26433.3
49.38	26700
49.4	26133.3
49.42	26408.3
49.44	26800
49.46	26825
49.48	26741.7
49.5	27466.7
49.52	26483.3
49.54	26858.3
49.56	27008.3

48.86	25183.3
48.88	26550
48.9	25808.3
48.92	25783.3
48.94	24883.3
48.96	24475
48.98	23808.3
49	23666.7
49.02	22925
49.04	23283.3
49.06	23550
49.08	22633.3
49.1	23058.3
49.12	22350
49.14	22183.3
49.16	22816.7
49.18	22900
49.2	22658.3
49.22	23591.7
49.24	23500
49.26	24116.7
49.28	24583.3
49.3	23608.3
49.32	24225
49.34	24550
49.36	23900
49.38	24108.3
49.4	23700
49.42	23625
49.44	23075
49.46	23558.3
49.48	23841.7
49.5	22975
49.52	23833.3
49.54	22966.7
49.56	22816.7

49.58	27233.3
49.6	27083.3
49.62	26783.3
49.64	26275
49.66	26733.3
49.68	26650
49.7	26733.3
49.72	26441.7
49.74	26158.3
49.76	26791.7
49.78	26900
49.8	26908.3
49.82	26616.7
49.84	26825
49.86	26758.3
49.88	25775
49.9	25958.3
49.92	25466.7
49.94	24591.7
49.96	24025
49.98	23641.7
50	22750
50.02	21458.3
50.04	21058.3
50.06	21433.3
50.08	20675
50.1	20483.3
50.12	20608.3
50.14	20991.7
50.16	20625
50.18	20566.7
50.2	21975
50.22	21983.3
50.24	22150
50.26	22450
50.28	23908.3

49.58	25766.7
49.6	26491.7
49.62	26891.7
49.64	26925
49.66	27191.7
49.68	26475
49.7	26191.7
49.72	25600
49.74	26058.3
49.76	25675
49.78	26141.7
49.8	26116.7
49.82	26016.7
49.84	25575
49.86	25891.7
49.88	25608.3
49.9	25850
49.92	26508.3
49.94	26433.3
49.96	26341.7
49.98	25766.7
50	25450
50.02	25916.7
50.04	26033.3
50.06	26225
50.08	26008.3
50.1	26683.3
50.12	26850
50.14	26075
50.16	26125
50.18	25216.7
50.2	25625
50.22	25325
50.24	25541.7
50.26	24916.7
50.28	25825

49.58	27141.7
49.6	28016.7
49.62	28266.7
49.64	27483.3
49.66	27925
49.68	27266.7
49.7	27833.3
49.72	26433.3
49.74	27200
49.76	27075
49.78	26200
49.8	26366.7
49.82	26150
49.84	26041.7
49.86	25866.7
49.88	26041.7
49.9	26166.7
49.92	24883.3
49.94	24766.7
49.96	25125
49.98	24516.7
50	23241.7
50.02	22558.3
50.04	21708.3
50.06	21491.7
50.08	21883.3
50.1	20850
50.12	21858.3
50.14	21183.3
50.16	21583.3
50.18	21400
50.2	21316.7
50.22	21525
50.24	21116.7
50.26	22300
50.28	23183.3

49.58	22500
49.6	22625
49.62	22983.3
49.64	23516.7
49.66	23100
49.68	22775
49.7	23475
49.72	23266.7
49.74	23950
49.76	24016.7
49.78	23533.3
49.8	24116.7
49.82	24125
49.84	25133.3
49.86	24850
49.88	24958.3
49.9	24666.7
49.92	24725
49.94	24341.7
49.96	24600
49.98	25116.7
50	25075
50.02	25033.3
50.04	26191.7
50.06	26350
50.08	26816.7
50.1	28250
50.12	28158.3
50.14	28658.3
50.16	28508.3
50.18	28608.3
50.2	29266.7
50.22	28966.7
50.24	29350
50.26	29850
50.28	29816.7

50.3	23641.7
50.32	23566.7
50.34	24458.3
50.36	24500
50.38	25583.3
50.4	26525
50.42	27133.3
50.44	27491.7
50.46	27758.3
50.48	28366.7
50.5	28100
50.52	28316.7
50.54	28725
50.56	27725
50.58	28466.7
50.6	29016.7
50.62	28800
50.64	27458.3
50.66	27241.7
50.68	27025
50.7	26575
50.72	25766.7
50.74	26291.7
50.76	25233.3
50.78	24916.7
50.8	24183.3
50.82	24050
50.84	23866.7
50.86	23725
50.88	23575
50.9	22875
50.92	22908.3
50.94	22500
50.96	23558.3
50.98	23475
51	23258.3

50.3	25750
50.32	25483.3
50.34	25183.3
50.36	24933.3
50.38	24475
50.4	24883.3
50.42	25441.7
50.44	25125
50.46	25041.7
50.48	25008.3
50.5	25083.3
50.52	24591.7
50.54	25416.7
50.56	24966.7
50.58	24558.3
50.6	25300
50.62	25041.7
50.64	24833.3
50.66	24666.7
50.68	24850
50.7	24308.3
50.72	24275
50.74	24975
50.76	24916.7
50.78	24983.3
50.8	25308.3
50.82	25025
50.84	25333.3
50.86	24616.7
50.88	25225
50.9	24233.3
50.92	24141.7
50.94	25266.7
50.96	24633.3
50.98	24225
51	24883.3

50.3	23825
50.32	24125
50.34	23783.3
50.36	24641.7
50.38	25391.7
50.4	26433.3
50.42	26533.3
50.44	26691.7
50.46	26700
50.48	27133.3
50.5	28491.7
50.52	28408.3
50.54	28108.3
50.56	28608.3
50.58	28333.3
50.6	28883.3
50.62	27441.7
50.64	27250
50.66	26866.7
50.68	26491.7
50.7	25833.3
50.72	25875
50.74	25975
50.76	25383.3
50.78	24575
50.8	24391.7
50.82	24566.7
50.84	24258.3
50.86	24291.7
50.88	23858.3
50.9	23641.7
50.92	23766.7
50.94	22933.3
50.96	22941.7
50.98	23175
51	22300

50.3	27900
50.32	27158.3
50.34	27300
50.36	26741.7
50.38	26508.3
50.4	25808.3
50.42	25508.3
50.44	25808.3
50.46	25258.3
50.48	24966.7
50.5	25250
50.52	25466.7
50.54	25916.7
50.56	25000
50.58	25500
50.6	25175
50.62	25450
50.64	25725
50.66	26658.3
50.68	26600
50.7	25866.7
50.72	26366.7
50.74	26633.3
50.76	26766.7
50.78	26633.3
50.8	25866.7
50.82	25108.3
50.84	25000
50.86	24450
50.88	23608.3
50.9	23475
50.92	22716.7
50.94	22266.7
50.96	21600
50.98	21683.3
51	20541.7

51.02	23741.7
51.04	24183.3
51.06	24991.7
51.08	25141.7
51.1	25583.3
51.12	26658.3
51.14	27258.3
51.16	27808.3
51.18	27233.3
51.2	27750
51.22	28891.7
51.24	29375
51.26	29083.3
51.28	29083.3
51.3	29566.7
51.32	30266.7
51.34	30200
51.36	30308.3
51.38	29625
51.4	29108.3
51.42	29808.3
51.44	28308.3
51.46	27600
51.48	26925
51.5	25900
51.52	24750
51.54	23750
51.56	23141.7
51.58	22491.7
51.6	22091.7
51.62	21383.3
51.64	20191.7
51.66	20175
51.68	19950
51.7	19941.7
51.72	19591.7

51.02	23775
51.04	24433.3
51.06	24383.3
51.08	24416.7
51.1	24733.3
51.12	24191.7
51.14	24333.3
51.16	24275
51.18	24225
51.2	24233.3
51.22	23958.3
51.24	24500
51.26	24900
51.28	24383.3
51.3	24066.7
51.32	23833.3
51.34	24033.3
51.36	24450
51.38	23958.3
51.4	23733.3
51.42	24166.7
51.44	24333.3
51.46	23941.7
51.48	24266.7
51.5	24266.7
51.52	23833.3
51.54	23608.3
51.56	24016.7
51.58	22675
51.6	23725
51.62	23191.7
51.64	23875
51.66	24058.3
51.68	23500
51.7	23616.7
51.72	23633.3

51.02	23191.7
51.04	23616.7
51.06	23683.3
51.08	25041.7
51.1	24791.7
51.12	25133.3
51.14	25816.7
51.16	26941.7
51.18	27350
51.2	27800
51.22	28691.7
51.24	28841.7
51.26	28283.3
51.28	28641.7
51.3	28766.7
51.32	28550
51.34	29308.3
51.36	29691.7
51.38	29941.7
51.4	29191.7
51.42	29758.3
51.44	28908.3
51.46	29016.7
51.48	28525
51.5	27158.3
51.52	26200
51.54	24625
51.56	24050
51.58	23891.7
51.6	23191.7
51.62	22575
51.64	21583.3
51.66	21166.7
51.68	20833.3
51.7	20458.3
51.72	20300

51.02	21258.3
51.04	21533.3
51.06	21158.3
51.08	20850
51.1	20891.7
51.12	20475
51.14	20925
51.16	20875
51.18	20225
51.2	20158.3
51.22	20200
51.24	20983.3
51.26	20541.7
51.28	20491.7
51.3	21191.7
51.32	21383.3
51.34	21241.7
51.36	21791.7
51.38	22183.3
51.4	22016.7
51.42	22541.7
51.44	22116.7
51.46	22008.3
51.48	22158.3
51.5	22858.3
51.52	23183.3
51.54	23625
51.56	23233.3
51.58	23608.3
51.6	23466.7
51.62	21725
51.64	22566.7
51.66	22466.7
51.68	21691.7
51.7	21766.7
51.72	21533.3

51.74	19016.7
51.76	19316.7
51.78	19633.3
51.8	19475
51.82	20308.3
51.84	20750
51.86	20866.7
51.88	20825
51.9	21200
51.92	21775
51.94	22166.7
51.96	22333.3
51.98	22633.3
52	22833.3
52.02	23600
52.04	23475
52.06	23766.7
52.08	23808.3
52.1	23600
52.12	23541.7
52.14	23558.3
52.16	23575
52.18	23216.7
52.2	22925
52.22	23216.7
52.24	23200
52.26	23658.3
52.28	23783.3
52.3	23925
52.32	24300
52.34	24350
52.36	24358.3
52.38	25141.7
52.4	25908.3
52.42	26283.3
52.44	26175

51.74	23908.3
51.76	23558.3
51.78	23266.7
51.8	23583.3
51.82	23891.7
51.84	24033.3
51.86	23291.7
51.88	23591.7
51.9	23841.7
51.92	23441.7
51.94	23683.3
51.96	23850
51.98	23458.3
52	23808.3
52.02	23700
52.04	23983.3
52.06	23550
52.08	23350
52.1	22716.7
52.12	23650
52.14	23725
52.16	23125
52.18	23641.7
52.2	23608.3
52.22	22883.3
52.24	23558.3
52.26	23616.7
52.28	23175
52.3	23100
52.32	23366.7
52.34	22808.3
52.36	22741.7
52.38	22775
52.4	22908.3
52.42	23150
52.44	22908.3

51.74	19508.3
51.76	20408.3
51.78	20441.7
51.8	20441.7
51.82	20333.3
51.84	20383.3
51.86	19883.3
51.88	20258.3
51.9	20941.7
51.92	21016.7
51.94	21608.3
51.96	21441.7
51.98	22241.7
52	22233.3
52.02	21941.7
52.04	23200
52.06	23250
52.08	23791.7
52.1	23133.3
52.12	23216.7
52.14	23533.3
52.16	23575
52.18	23925
52.2	23641.7
52.22	23850
52.24	23900
52.26	23891.7
52.28	24125
52.3	24066.7
52.32	24441.7
52.34	24625
52.36	25091.7
52.38	25125
52.4	24725
52.42	25075
52.44	25616.7

51.74	20916.7
51.76	20583.3
51.78	20608.3
51.8	19566.7
51.82	19083.3
51.84	19025
51.86	18783.3
51.88	19016.7
51.9	18558.3
51.92	18600
51.94	18466.7
51.96	18641.7
51.98	18616.7
52	18033.3
52.02	17658.3
52.04	19016.7
52.06	18575
52.08	18383.3
52.1	18825
52.12	18333.3
52.14	18700
52.16	18741.7
52.18	18666.7
52.2	19208.3
52.22	19516.7
52.24	19608.3
52.26	19425
52.28	19641.7
52.3	19775
52.32	20200
52.34	19991.7
52.36	19650
52.38	19691.7
52.4	20250
52.42	19691.7
52.44	20258.3

52.46	26691.7
52.48	26941.7
52.5	27250
52.52	27150
52.54	27500
52.56	27675
52.58	27791.7
52.6	28275
52.62	29433.3
52.64	29691.7
52.66	29608.3
52.68	29091.7
52.7	28608.3
52.72	28158.3
52.74	27458.3
52.76	27008.3
52.78	26383.3
52.8	25241.7
52.82	24633.3
52.84	23700
52.86	23041.7
52.88	22150
52.9	20875
52.92	20525
52.94	20241.7
52.96	20033.3
52.98	19083.3
53	18800
53.02	19008.3
53.04	19116.7
53.06	19483.3
53.08	19141.7
53.1	19208.3
53.12	18816.7
53.14	18916.7
53.16	19333.3

52.46	23000
52.48	23541.7
52.5	22916.7
52.52	22816.7
52.54	22775
52.56	22600
52.58	22458.3
52.6	22300
52.62	22683.3
52.64	23250
52.66	23200
52.68	23350
52.7	22991.7
52.72	22475
52.74	22600
52.76	23041.7
52.78	22733.3
52.8	22325
52.82	22400
52.84	23058.3
52.86	22183.3
52.88	23041.7
52.9	22608.3
52.92	22141.7
52.94	21758.3
52.96	22275
52.98	21975
53	22116.7
53.02	22983.3
53.04	22958.3
53.06	22591.7
53.08	22316.7
53.1	22541.7
53.12	23066.7
53.14	22708.3
53.16	22750

52.46	26091.7
52.48	26466.7
52.5	26533.3
52.52	27241.7
52.54	27250
52.56	27008.3
52.58	28091.7
52.6	28541.7
52.62	28716.7
52.64	28841.7
52.66	28483.3
52.68	28850
52.7	29516.7
52.72	29783.3
52.74	29600
52.76	28950
52.78	29016.7
52.8	27833.3
52.82	26858.3
52.84	25991.7
52.86	23941.7
52.88	23316.7
52.9	22683.3
52.92	22716.7
52.94	21725
52.96	21183.3
52.98	19783.3
53	20066.7
53.02	20583.3
53.04	20433.3
53.06	19125
53.08	18525
53.1	18616.7
53.12	19250
53.14	19258.3
53.16	19300

52.46	20425
52.48	20225
52.5	20725
52.52	20491.7
52.54	21075
52.56	20791.7
52.58	20716.7
52.6	20641.7
52.62	20275
52.64	21125
52.66	21433.3
52.68	21625
52.7	21816.7
52.72	21625
52.74	21441.7
52.76	21750
52.78	22350
52.8	22200
52.82	22183.3
52.84	22858.3
52.86	22491.7
52.88	22733.3
52.9	22616.7
52.92	22366.7
52.94	22900
52.96	22250
52.98	21891.7
53	21491.7
53.02	20458.3
53.04	20900
53.06	20841.7
53.08	20025
53.1	19908.3
53.12	19641.7
53.14	19700
53.16	18833.3

53.18	19241.7
53.2	18783.3
53.22	18625
53.24	18358.3
53.26	18841.7
53.28	18225
53.3	17933.3
53.32	18108.3
53.34	18266.7
53.36	18200
53.38	18408.3
53.4	18100
53.42	17825
53.44	17875
53.46	18183.3
53.48	18491.7
53.5	17700
53.52	17700
53.54	17566.7
53.56	17883.3
53.58	17783.3
53.6	18575
53.62	17708.3
53.64	17683.3
53.66	17775
53.68	18050
53.7	18075
53.72	18050
53.74	17575
53.76	17408.3
53.78	18283.3
53.8	18375
53.82	18591.7
53.84	18600
53.86	18283.3
53.88	18425

53.18	22100
53.2	22250
53.22	22566.7
53.24	21816.7
53.26	21458.3
53.28	22366.7
53.3	22133.3
53.32	22275
53.34	22208.3
53.36	21833.3
53.38	22475
53.4	21825
53.42	22175
53.44	22141.7
53.46	22291.7
53.48	22083.3
53.5	22216.7
53.52	21925
53.54	22700
53.56	21991.7
53.58	21866.7
53.6	21983.3
53.62	22166.7
53.64	22100
53.66	21808.3
53.68	22016.7
53.7	21925
53.72	22100
53.74	22266.7
53.76	22450
53.78	22075
53.8	22358.3
53.82	22000
53.84	21708.3
53.86	21958.3
53.88	22075

53.18	19333.3
53.2	18900
53.22	18350
53.24	18650
53.26	18825
53.28	18533.3
53.3	18591.7
53.32	18533.3
53.34	17966.7
53.36	18308.3
53.38	18241.7
53.4	17783.3
53.42	18075
53.44	18708.3
53.46	18175
53.48	17916.7
53.5	18150
53.52	18525
53.54	18983.3
53.56	18950
53.58	18266.7
53.6	18266.7
53.62	18875
53.64	18633.3
53.66	17933.3
53.68	17883.3
53.7	17791.7
53.72	18233.3
53.74	17983.3
53.76	17500
53.78	18066.7
53.8	18283.3
53.82	17908.3
53.84	17950
53.86	17966.7
53.88	17775

53.18	18650
53.2	18341.7
53.22	18075
53.24	18241.7
53.26	18416.7
53.28	17691.7
53.3	17966.7
53.32	17758.3
53.34	17916.7
53.36	17941.7
53.38	17583.3
53.4	17508.3
53.42	17383.3
53.44	17366.7
53.46	17275
53.48	17200
53.5	16958.3
53.52	17008.3
53.54	17400
53.56	17116.7
53.58	16875
53.6	17350
53.62	16566.7
53.64	16808.3
53.66	16575
53.68	17058.3
53.7	17075
53.72	17116.7
53.74	15850
53.76	16491.7
53.78	16283.3
53.8	16750
53.82	16741.7
53.84	16466.7
53.86	16575
53.88	16425

53.9	18616.7	53.9	22250	53.9	17950	53.9	16491.7
53.92	18175	53.92	21950	53.92	18425	53.92	16708.3
53.94	18908.3	53.94	21716.7	53.94	18308.3	53.94	16516.7
53.96	18883.3	53.96	21466.7	53.96	19091.7	53.96	16625
53.98	19383.3	53.98	21950	53.98	18533.3	53.98	16341.7
54	19191.7	54	21416.7	54	18791.7	54	16216.7
54.02	19058.3	54.02	21783.3	54.02	19425	54.02	16425
54.04	19000	54.04	21741.7	54.04	18758.3	54.04	15991.7
54.06	19083.3	54.06	21758.3	54.06	19125	54.06	16066.7
54.08	19383.3	54.08	21666.7	54.08	19425	54.08	16933.3
54.1	20050	54.1	21666.7	54.1	19350	54.1	16325
54.12	19558.3	54.12	22008.3	54.12	19391.7	54.12	16083.3
54.14	19900	54.14	21908.3	54.14	19850	54.14	16200
54.16	19433.3	54.16	21041.7	54.16	19875	54.16	16866.7
54.18	19616.7	54.18	21283.3	54.18	20275	54.18	16383.3
54.2	19816.7	54.2	21375	54.2	20383.3	54.2	16116.7
54.22	19916.7	54.22	21816.7	54.22	20175	54.22	16400
54.24	19666.7	54.24	21291.7	54.24	20000	54.24	16566.7
54.26	19950	54.26	21383.3	54.26	19675	54.26	16800
54.28	19883.3	54.28	21716.7	54.28	20225	54.28	17125
54.3	19841.7	54.3	21575	54.3	20158.3	54.3	16650
54.32	19733.3	54.32	21225	54.32	20083.3	54.32	16800
54.34	19541.7	54.34	20991.7	54.34	19633.3	54.34	17433.3
54.36	19666.7	54.36	21450	54.36	19191.7	54.36	17575
54.38	20216.7	54.38	21341.7	54.38	19808.3	54.38	17275
54.4	20533.3	54.4	21341.7	54.4	20166.7	54.4	17291.7
54.42	20675	54.42	21291.7	54.42	20241.7	54.42	17425
54.44	20258.3	54.44	20866.7	54.44	20233.3	54.44	17258.3
54.46	20183.3	54.46	21300	54.46	20250	54.46	17766.7
54.48	20216.7	54.48	21341.7	54.48	20741.7	54.48	17450
54.5	20250	54.5	21516.7	54.5	21000	54.5	17550
54.52	20891.7	54.52	21683.3	54.52	21441.7	54.52	17650
54.54	21508.3	54.54	21666.7	54.54	21641.7	54.54	17475
54.56	20975	54.56	21308.3	54.56	21608.3	54.56	17300
54.58	21283.3	54.58	21241.7	54.58	21216.7	54.58	17783.3
54.6	21433.3	54.6	21716.7	54.6	21966.7	54.6	17908.3

54.62	21808.3
54.64	22725
54.66	22775
54.68	23333.3
54.7	23983.3
54.72	23650
54.74	24008.3
54.76	24691.7
54.78	24891.7
54.8	25058.3
54.82	24600
54.84	24375
54.86	23841.7
54.88	23383.3
54.9	23300
54.92	22791.7
54.94	23150
54.96	22308.3
54.98	22641.7
55	22350

54.62	21641.7
54.64	21050
54.66	21216.7
54.68	20925
54.7	21750
54.72	21125
54.74	21883.3
54.76	21100
54.78	21433.3
54.8	21591.7
54.82	21341.7
54.84	21275
54.86	21683.3
54.88	21500
54.9	21116.7
54.92	20966.7
54.94	21450
54.96	21083.3
54.98	20916.7
55	21250

54.62	21966.7
54.64	22366.7
54.66	22825
54.68	22725
54.7	22458.3
54.72	23033.3
54.74	23525
54.76	24075
54.78	24150
54.8	24175
54.82	24275
54.84	24350
54.86	23825
54.88	23850
54.9	23425
54.92	23433.3
54.94	23416.7
54.96	23000
54.98	22600
55	22183.3

54.62	17525
54.64	17308.3
54.66	17608.3
54.68	17758.3
54.7	18141.7
54.72	18600
54.74	18258.3
54.76	18383.3
54.78	18550
54.8	18533.3
54.82	18875
54.84	18983.3
54.86	18891.7
54.88	18791.7
54.9	18891.7
54.92	19108.3
54.94	18950
54.96	18900
54.98	19058.3
55	18875
