# Supplementary material for: Dermal formulation based on carbopol and Gum Arabic improves skin retention of indomethacin
Source: PLoS One. 2025 Jun 10;20(6):e0326051. doi: 10.1371/journal.pone.0326051 (PMC12151425; doi:10.1371/journal.pone.0326051)
Supplement: S2 Table — Raw data in Fig 2C and 2D. (PDF) [file pone.0326051.s004.pdf]

Figure 2C

Time	Temp	DTA	TGA
sec	˚ C	uV	mg
0	33.97	-0.66	4.53
1	33.94	-0.67	4.53
2	34.08	-0.67	4.53
3	34.07	-0.67	4.53
4	34.24	-0.67	4.53
5	34.04	-0.69	4.53
6	34.04	-0.69	4.53
7	34.02	-0.69	4.53
8	34.14	-0.69	4.53
9	34.2	-0.67	4.53
10	34.24	-0.69	4.53
11	34.12	-0.69	4.53
12	34.05	-0.69	4.53
13	34.15	-0.69	4.53
14	34.15	-0.69	4.53
15	34.32	-0.69	4.53
16	34.21	-0.69	4.53
17	34.15	-0.70	4.53
18	34.09	-0.69	4.53
19	34.26	-0.69	4.53
20	34.21	-0.69	4.53
21	34.41	-0.69	4.53
22	34.23	-0.69	4.53
23	34.16	-0.70	4.53
24	34.19	-0.69	4.53
25	34.29	-0.69	4.53
26	34.34	-0.69	4.53
27	34.37	-0.69	4.53
28	34.31	-0.69	4.53
29	34.16	-0.69	4.53
30	34.29	-0.69	4.53
31	34.26	-0.69	4.53

Figure 2D

Vehicle

Time	Temp	DTA	TGA
sec	˚ C	uV	mg
0	33.66	-0.40	4.87
1	33.44	-0.40	4.87
2	33.51	-0.40	4.87
3	33.56	-0.39	4.87
4	33.63	-0.40	4.87
5	33.74	-0.39	4.86
6	33.6	-0.40	4.86
7	33.52	-0.39	4.86
8	33.48	-0.39	4.86
9	33.65	-0.39	4.86
10	33.64	-0.39	4.86
11	33.79	-0.40	4.86
12	33.57	-0.40	4.86
13	33.59	-0.39	4.86
14	33.57	-0.40	4.86
15	33.69	-0.39	4.86
16	33.76	-0.40	4.86
17	33.74	-0.40	4.86
18	33.61	-0.40	4.86
19	33.59	-0.40	4.86
20	33.69	-0.39	4.86
21	33.66	-0.40	4.86
22	33.83	-0.40	4.86
23	33.68	-0.40	4.86
24	33.65	-0.40	4.86
25	33.58	-0.40	4.86
26	33.77	-0.39	4.86
27	33.75	-0.39	4.86
28	33.85	-0.39	4.86
29	33.69	-0.40	4.86
30	33.65	-0.40	4.86
31	33.71	-0.40	4.86

IMC-MP@GCgel

Time	Temp	DTA	TGA
sec	˚ C	uV	mg
0	25.78	-0.54	4.3475
1	25.87	-0.54	4.3475
2	25.89	-0.52	4.3475
3	25.94	-0.54	4.3475
4	26.05	-0.52	4.3475
5	25.91	-0.54	4.3475
6	25.81	-0.54	4.3475
7	25.78	-0.54	4.3475
8	25.94	-0.54	4.3475
9	25.92	-0.54	4.3475
10	26.07	-0.52	4.3475
11	25.85	-0.54	4.3475
12	25.85	-0.54	4.3475
13	25.82	-0.54	4.3475
14	25.92	-0.54	4.3475
15	25.97	-0.54	4.3475
16	26.02	-0.54	4.3475
17	25.86	-0.52	4.3475
18	25.76	-0.54	4.3475
19	25.88	-0.52	4.3475
20	25.87	-0.54	4.3475
21	26.05	-0.52	4.3475
22	25.94	-0.54	4.3475
23	25.88	-0.52	4.3475
24	25.77	-0.54	4.3475
25	25.93	-0.52	4.3475
26	25.87	-0.54	4.3475
27	26.09	-0.52	4.3475
28	25.92	-0.54	4.3475
29	25.85	-0.54	4.3475
30	25.84	-0.54	4.3475
31	25.91	-0.54	4.3475

IMC-NP@GCgel

Time	Temp	DTA	TGA
sec	˚ C	uV	mg
0	33.28	-0.51	4.64
1	33.23	-0.51	4.64
2	33.42	-0.51	4.64
3	33.37	-0.51	4.64
4	33.22	-0.52	4.64
5	33.24	-0.52	4.64
6	33.3	-0.52	4.64
7	33.38	-0.52	4.64
8	33.37	-0.52	4.64
9	33.42	-0.52	4.64
10	33.15	-0.53	4.64
11	33.34	-0.53	4.64
12	33.25	-0.53	4.64
13	33.49	-0.53	4.64
14	33.38	-0.53	4.64
15	33.39	-0.53	4.64
16	33.22	-0.54	4.64
17	33.32	-0.54	4.64
18	33.34	-0.54	4.64
19	33.43	-0.54	4.64
20	33.47	-0.54	4.64
21	33.25	-0.54	4.64
22	33.32	-0.55	4.64
23	33.31	-0.55	4.64
24	33.47	-0.55	4.64
25	33.43	-0.55	4.64
26	33.5	-0.55	4.64
27	33.22	-0.55	4.64
28	33.39	-0.56	4.64
29	33.31	-0.56	4.64
30	33.51	-0.56	4.64
31	33.51	-0.56	4.64

32	34.5	-0.67	4.53
33	34.37	-0.69	4.53
34	34.35	-0.69	4.53
35	34.19	-0.69	4.53
36	34.38	-0.69	4.53
37	34.34	-0.67	4.53
38	34.54	-0.69	4.53
39	34.44	-0.69	4.53
40	34.29	-0.69	4.53
41	34.34	-0.69	4.53
42	34.4	-0.69	4.53
43	34.51	-0.69	4.53
44	34.51	-0.69	4.53
45	34.57	-0.69	4.53
46	34.34	-0.69	4.53
47	34.51	-0.69	4.53
48	34.46	-0.69	4.53
49	34.69	-0.69	4.53
50	34.61	-0.69	4.53
51	34.6	-0.69	4.53
52	34.45	-0.69	4.53
53	34.61	-0.69	4.53
54	34.61	-0.67	4.53
55	34.74	-0.69	4.53
56	34.76	-0.67	4.53
57	34.6	-0.69	4.53
58	34.65	-0.69	4.53
59	34.7	-0.69	4.53
60	34.87	-0.67	4.53
61	34.88	-0.67	4.53
62	34.98	-0.67	4.53
63	34.7	-0.67	4.53
64	34.91	-0.67	4.53
65	34.84	-0.67	4.53
66	35.11	-0.67	4.53
67	35.05	-0.66	4.53

32	33.72	-0.39	4.86
33	33.89	-0.40	4.86
34	33.82	-0.40	4.86
35	33.76	-0.40	4.86
36	33.65	-0.40	4.86
37	33.83	-0.40	4.86
38	33.78	-0.40	4.86
39	33.98	-0.40	4.86
40	33.83	-0.41	4.86
41	33.76	-0.41	4.86
42	33.74	-0.41	4.86
43	33.85	-0.41	4.86
44	33.91	-0.41	4.86
45	34.01	-0.41	4.86
46	33.92	-0.41	4.86
47	33.78	-0.41	4.86
48	33.91	-0.41	4.86
49	33.85	-0.41	4.86
50	34.08	-0.41	4.86
51	34.02	-0.41	4.86
52	34.03	-0.41	4.86
53	33.83	-0.41	4.86
54	34.06	-0.41	4.86
55	34	-0.41	4.86
56	34.2	-0.41	4.86
57	34.13	-0.42	4.86
58	34.03	-0.42	4.86
59	34.05	-0.43	4.86
60	34.11	-0.42	4.86
61	34.28	-0.43	4.86
62	34.3	-0.42	4.86
63	34.33	-0.43	4.86
64	34.11	-0.43	4.86
65	34.33	-0.44	4.86
66	34.26	-0.44	4.86
67	34.51	-0.44	4.86

32	25.95	-0.54	4.3475
33	26.04	-0.54	4.3475
34	26.01	-0.54	4.3475
35	25.77	-0.56	4.3475
36	25.92	-0.54	4.3475
37	25.86	-0.54	4.3475
38	26.06	-0.54	4.3475
39	26.02	-0.54	4.3475
40	26.06	-0.54	4.3475
41	25.75	-0.56	4.3475
42	25.98	-0.54	4.3475
43	25.88	-0.54	4.3475
44	26.13	-0.54	4.3475
45	26.06	-0.54	4.3475
46	26.01	-0.54	4.3475
47	25.9	-0.56	4.3475
48	25.97	-0.56	4.3475
49	26.04	-0.54	4.3475
50	26.13	-0.54	4.3475
51	26.19	-0.54	4.3475
52	25.98	-0.54	4.3475
53	26.04	-0.54	4.3475
54	26.01	-0.56	4.3475
55	26.19	-0.54	4.3475
56	26.17	-0.54	4.3475
57	26.29	-0.54	4.3475
58	26.02	-0.56	4.3475
59	26.17	-0.56	4.3475
60	26.12	-0.56	4.3475
61	26.29	-0.56	4.3475
62	26.36	-0.56	4.3475
63	26.36	-0.56	4.3475
64	26.19	-0.56	4.3475
65	26.27	-0.56	4.3475
66	26.39	-0.56	4.3475
67	26.41	-0.56	4.3475

32	33.45	-0.56	4.64
33	33.3	-0.56	4.64
34	33.37	-0.56	4.64
35	33.43	-0.57	4.64
36	33.5	-0.57	4.64
37	33.61	-0.58	4.64
38	33.39	-0.58	4.64
39	33.43	-0.59	4.64
40	33.36	-0.60	4.64
41	33.58	-0.60	4.64
42	33.52	-0.61	4.64
43	33.63	-0.61	4.64
44	33.35	-0.62	4.64
45	33.49	-0.62	4.64
46	33.49	-0.64	4.64
47	33.58	-0.64	4.64
48	33.69	-0.65	4.64
49	33.59	-0.66	4.64
50	33.5	-0.66	4.64
51	33.52	-0.67	4.64
52	33.66	-0.68	4.64
53	33.65	-0.69	4.64
54	33.83	-0.69	4.64
55	33.63	-0.71	4.64
56	33.65	-0.71	4.64
57	33.61	-0.72	4.64
58	33.78	-0.72	4.64
59	33.82	-0.73	4.64
60	33.9	-0.74	4.64
61	33.73	-0.75	4.64
62	33.76	-0.75	4.64
63	33.84	-0.76	4.64
64	33.88	-0.77	4.64
65	34.08	-0.78	4.64
66	33.99	-0.79	4.64
67	33.94	-0.80	4.64

68	35.09	-0.67	4.53
69	34.94	-0.67	4.53
70	35.07	-0.67	4.53
71	35.15	-0.66	4.53
72	35.27	-0.66	4.53
73	35.4	-0.66	4.53
74	35.24	-0.67	4.53
75	35.29	-0.67	4.53
76	35.32	-0.67	4.53
77	35.51	-0.66	4.51
78	35.54	-0.66	4.51
79	35.69	-0.66	4.51
80	35.46	-0.67	4.51
81	35.63	-0.67	4.51
82	35.62	-0.66	4.53
83	35.82	-0.66	4.53
84	35.93	-0.66	4.53
85	35.96	-0.66	4.53
86	35.84	-0.66	4.53
87	35.96	-0.66	4.53
88	36.1	-0.66	4.53
89	36.19	-0.66	4.53
90	36.4	-0.66	4.53
91	36.28	-0.66	4.53
92	36.3	-0.66	4.53
93	36.33	-0.66	4.53
94	36.58	-0.66	4.53
95	36.62	-0.66	4.53
96	36.81	-0.66	4.53
97	36.63	-0.66	4.53
98	36.75	-0.66	4.53
99	36.85	-0.64	4.53
100	37.01	-0.64	4.53
101	37.22	-0.64	4.53
102	37.25	-0.64	4.53
103	37.2	-0.64	4.53

68	34.48	-0.44	4.86
69	34.54	-0.44	4.86
70	34.32	-0.45	4.86
71	34.52	-0.45	4.86
72	34.56	-0.45	4.86
73	34.73	-0.46	4.86
74	34.78	-0.46	4.85
75	34.68	-0.46	4.85
76	34.71	-0.46	4.85
77	34.75	-0.46	4.85
78	34.93	-0.47	4.85
79	35	-0.47	4.85
80	35.11	-0.47	4.85
81	34.87	-0.47	4.85
82	35.08	-0.48	4.85
83	35.07	-0.48	4.85
84	35.33	-0.48	4.85
85	35.35	-0.48	4.85
86	35.46	-0.49	4.86
87	35.28	-0.49	4.86
88	35.43	-0.50	4.86
89	35.55	-0.50	4.86
90	35.7	-0.50	4.86
91	35.84	-0.51	4.86
92	35.75	-0.51	4.86
93	35.78	-0.52	4.86
94	35.83	-0.52	4.85
95	36.03	-0.53	4.85
96	36.12	-0.52	4.85
97	36.35	-0.53	4.85
98	36.14	-0.53	4.85
99	36.29	-0.54	4.85
100	36.33	-0.54	4.85
101	36.55	-0.55	4.85
102	36.66	-0.55	4.85
103	36.77	-0.55	4.85

68	26.6	-0.56	4.3475
69	26.47	-0.56	4.3475
70	26.44	-0.56	4.3475
71	26.4	-0.56	4.3475
72	26.62	-0.56	4.3475
73	26.61	-0.56	4.3475
74	26.82	-0.54	4.3475
75	26.65	-0.56	4.3475
76	26.66	-0.56	4.3475
77	26.71	-0.56	4.3475
78	26.81	-0.56	4.3475
79	26.96	-0.54	4.3475
80	27.01	-0.54	4.3475
81	26.97	-0.54	4.3475
82	26.88	-0.56	4.3475
83	27.06	-0.56	4.3475
84	27.07	-0.56	4.3475
85	27.32	-0.54	4.3475
86	27.3	-0.56	4.3475
87	27.33	-0.56	4.3475
88	27.2	-0.56	4.3475
89	27.45	-0.56	4.3475
90	27.44	-0.56	4.3475
91	27.72	-0.56	4.3475
92	27.67	-0.56	4.3475
93	27.64	-0.56	4.3475
94	27.67	-0.56	4.3475
95	27.8	-0.57	4.3475
96	27.94	-0.56	4.3475
97	28.07	-0.56	4.3475
98	28.14	-0.56	4.3475
99	27.97	-0.56	4.3475
100	28.19	-0.56	4.3475
101	28.21	-0.57	4.3475
102	28.47	-0.56	4.3475
103	28.5	-0.56	4.3475

68	33.88	-0.80	4.64
69	34.11	-0.81	4.64
70	34.07	-0.82	4.64
71	34.3	-0.83	4.64
72	34.16	-0.83	4.64
73	34.15	-0.84	4.64
74	34.18	-0.85	4.64
75	34.26	-0.86	4.64
76	34.45	-0.86	4.64
77	34.5	-0.87	4.64
78	34.44	-0.87	4.64
79	34.35	-0.88	4.64
80	34.55	-0.88	4.64
81	34.54	-0.90	4.64
82	34.8	-0.90	4.64
83	34.73	-0.91	4.64
84	34.76	-0.91	4.64
85	34.67	-0.92	4.64
86	34.92	-0.93	4.64
87	34.91	-0.94	4.64
88	35.15	-0.94	4.64
89	35.11	-0.95	4.64
90	35.05	-0.95	4.64
91	35.17	-0.95	4.64
92	35.24	-0.96	4.64
93	35.44	-0.97	4.64
94	35.51	-0.97	4.64
95	35.59	-0.98	4.64
96	35.39	-0.98	4.64
97	35.66	-0.99	4.64
98	35.65	-1.00	4.64
99	35.93	-1.00	4.64
100	35.92	-1.01	4.64
101	36.02	-1.01	4.64
102	35.91	-1.02	4.64
103	36.16	-1.02	4.64

104	37.23	-0.64	4.53
105	37.47	-0.64	4.53
106	37.51	-0.64	4.53
107	37.79	-0.62	4.53
108	37.72	-0.64	4.53
109	37.73	-0.64	4.53
110	37.76	-0.62	4.53
111	38.01	-0.64	4.53
112	38.13	-0.62	4.53
113	38.34	-0.62	4.53
114	38.32	-0.62	4.53
115	38.32	-0.62	4.53
116	38.5	-0.62	4.53
117	38.59	-0.62	4.53
118	38.84	-0.62	4.53
119	38.92	-0.62	4.53
120	38.95	-0.64	4.51
121	38.89	-0.64	4.51
122	39.21	-0.64	4.51
123	39.2	-0.64	4.51
124	39.55	-0.64	4.51
125	39.52	-0.64	4.51
126	39.59	-0.66	4.51
127	39.63	-0.64	4.51
128	39.86	-0.66	4.51
129	40	-0.64	4.51
130	40.21	-0.66	4.51
131	40.28	-0.64	4.51
132	40.2	-0.66	4.51
133	40.43	-0.64	4.51
134	40.5	-0.66	4.51
135	40.76	-0.64	4.50
136	40.87	-0.66	4.50
137	41.01	-0.66	4.50
138	40.86	-0.66	4.50
139	41.19	-0.66	4.50

104	36.69	-0.55	4.85
105	36.77	-0.56	4.85
106	36.94	-0.56	4.85
107	37.04	-0.57	4.85
108	37.27	-0.57	4.85
109	37.22	-0.57	4.85
110	37.27	-0.57	4.85
111	37.31	-0.58	4.85
112	37.56	-0.59	4.85
113	37.63	-0.59	4.85
114	37.91	-0.59	4.85
115	37.82	-0.59	4.85
116	37.83	-0.60	4.85
117	38.01	-0.60	4.85
118	38.15	-0.61	4.85
119	38.38	-0.61	4.84
120	38.46	-0.62	4.84
121	38.5	-0.62	4.84
122	38.47	-0.62	4.84
123	38.73	-0.62	4.84
124	38.78	-0.64	4.84
125	39.12	-0.64	4.84
126	39.08	-0.64	4.84
127	39.16	-0.65	4.84
128	39.18	-0.65	4.84
129	39.43	-0.66	4.84
130	39.53	-0.66	4.84
131	39.79	-0.67	4.84
132	39.83	-0.67	4.84
133	39.8	-0.68	4.84
134	39.97	-0.68	4.84
135	40.13	-0.69	4.84
136	40.36	-0.69	4.84
137	40.48	-0.70	4.84
138	40.64	-0.70	4.84
139	40.49	-0.70	4.84

104	28.66	-0.56	4.3475
105	28.43	-0.57	4.3475
106	28.69	-0.57	4.3475
107	28.72	-0.57	4.3475
108	28.96	-0.57	4.3475
109	29.05	-0.57	4.3475
110	29.08	-0.57	4.3475
111	29.01	-0.57	4.3475
112	29.13	-0.59	4.3475
113	29.3	-0.57	4.3475
114	29.42	-0.59	4.3475
115	29.66	-0.57	4.3475
116	29.55	-0.57	4.3475
117	29.61	-0.59	4.3475
118	29.66	-0.59	4.3475
119	29.91	-0.59	4.3475
120	29.98	-0.59	4.3475
121	30.23	-0.59	4.3475
122	30.07	-0.59	4.3475
123	30.19	-0.61	4.3475
124	30.27	-0.61	4.3475
125	30.47	-0.61	4.3475
126	30.65	-0.61	4.3475
127	30.76	-0.61	4.3475
128	30.74	-0.61	4.3475
129	30.78	-0.63	4.3475
130	30.99	-0.63	4.3475
131	31.09	-0.63	4.3475
132	31.41	-0.63	4.3475
133	31.36	-0.63	4.3475
134	31.43	-0.63	4.3475
135	31.43	-0.65	4.3475
136	31.71	-0.65	4.3475
137	31.75	-0.65	4.3475
138	32.05	-0.65	4.3475
139	32.08	-0.65	4.3475

104	36.22	-1.03	4.64
105	36.45	-1.03	4.64
106	36.52	-1.04	4.64
107	36.44	-1.05	4.64
108	36.57	-1.05	4.64
109	36.64	-1.05	4.64
110	36.85	-1.06	4.64
111	36.96	-1.06	4.64
112	37.15	-1.07	4.64
113	36.92	-1.08	4.64
114	37.19	-1.08	4.64
115	37.21	-1.09	4.64
116	37.49	-1.09	4.64
117	37.57	-1.10	4.64
118	37.71	-1.10	4.64
119	37.58	-1.11	4.64
120	37.8	-1.11	4.64
121	37.93	-1.12	4.64
122	38.12	-1.13	4.64
123	38.32	-1.13	4.64
124	38.25	-1.13	4.64
125	38.32	-1.14	4.64
126	38.42	-1.14	4.64
127	38.66	-1.15	4.64
128	38.78	-1.16	4.64
129	39.01	-1.16	4.64
130	38.88	-1.17	4.64
131	39.06	-1.17	4.64
132	39.15	-1.18	4.64
133	39.38	-1.19	4.64
134	39.55	-1.19	4.64
135	39.67	-1.20	4.64
136	39.65	-1.20	4.64
137	39.75	-1.20	4.64
138	39.96	-1.21	4.64
139	40.08	-1.21	4.64

140	41.25	-0.66	4.50
141	41.59	-0.66	4.50
142	41.62	-0.64	4.50
143	41.74	-0.66	4.51
144	41.73	-0.64	4.51
145	41.92	-0.66	4.51
146	42.08	-0.64	4.51
147	42.32	-0.64	4.51
148	42.49	-0.64	4.51
149	42.4	-0.66	4.51
150	42.63	-0.64	4.51
151	42.72	-0.66	4.51
152	42.99	-0.64	4.51
153	43.12	-0.64	4.51
154	43.36	-0.64	4.50
155	43.21	-0.66	4.50
156	43.5	-0.66	4.50
157	43.59	-0.66	4.50
158	43.86	-0.66	4.50
159	44.01	-0.66	4.50
160	44.13	-0.66	4.50
161	44.1	-0.66	4.50
162	44.29	-0.66	4.50
163	44.5	-0.66	4.50
164	44.7	-0.66	4.50
165	44.97	-0.66	4.50
166	44.91	-0.67	4.50
167	45.07	-0.67	4.50
168	45.17	-0.67	4.50
169	45.48	-0.67	4.50
170	45.61	-0.67	4.50
171	45.9	-0.67	4.50
172	45.81	-0.69	4.50
173	46	-0.69	4.50
174	46.17	-0.69	4.50
175	46.39	-0.69	4.50

140	40.79	-0.70	4.84
141	40.83	-0.71	4.84
142	41.17	-0.71	4.84
143	41.2	-0.71	4.84
144	41.34	-0.72	4.84
145	41.3	-0.72	4.84
146	41.56	-0.72	4.84
147	41.68	-0.72	4.84
148	41.92	-0.74	4.84
149	42.07	-0.73	4.84
150	42.02	-0.74	4.84
151	42.19	-0.74	4.84
152	42.31	-0.75	4.84
153	42.59	-0.75	4.84
154	42.7	-0.76	4.84
155	42.91	-0.76	4.84
156	42.78	-0.76	4.84
157	43.07	-0.76	4.84
158	43.13	-0.77	4.84
159	43.44	-0.77	4.84
160	43.55	-0.78	4.84
161	43.69	-0.78	4.84
162	43.64	-0.79	4.83
163	43.89	-0.79	4.83
164	44.07	-0.79	4.83
165	44.28	-0.80	4.83
166	44.52	-0.81	4.83
167	44.48	-0.81	4.83
168	44.59	-0.81	4.83
169	44.72	-0.81	4.83
170	45.03	-0.81	4.83
171	45.17	-0.82	4.83
172	45.44	-0.83	4.83
173	45.35	-0.83	4.83
174	45.55	-0.83	4.83
175	45.65	-0.84	4.83

140	32.09	-0.67	4.3475
141	32.2	-0.67	4.3475
142	32.41	-0.67	4.3475
143	32.61	-0.67	4.3475
144	32.77	-0.67	4.3475
145	32.87	-0.67	4.3475
146	32.79	-0.68	4.3475
147	33.06	-0.68	4.366
148	33.13	-0.68	4.366
149	33.44	-0.68	4.366
150	33.5	-0.68	4.366
151	33.69	-0.68	4.366
152	33.52	-0.70	4.366
153	33.86	-0.70	4.366
154	33.93	-0.70	4.366
155	34.25	-0.70	4.366
156	34.34	-0.70	4.366
157	34.45	-0.70	4.366
158	34.41	-0.72	4.366
159	34.63	-0.74	4.366
160	34.83	-0.74	4.3475
161	35.04	-0.74	4.3475
162	35.24	-0.74	4.366
163	35.19	-0.76	4.366
164	35.33	-0.76	4.366
165	35.42	-0.78	4.366
166	35.72	-0.78	4.366
167	35.85	-0.78	4.366
168	36.14	-0.78	4.366
169	36.03	-0.80	4.366
170	36.24	-0.80	4.366
171	36.35	-0.81	4.366
172	36.6	-0.81	4.3475
173	36.81	-0.81	4.3475
174	36.95	-0.83	4.3475
175	36.93	-0.83	4.3475

140	40.37	-1.22	4.64
141	40.34	-1.23	4.64
142	40.4	-1.23	4.64
143	40.48	-1.23	4.64
144	40.77	-1.24	4.64
145	40.87	-1.25	4.64
146	41.16	-1.25	4.64
147	41.15	-1.25	4.64
148	41.18	-1.25	4.64
149	41.36	-1.26	4.64
150	41.54	-1.27	4.64
151	41.79	-1.27	4.64
152	41.9	-1.28	4.64
153	41.98	-1.28	4.64
154	41.98	-1.28	4.64
155	42.25	-1.29	4.64
156	42.32	-1.29	4.64
157	42.69	-1.30	4.64
158	42.69	-1.30	4.64
159	42.81	-1.31	4.64
160	42.8	-1.31	4.64
161	43.13	-1.31	4.64
162	43.21	-1.32	4.64
163	43.51	-1.32	4.64
164	43.61	-1.33	4.64
165	43.59	-1.33	4.64
166	43.77	-1.34	4.64
167	43.92	-1.34	4.64
168	44.19	-1.35	4.64
169	44.34	-1.35	4.64
170	44.53	-1.35	4.64
171	44.43	-1.35	4.64
172	44.73	-1.36	4.64
173	44.8	-1.36	4.64
174	45.16	-1.36	4.64
175	45.25	-1.37	4.64

176	46.63	-0.69	4.50
177	46.77	-0.70	4.50
178	46.82	-0.70	4.50
179	46.94	-0.70	4.51
180	47.21	-0.70	4.51
181	47.38	-0.70	4.51
182	47.7	-0.70	4.51
183	47.71	-0.70	4.51
184	47.81	-0.70	4.51
185	47.93	-0.72	4.51
186	48.22	-0.72	4.51
187	48.35	-0.70	4.51
188	48.67	-0.72	4.51
189	48.68	-0.72	4.51
190	48.72	-0.74	4.51
191	48.93	-0.72	4.51
192	49.14	-0.74	4.51
193	49.39	-0.74	4.51
194	49.54	-0.74	4.51
195	49.68	-0.75	4.51
196	49.65	-0.75	4.51
197	49.96	-0.77	4.51
198	50.05	-0.77	4.51
199	50.44	-0.77	4.51
200	50.45	-0.78	4.51
201	50.57	-0.80	4.51
202	50.62	-0.80	4.51
203	50.9	-0.80	4.51
204	51.03	-0.80	4.51
205	51.34	-0.82	4.51
206	51.46	-0.82	4.51
207	51.46	-0.83	4.51
208	51.65	-0.83	4.51
209	51.83	-0.83	4.51
210	52.08	-0.83	4.51
211	52.26	-0.85	4.51

176	45.93	-0.84	4.83
177	46.14	-0.85	4.83
178	46.3	-0.85	4.83
179	46.29	-0.86	4.83
180	46.44	-0.86	4.83
181	46.69	-0.86	4.83
182	46.82	-0.86	4.83
183	47.14	-0.87	4.83
184	47.18	-0.88	4.83
185	47.25	-0.88	4.83
186	47.34	-0.88	4.83
187	47.71	-0.88	4.83
188	47.8	-0.89	4.83
189	48.1	-0.89	4.83
190	48.14	-0.90	4.83
191	48.23	-0.90	4.83
192	48.42	-0.91	4.82
193	48.62	-0.91	4.82
194	48.88	-0.91	4.82
195	49.05	-0.92	4.82
196	49.14	-0.93	4.82
197	49.15	-0.93	4.82
198	49.48	-0.94	4.82
199	49.56	-0.94	4.82
200	49.92	-0.94	4.82
201	49.97	-0.95	4.82
202	50.11	-0.95	4.82
203	50.12	-0.96	4.82
204	50.46	-0.96	4.82
205	50.59	-0.96	4.82
206	50.91	-0.97	4.82
207	51.01	-0.97	4.82
208	51.06	-0.98	4.82
209	51.22	-0.98	4.81
210	51.41	-0.98	4.81
211	51.68	-0.99	4.81

176	37.07	-0.85	4.3475
177	37.29	-0.85	4.3475
178	37.45	-0.87	4.3475
179	37.77	-0.87	4.3475
180	37.81	-0.87	4.3475
181	37.89	-0.87	4.3475
182	37.98	-0.89	4.3475
183	38.29	-0.89	4.3475
184	38.36	-0.91	4.3475
185	38.75	-0.91	4.3475
186	38.75	-0.91	4.3475
187	38.83	-0.93	4.3475
188	38.96	-0.93	4.3475
189	39.22	-0.93	4.366
190	39.38	-0.94	4.366
191	39.61	-0.94	4.366
192	39.75	-0.94	4.366
193	39.69	-0.96	4.366
194	39.96	-0.96	4.366
195	40.1	-0.96	4.366
196	40.42	-0.96	4.366
197	40.54	-0.98	4.366
198	40.74	-0.98	4.366
199	40.63	-1.00	4.366
200	41	-1.00	4.366
201	41.05	-1.00	4.366
202	41.4	-1.00	4.366
203	41.53	-1.02	4.366
204	41.68	-1.02	4.366
205	41.64	-1.04	4.366
206	41.92	-1.04	4.366
207	42.11	-1.04	4.366
208	42.36	-1.04	4.366
209	42.58	-1.04	4.366
210	42.62	-1.05	4.366
211	42.74	-1.07	4.366

176	45.41	-1.37	4.64
177	45.33	-1.38	4.64
178	45.62	-1.38	4.64
179	45.78	-1.38	4.64
180	46.05	-1.39	4.64
181	46.21	-1.39	4.64
182	46.24	-1.39	4.64
183	46.34	-1.39	4.64
184	46.51	-1.39	4.64
185	46.81	-1.40	4.64
186	46.95	-1.40	4.64
187	47.22	-1.40	4.64
188	47.13	-1.41	4.64
189	47.38	-1.41	4.64
190	47.46	-1.42	4.64
191	47.78	-1.42	4.64
192	47.94	-1.43	4.64
193	48.14	-1.43	4.64
194	48.08	-1.43	4.64
195	48.32	-1.43	4.64
196	48.52	-1.44	4.64
197	48.72	-1.44	4.64
198	48.97	-1.45	4.64
199	49.07	-1.45	4.64
200	49.11	-1.46	4.64
201	49.25	-1.46	4.64
202	49.57	-1.46	4.64
203	49.67	-1.46	4.64
204	50.01	-1.47	4.64
205	49.98	-1.47	4.64
206	50.1	-1.47	4.64
207	50.22	-1.48	4.64
208	50.5	-1.48	4.64
209	50.69	-1.49	4.64
210	50.96	-1.49	4.64
211	51.01	-1.49	4.64

212	52.46	-0.85	4.51
213	52.36	-0.86	4.51
214	52.67	-0.86	4.51
215	52.75	-0.88	4.51
216	53.13	-0.88	4.51
217	53.2	-0.90	4.51
218	53.42	-0.91	4.51
219	53.36	-0.91	4.51
220	53.67	-0.91	4.51
221	53.85	-0.91	4.51
222	54.1	-0.93	4.51
223	54.3	-0.93	4.51
224	54.31	-0.94	4.51
225	54.46	-0.94	4.51
226	54.62	-0.96	4.51
227	54.92	-0.96	4.51
228	55.09	-0.98	4.51
229	55.34	-0.98	4.53
230	55.24	-0.99	4.53
231	55.53	-0.99	4.53
232	55.63	-0.99	4.51
233	55.98	-1.01	4.51
234	56.15	-1.01	4.51
235	56.36	-1.01	4.51
236	56.3	-1.01	4.51
237	56.55	-1.02	4.51
238	56.76	-1.02	4.51
239	56.98	-1.02	4.51
240	57.25	-1.02	4.51
241	57.34	-1.04	4.51
242	57.4	-1.06	4.51
243	57.53	-1.06	4.51
244	57.85	-1.06	4.51
245	58	-1.07	4.51
246	58.32	-1.07	4.51
247	58.31	-1.09	4.51

212	51.86	-0.99	4.81
213	52.09	-1.00	4.81
214	51.97	-1.00	4.81
215	52.32	-1.01	4.81
216	52.41	-1.01	4.81
217	52.78	-1.01	4.81
218	52.89	-1.02	4.81
219	53.1	-1.02	4.81
220	53.02	-1.03	4.80
221	53.33	-1.03	4.80
222	53.51	-1.03	4.80
223	53.78	-1.03	4.80
224	53.99	-1.04	4.80
225	54.05	-1.04	4.80
226	54.16	-1.05	4.80
227	54.34	-1.05	4.80
228	54.61	-1.05	4.80
229	54.83	-1.05	4.79
230	55.08	-1.06	4.79
231	55.02	-1.06	4.79
232	55.25	-1.07	4.79
233	55.4	-1.07	4.79
234	55.7	-1.08	4.79
235	55.86	-1.08	4.79
236	56.13	-1.08	4.79
237	56.08	-1.09	4.79
238	56.3	-1.09	4.79
239	56.52	-1.10	4.79
240	56.74	-1.10	4.79
241	57.02	-1.10	4.79
242	57.11	-1.10	4.78
243	57.21	-1.11	4.78
244	57.33	-1.11	4.78
245	57.65	-1.12	4.78
246	57.8	-1.12	4.78
247	58.14	-1.12	4.78

212	42.92	-1.07	4.366
213	43.18	-1.07	4.366
214	43.35	-1.07	4.366
215	43.64	-1.07	4.366
216	43.55	-1.09	4.366
217	43.79	-1.09	4.366
218	43.89	-1.11	4.366
219	44.18	-1.11	4.366
220	44.36	-1.11	4.366
221	44.61	-1.13	4.366
222	44.59	-1.13	4.366
223	44.75	-1.15	4.366
224	44.98	-1.15	4.366
225	45.15	-1.17	4.366
226	45.46	-1.15	4.366
227	45.56	-1.17	4.366
228	45.68	-1.17	4.366
229	45.73	-1.18	4.366
230	46.03	-1.18	4.366
231	46.16	-1.20	4.3475
232	46.49	-1.20	4.3475
233	46.52	-1.20	4.3475
234	46.67	-1.20	4.3475
235	46.74	-1.22	4.3475
236	47.05	-1.22	4.3475
237	47.2	-1.22	4.3475
238	47.52	-1.24	4.3475
239	47.61	-1.24	4.3475
240	47.63	-1.26	4.3475
241	47.85	-1.26	4.3475
242	48.06	-1.28	4.3475
243	48.32	-1.28	4.3475
244	48.5	-1.28	4.3475
245	48.71	-1.28	4.3475
246	48.59	-1.30	4.3475
247	48.94	-1.30	4.3475

212	51.06	-1.49	4.64
213	51.32	-1.50	4.64
214	51.46	-1.50	4.64
215	51.79	-1.51	4.64
216	51.9	-1.51	4.64
217	52.03	-1.51	4.64
218	52.03	-1.51	4.64
219	52.37	-1.52	4.64
220	52.46	-1.52	4.64
221	52.83	-1.53	4.64
222	52.86	-1.53	4.64
223	52.99	-1.53	4.64
224	53.09	-1.53	4.64
225	53.36	-1.53	4.64
226	53.54	-1.54	4.64
227	53.83	-1.54	4.64
228	53.97	-1.54	4.64
229	53.93	-1.54	4.64
230	54.21	-1.55	4.64
231	54.34	-1.55	4.64
232	54.68	-1.56	4.64
233	54.79	-1.56	4.64
234	55.04	-1.56	4.64
235	54.9	-1.56	4.64
236	55.25	-1.56	4.64
237	55.35	-1.57	4.64
238	55.71	-1.57	4.64
239	55.86	-1.57	4.64
240	56.01	-1.58	4.64
241	55.99	-1.58	4.64
242	56.24	-1.58	4.64
243	56.45	-1.58	4.64
244	56.7	-1.59	4.64
245	56.96	-1.60	4.64
246	56.94	-1.60	4.64
247	57.08	-1.60	4.64

248	58.49	-1.09	4.51
249	58.63	-1.09	4.51
250	58.89	-1.10	4.51
251	59.12	-1.10	4.51
252	59.32	-1.12	4.51
253	59.35	-1.12	4.51
254	59.47	-1.14	4.51
255	59.74	-1.14	4.51
256	59.88	-1.14	4.51
257	60.21	-1.15	4.51
258	60.3	-1.15	4.51
259	60.42	-1.17	4.51
260	60.49	-1.17	4.51
261	60.84	-1.18	4.51
262	60.94	-1.18	4.51
263	61.3	-1.18	4.51
264	61.33	-1.20	4.51
265	61.44	-1.22	4.51
266	61.59	-1.22	4.51
267	61.84	-1.23	4.51
268	62.06	-1.22	4.51
269	62.32	-1.23	4.51
270	62.43	-1.23	4.51
271	62.41	-1.25	4.51
272	62.71	-1.25	4.51
273	62.86	-1.26	4.51
274	63.2	-1.26	4.51
275	63.31	-1.26	4.51
276	63.52	-1.28	4.51
277	63.46	-1.30	4.51
278	63.82	-1.30	4.51
279	63.92	-1.30	4.51
280	64.29	-1.31	4.51
281	64.4	-1.31	4.51
282	64.5	-1.33	4.51
283	64.62	-1.33	4.51

248	58.16	-1.12	4.78
249	58.27	-1.13	4.78
250	58.44	-1.13	4.78
251	58.71	-1.14	4.78
252	58.91	-1.14	4.78
253	59.16	-1.14	4.78
254	59.24	-1.15	4.78
255	59.28	-1.15	4.77
256	59.54	-1.16	4.77
257	59.72	-1.16	4.77
258	60.02	-1.17	4.77
259	60.16	-1.17	4.77
260	60.31	-1.18	4.77
261	60.32	-1.18	4.77
262	60.66	-1.18	4.76
263	60.73	-1.18	4.76
264	61.14	-1.19	4.76
265	61.17	-1.19	4.76
266	61.32	-1.19	4.76
267	61.39	-1.20	4.76
268	61.71	-1.20	4.76
269	61.87	-1.20	4.76
270	62.13	-1.21	4.76
271	62.33	-1.21	4.76
272	62.31	-1.22	4.76
273	62.54	-1.22	4.75
274	62.68	-1.22	4.75
275	63.01	-1.23	4.75
276	63.16	-1.23	4.75
277	63.41	-1.24	4.75
278	63.32	-1.24	4.75
279	63.67	-1.25	4.75
280	63.75	-1.25	4.74
281	64.11	-1.25	4.74
282	64.22	-1.25	4.74
283	64.41	-1.26	4.74

248	49.03	-1.31	4.3475
249	49.4	-1.31	4.3475
250	49.49	-1.31	4.3475
251	49.72	-1.31	4.3475
252	49.63	-1.33	4.3475
253	49.96	-1.33	4.3475
254	50.08	-1.33	4.3475
255	50.39	-1.35	4.3475
256	50.59	-1.35	4.3475
257	50.7	-1.35	4.3475
258	50.74	-1.37	4.3475
259	50.94	-1.37	4.3475
260	51.21	-1.37	4.3475
261	51.41	-1.39	4.3475
262	51.68	-1.37	4.3475
263	51.68	-1.39	4.3475
264	51.84	-1.39	4.3475
265	51.96	-1.41	4.3475
266	52.29	-1.41	4.3475
267	52.45	-1.41	4.3475
268	52.74	-1.41	4.3475
269	52.7	-1.42	4.3475
270	52.88	-1.42	4.3475
271	53.05	-1.44	4.3475
272	53.28	-1.44	4.3475
273	53.53	-1.44	4.3475
274	53.72	-1.46	4.3475
275	53.8	-1.46	4.3475
276	53.85	-1.48	4.3475
277	54.17	-1.48	4.3475
278	54.26	-1.48	4.3475
279	54.64	-1.48	4.3475
280	54.7	-1.50	4.3475
281	54.87	-1.50	4.3475
282	54.88	-1.52	4.3475
283	55.23	-1.52	4.3475

248	57.23	-1.60	4.64
249	57.54	-1.61	4.64
250	57.69	-1.61	4.64
251	58	-1.61	4.64
252	57.93	-1.62	4.64
253	58.17	-1.62	4.64
254	58.3	-1.62	4.64
255	58.61	-1.62	4.64
256	58.81	-1.63	4.64
257	58.97	-1.63	4.64
258	59.01	-1.64	4.64
259	59.14	-1.64	4.64
260	59.4	-1.64	4.64
261	59.57	-1.64	4.64
262	59.89	-1.65	4.64
263	59.96	-1.65	4.64
264	60.07	-1.65	4.64
265	60.17	-1.65	4.64
266	60.52	-1.65	4.64
267	60.63	-1.66	4.64
268	60.97	-1.67	4.64
269	61.02	-1.67	4.64
270	61.07	-1.67	4.64
271	61.26	-1.67	4.64
272	61.48	-1.67	4.64
273	61.75	-1.68	4.64
274	61.97	-1.68	4.64
275	62.06	-1.68	4.64
276	62.08	-1.68	4.64
277	62.38	-1.69	4.64
278	62.49	-1.69	4.64
279	62.86	-1.69	4.64
280	62.98	-1.69	4.64
281	63.17	-1.69	4.64
282	63.11	-1.69	4.64
283	63.48	-1.69	4.64

284	64.85	-1.34	4.51
285	65.06	-1.34	4.51
286	65.31	-1.36	4.51
287	65.51	-1.36	4.51
288	65.49	-1.38	4.51
289	65.75	-1.38	4.51
290	65.89	-1.38	4.51
291	66.21	-1.39	4.51
292	66.35	-1.39	4.51
293	66.61	-1.41	4.51
294	66.51	-1.41	4.51
295	66.82	-1.42	4.51
296	66.94	-1.42	4.51
297	67.29	-1.44	4.51
298	67.46	-1.44	4.51
299	67.6	-1.47	4.51
300	67.61	-1.47	4.51
301	67.86	-1.49	4.51
302	68.1	-1.49	4.51
303	68.3	-1.50	4.51
304	68.59	-1.52	4.51
305	68.6	-1.54	4.51
306	68.74	-1.54	4.51
307	68.9	-1.54	4.51
308	69.19	-1.55	4.51
309	69.34	-1.55	4.51
310	69.65	-1.57	4.51
311	69.62	-1.58	4.51
312	69.81	-1.58	4.51
313	69.98	-1.58	4.51
314	70.23	-1.58	4.51
315	70.52	-1.58	4.51
316	70.65	-1.60	4.51
317	70.71	-1.62	4.51
318	70.85	-1.62	4.51
319	71.12	-1.63	4.51

284	64.4	-1.26	4.74
285	64.67	-1.27	4.74
286	64.87	-1.27	4.74
287	65.11	-1.27	4.74
288	65.34	-1.28	4.74
289	65.4	-1.28	4.74
290	65.54	-1.29	4.74
291	65.67	-1.29	4.74
292	65.99	-1.30	4.74
293	66.15	-1.30	4.74
294	66.44	-1.31	4.73
295	66.37	-1.30	4.73
296	66.62	-1.31	4.73
297	66.74	-1.32	4.73
298	67.03	-1.32	4.73
299	67.24	-1.32	4.73
300	67.43	-1.33	4.73
301	67.45	-1.33	4.72
302	67.62	-1.34	4.72
303	67.88	-1.34	4.72
304	68.04	-1.35	4.72
305	68.37	-1.35	4.72
306	68.43	-1.35	4.72
307	68.56	-1.35	4.72
308	68.64	-1.36	4.72
309	68.97	-1.36	4.72
310	69.08	-1.36	4.72
311	69.44	-1.37	4.72
312	69.45	-1.37	4.71
313	69.55	-1.37	4.71
314	69.73	-1.38	4.71
315	69.97	-1.39	4.71
316	70.21	-1.39	4.71
317	70.44	-1.39	4.71
318	70.55	-1.39	4.71
319	70.54	-1.40	4.71

284	55.33	-1.52	4.3475
285	55.68	-1.52	4.3475
286	55.76	-1.52	4.3475
287	55.87	-1.52	4.3475
288	56.01	-1.54	4.3475
289	56.25	-1.55	4.3475
290	56.46	-1.55	4.3475
291	56.73	-1.55	4.3475
292	56.91	-1.55	4.3475
293	56.88	-1.57	4.3475
294	57.16	-1.57	4.3475
295	57.31	-1.57	4.3475
296	57.61	-1.57	4.3475
297	57.74	-1.59	4.3475
298	58.02	-1.59	4.3475
299	57.91	-1.61	4.3475
300	58.23	-1.61	4.3475
301	58.35	-1.61	4.3475
302	58.68	-1.63	4.3475
303	58.84	-1.63	4.3475
304	59.03	-1.63	4.3475
305	59	-1.65	4.3475
306	59.25	-1.65	4.3475
307	59.48	-1.65	4.3475
308	59.67	-1.67	4.3475
309	59.97	-1.65	4.3475
310	60	-1.67	4.3475
311	60.09	-1.67	4.3475
312	60.25	-1.68	4.3475
313	60.57	-1.68	4.3475
314	60.72	-1.68	4.3475
315	61.03	-1.68	4.3475
316	61.03	-1.70	4.3475
317	61.18	-1.70	4.3475
318	61.3	-1.70	4.3475
319	61.59	-1.72	4.3475

284	63.58	-1.70	4.64
285	63.94	-1.71	4.64
286	64.03	-1.71	4.64
287	64.16	-1.71	4.64
288	64.23	-1.71	4.64
289	64.48	-1.71	4.64
290	64.7	-1.71	4.64
291	64.96	-1.72	4.64
292	65.17	-1.72	4.64
293	65.14	-1.72	4.64
294	65.41	-1.72	4.64
295	65.52	-1.72	4.64
296	65.84	-1.72	4.64
297	65.98	-1.73	4.64
298	66.26	-1.73	4.64
299	66.13	-1.73	4.64
300	66.42	-1.73	4.64
301	66.58	-1.73	4.64
302	66.86	-1.73	4.64
303	67.06	-1.74	4.64
304	67.24	-1.73	4.64
305	67.25	-1.74	4.64
306	67.45	-1.74	4.64
307	67.71	-1.74	4.64
308	67.91	-1.74	4.64
309	68.2	-1.75	4.64
310	68.23	-1.75	4.64
311	68.34	-1.75	4.64
312	68.51	-1.75	4.64
313	68.81	-1.75	4.64
314	68.94	-1.75	4.64
315	69.27	-1.75	4.64
316	69.27	-1.75	4.64
317	69.4	-1.75	4.64
318	69.6	-1.75	4.64
319	69.86	-1.75	4.64

320	71.27	-1.63	4.51
321	71.62	-1.63	4.51
322	71.65	-1.65	4.51
323	71.76	-1.66	4.51
324	71.88	-1.66	4.51
325	72.2	-1.66	4.51
326	72.34	-1.66	4.51
327	72.67	-1.68	4.51
328	72.76	-1.68	4.51
329	72.8	-1.70	4.51
330	73.02	-1.70	4.51
331	73.25	-1.71	4.51
332	73.5	-1.71	4.51
333	73.69	-1.73	4.51
334	73.82	-1.73	4.51
335	73.84	-1.74	4.51
336	74.15	-1.74	4.51
337	74.26	-1.74	4.51
338	74.67	-1.74	4.51
339	74.7	-1.76	4.51
340	74.9	-1.78	4.51
341	74.91	-1.78	4.51
342	75.28	-1.78	4.51
343	75.39	-1.78	4.51
344	75.72	-1.78	4.51
345	75.86	-1.78	4.51
346	75.93	-1.79	4.51
347	76.1	-1.79	4.51
348	76.29	-1.79	4.51
349	76.59	-1.79	4.51
350	76.78	-1.81	4.51
351	77	-1.81	4.51
352	76.97	-1.82	4.51
353	77.27	-1.82	4.50
354	77.38	-1.82	4.50
355	77.75	-1.84	4.50

320	70.85	-1.40	4.71
321	71	-1.41	4.71
322	71.33	-1.41	4.71
323	71.42	-1.41	4.71
324	71.64	-1.41	4.71
325	71.6	-1.42	4.71
326	71.95	-1.42	4.70
327	72.04	-1.43	4.70
328	72.42	-1.43	4.70
329	72.52	-1.43	4.70
330	72.62	-1.43	4.70
331	72.76	-1.43	4.70
332	72.99	-1.44	4.70
333	73.21	-1.44	4.70
334	73.45	-1.45	4.70
335	73.67	-1.45	4.69
336	73.63	-1.45	4.69
337	73.89	-1.45	4.69
338	74.02	-1.46	4.69
339	74.36	-1.47	4.69
340	74.49	-1.47	4.69
341	74.76	-1.47	4.69
342	74.67	-1.47	4.69
343	74.99	-1.47	4.69
344	75.14	-1.48	4.69
345	75.44	-1.48	4.68
346	75.64	-1.48	4.68
347	75.79	-1.49	4.68
348	75.82	-1.48	4.68
349	76.06	-1.49	4.68
350	76.3	-1.49	4.68
351	76.52	-1.50	4.67
352	76.83	-1.50	4.67
353	76.82	-1.51	4.67
354	76.98	-1.50	4.67
355	77.12	-1.51	4.67

320	61.82	-1.72	4.3475
321	62.05	-1.72	4.3475
322	62.11	-1.74	4.3475
323	62.19	-1.76	4.3475
324	62.42	-1.74	4.3475
325	62.61	-1.76	4.3475
326	62.92	-1.76	4.3475
327	63.05	-1.76	4.3475
328	63.2	-1.76	4.3475
329	63.18	-1.78	4.3475
330	63.54	-1.78	4.3475
331	63.62	-1.78	4.3475
332	64	-1.78	4.3475
333	64.07	-1.79	4.3475
334	64.25	-1.79	4.3475
335	64.28	-1.81	4.3475
336	64.55	-1.81	4.366
337	64.74	-1.83	4.366
338	65.01	-1.83	4.3475
339	65.18	-1.83	4.3475
340	65.22	-1.85	4.3475
341	65.39	-1.85	4.3475
342	65.59	-1.87	4.3475
343	65.84	-1.87	4.3475
344	66.06	-1.87	4.3475
345	66.3	-1.87	4.3475
346	66.23	-1.89	4.3475
347	66.49	-1.89	4.3475
348	66.63	-1.89	4.3475
349	66.94	-1.89	4.3475
350	67.08	-1.91	4.3475
351	67.35	-1.91	4.3475
352	67.28	-1.91	4.3475
353	67.55	-1.92	4.3475
354	67.72	-1.92	4.3475
355	67.99	-1.92	4.3475

320	70.1	-1.76	4.64
321	70.31	-1.76	4.64
322	70.4	-1.76	4.64
323	70.45	-1.76	4.64
324	70.73	-1.76	4.64
325	70.87	-1.76	4.64
326	71.23	-1.76	4.64
327	71.3	-1.76	4.64
328	71.48	-1.77	4.64
329	71.5	-1.77	4.64
330	71.84	-1.77	4.64
331	71.96	-1.77	4.64
332	72.32	-1.77	4.64
333	72.44	-1.77	4.64
334	72.52	-1.77	4.64
335	72.64	-1.77	4.64
336	72.9	-1.77	4.64
337	73.13	-1.78	4.64
338	73.35	-1.78	4.64
339	73.55	-1.78	4.64
340	73.53	-1.78	4.64
341	73.8	-1.78	4.64
342	73.92	-1.78	4.64
343	74.25	-1.79	4.64
344	74.4	-1.79	4.64
345	74.66	-1.79	4.64
346	74.56	-1.79	4.64
347	74.93	-1.79	4.64
348	75.03	-1.80	4.64
349	75.35	-1.80	4.64
350	75.54	-1.80	4.64
351	75.7	-1.80	4.64
352	75.7	-1.80	4.64
353	75.94	-1.80	4.64
354	76.17	-1.80	4.64
355	76.41	-1.80	4.64

356	77.89	-1.84	4.50
357	78.14	-1.86	4.50
358	78.05	-1.86	4.50
359	78.38	-1.86	4.50
360	78.53	-1.86	4.50
361	78.81	-1.87	4.50
362	79.04	-1.87	4.50
363	79.15	-1.89	4.50
364	79.24	-1.89	4.50
365	79.43	-1.89	4.50
366	79.7	-1.89	4.51
367	79.88	-1.90	4.51
368	80.18	-1.90	4.51
369	80.17	-1.92	4.51
370	80.39	-1.92	4.51
371	80.52	-1.92	4.51
372	80.83	-1.94	4.51
373	81	-1.94	4.51
374	81.26	-1.95	4.51
375	81.24	-1.95	4.51
376	81.44	-1.97	4.51
377	81.66	-1.95	4.51
378	81.9	-1.97	4.51
379	82.16	-1.97	4.51
380	82.3	-1.98	4.51
381	82.38	-1.98	4.51
382	82.53	-2.00	4.51
383	82.82	-2.00	4.51
384	82.98	-2.02	4.51
385	83.32	-2.02	4.51
386	83.33	-2.03	4.51
387	83.47	-2.03	4.51
388	83.57	-2.03	4.51
389	83.89	-2.05	4.51
390	84.06	-2.05	4.51
391	84.36	-2.06	4.51

356	77.45	-1.51	4.67
357	77.63	-1.52	4.67
358	77.92	-1.52	4.67
359	77.9	-1.52	4.67
360	78.1	-1.53	4.67
361	78.27	-1.53	4.67
362	78.53	-1.53	4.66
363	78.79	-1.54	4.66
364	78.96	-1.54	4.66
365	79.03	-1.54	4.66
366	79.15	-1.54	4.66
367	79.42	-1.54	4.66
368	79.57	-1.55	4.66
369	79.93	-1.55	4.66
370	79.99	-1.55	4.66
371	80.12	-1.56	4.66
372	80.19	-1.56	4.66
373	80.54	-1.56	4.66
374	80.67	-1.57	4.65
375	80.99	-1.57	4.65
376	81.05	-1.58	4.65
377	81.12	-1.58	4.65
378	81.33	-1.58	4.65
379	81.54	-1.59	4.65
380	81.8	-1.59	4.65
381	81.99	-1.60	4.65
382	82.15	-1.59	4.65
383	82.12	-1.60	4.65
384	82.47	-1.60	4.64
385	82.56	-1.60	4.64
386	82.93	-1.60	4.64
387	83.01	-1.61	4.64
388	83.22	-1.61	4.64
389	83.17	-1.61	4.64
390	83.49	-1.61	4.64
391	83.64	-1.62	4.64

356	68.21	-1.92	4.3475
357	68.34	-1.94	4.3475
358	68.37	-1.94	4.3475
359	68.55	-1.94	4.3475
360	68.83	-1.94	4.3475
361	68.98	-1.96	4.3475
362	69.33	-1.94	4.3475
363	69.35	-1.96	4.3475
364	69.46	-1.96	4.3475
365	69.58	-1.98	4.3475
366	69.89	-1.98	4.3475
367	70.03	-1.98	4.3475
368	70.35	-1.98	4.3475
369	70.42	-1.98	4.3475
370	70.47	-2.00	4.3475
371	70.68	-2.00	4.3475
372	70.91	-2.00	4.3475
373	71.18	-2.00	4.3475
374	71.38	-2.02	4.3475
375	71.54	-2.00	4.3475
376	71.52	-2.04	4.3475
377	71.81	-2.02	4.3475
378	71.93	-2.04	4.3475
379	72.27	-2.04	4.3475
380	72.39	-2.04	4.3475
381	72.62	-2.04	4.3475
382	72.54	-2.05	4.3475
383	72.92	-2.05	4.3475
384	72.98	-2.05	4.3475
385	73.32	-2.05	4.3475
386	73.47	-2.05	4.3475
387	73.63	-2.07	4.3475
388	73.64	-2.09	4.3475
389	73.9	-2.09	4.3475
390	74.12	-2.09	4.3475
391	74.33	-2.09	4.3475

356	76.66	-1.81	4.64
357	76.71	-1.81	4.64
358	76.85	-1.81	4.64
359	76.99	-1.81	4.64
360	77.32	-1.82	4.64
361	77.47	-1.82	4.64
362	77.78	-1.82	4.64
363	77.75	-1.82	4.64
364	77.95	-1.82	4.64
365	78.1	-1.83	4.64
366	78.36	-1.83	4.64
367	78.62	-1.83	4.64
368	78.81	-1.83	4.64
369	78.86	-1.83	4.64
370	78.95	-1.83	4.64
371	79.23	-1.84	4.64
372	79.38	-1.84	4.64
373	79.74	-1.84	4.64
374	79.79	-1.84	4.64
375	79.95	-1.84	4.64
376	79.99	-1.85	4.64
377	80.31	-1.85	4.64
378	80.43	-1.86	4.64
379	80.78	-1.86	4.64
380	80.83	-1.86	4.64
381	80.92	-1.86	4.64
382	81.07	-1.87	4.64
383	81.3	-1.87	4.64
384	81.52	-1.87	4.64
385	81.78	-1.87	4.64
386	81.92	-1.88	4.64
387	81.87	-1.88	4.64
388	82.17	-1.88	4.64
389	82.3	-1.88	4.64
390	82.62	-1.89	4.64
391	82.74	-1.89	4.64

392	84.43	-2.06	4.51
393	84.48	-2.08	4.51
394	84.73	-2.08	4.51
395	84.92	-2.10	4.51
396	85.22	-2.10	4.51
397	85.36	-2.11	4.51
398	85.54	-2.11	4.51
399	85.53	-2.13	4.51
400	85.85	-2.13	4.51
401	85.97	-2.14	4.51
402	86.36	-2.14	4.51
403	86.42	-2.16	4.51
404	86.59	-2.18	4.51
405	86.67	-2.18	4.51
406	86.94	-2.18	4.51
407	87.12	-2.18	4.51
408	87.45	-2.19	4.51
409	87.59	-2.19	4.53
410	87.61	-2.21	4.53
411	87.84	-2.21	4.53
412	88.04	-2.22	4.53
413	88.31	-2.22	4.53
414	88.48	-2.22	4.53
415	88.73	-2.24	4.53
416	88.64	-2.26	4.51
417	88.97	-2.26	4.51
418	89.07	-2.27	4.51
419	89.45	-2.27	4.51
420	89.55	-2.27	4.51
421	89.76	-2.29	4.51
422	89.76	-2.29	4.51
423	90.02	-2.30	4.51
424	90.21	-2.30	4.51
425	90.47	-2.30	4.51
426	90.71	-2.30	4.51
427	90.73	-2.32	4.51

392	83.96	-1.62	4.64
393	84.11	-1.63	4.64
394	84.2	-1.63	4.64
395	84.31	-1.63	4.63
396	84.52	-1.64	4.63
397	84.79	-1.64	4.63
398	84.98	-1.65	4.63
399	85.2	-1.65	4.63
400	85.16	-1.66	4.63
401	85.44	-1.66	4.63
402	85.54	-1.66	4.63
403	85.88	-1.66	4.63
404	86	-1.67	4.62
405	86.26	-1.67	4.62
406	86.19	-1.67	4.62
407	86.44	-1.67	4.62
408	86.62	-1.68	4.62
409	86.87	-1.67	4.62
410	87.12	-1.68	4.62
411	87.24	-1.68	4.62
412	87.29	-1.69	4.62
413	87.46	-1.69	4.62
414	87.73	-1.69	4.61
415	87.92	-1.70	4.61
416	88.23	-1.70	4.61
417	88.25	-1.71	4.61
418	88.37	-1.70	4.61
419	88.54	-1.71	4.61
420	88.84	-1.71	4.61
421	88.98	-1.71	4.61
422	89.27	-1.72	4.61
423	89.32	-1.72	4.61
424	89.43	-1.72	4.61
425	89.65	-1.73	4.61
426	89.86	-1.73	4.61
427	90.15	-1.73	4.60

392	74.57	-2.09	4.3475
393	74.6	-2.09	4.3475
394	74.78	-2.11	4.3475
395	74.92	-2.11	4.3475
396	75.2	-2.11	4.3475
397	75.39	-2.11	4.3475
398	75.66	-2.11	4.3475
399	75.6	-2.13	4.3475
400	75.82	-2.13	4.3475
401	75.99	-2.13	4.3475
402	76.25	-2.13	4.3475
403	76.45	-2.15	4.3475
404	76.68	-2.15	4.3475
405	76.7	-2.15	4.3475
406	76.85	-2.16	4.3475
407	77.07	-2.16	4.3475
408	77.27	-2.16	4.3475
409	77.57	-2.15	4.366
410	77.68	-2.16	4.366
411	77.79	-2.16	4.366
412	77.89	-2.18	4.366
413	78.19	-2.16	4.366
414	78.28	-2.18	4.366
415	78.68	-2.18	4.366
416	78.72	-2.18	4.366
417	78.85	-2.18	4.366
418	78.92	-2.20	4.366
419	79.23	-2.18	4.366
420	79.38	-2.20	4.366
421	79.67	-2.20	4.366
422	79.82	-2.20	4.366
423	79.85	-2.20	4.366
424	80.05	-2.20	4.366
425	80.24	-2.22	4.366
426	80.53	-2.20	4.366
427	80.7	-2.22	4.366

392	82.98	-1.89	4.64
393	82.86	-1.89	4.64
394	83.2	-1.90	4.64
395	83.29	-1.90	4.64
396	83.64	-1.90	4.64
397	83.78	-1.90	4.64
398	83.94	-1.90	4.64
399	83.97	-1.91	4.64
400	84.18	-1.90	4.64
401	84.41	-1.91	4.64
402	84.64	-1.91	4.64
403	84.89	-1.91	4.64
404	84.91	-1.91	4.64
405	85.03	-1.92	4.64
406	85.21	-1.91	4.64
407	85.48	-1.92	4.64
408	85.64	-1.93	4.64
409	85.95	-1.93	4.64
410	85.9	-1.93	4.64
411	86.11	-1.93	4.64
412	86.24	-1.93	4.64
413	86.54	-1.93	4.64
414	86.74	-1.94	4.64
415	86.93	-1.94	4.64
416	86.98	-1.94	4.64
417	87.1	-1.94	4.64
418	87.36	-1.94	4.64
419	87.5	-1.94	4.64
420	87.85	-1.95	4.64
421	87.92	-1.95	4.64
422	88.03	-1.95	4.64
423	88.12	-1.95	4.64
424	88.46	-1.95	4.64
425	88.56	-1.96	4.64
426	88.93	-1.97	4.64
427	88.97	-1.96	4.64

428	90.85	-2.34	4.51
429	91.03	-2.34	4.53
430	91.31	-2.34	4.53
431	91.49	-2.35	4.53
432	91.76	-2.35	4.53
433	91.71	-2.37	4.51
434	91.94	-2.37	4.51
435	92.05	-2.38	4.51
436	92.36	-2.38	4.51
437	92.53	-2.38	4.51
438	92.74	-2.40	4.51
439	92.75	-2.42	4.51
440	92.93	-2.42	4.51
441	93.13	-2.42	4.51
442	93.33	-2.43	4.51
443	93.61	-2.43	4.51
444	93.71	-2.45	4.51
445	93.8	-2.45	4.51
446	93.92	-2.46	4.51
447	94.23	-2.46	4.51
448	94.35	-2.46	4.51
449	94.68	-2.48	4.51
450	94.73	-2.48	4.51
451	94.84	-2.50	4.51
452	94.97	-2.50	4.51
453	95.24	-2.50	4.51
454	95.46	-2.50	4.51
455	95.68	-2.51	4.51
456	95.78	-2.53	4.51
457	95.84	-2.53	4.51
458	96.11	-2.53	4.51
459	96.27	-2.54	4.51
460	96.59	-2.53	4.51
461	96.73	-2.54	4.51
462	96.9	-2.56	4.51
463	96.89	-2.56	4.51

428	90.29	-1.74	4.60
429	90.43	-1.74	4.60
430	90.47	-1.74	4.60
431	90.78	-1.74	4.60
432	90.9	-1.74	4.60
433	91.28	-1.75	4.60
434	91.33	-1.75	4.60
435	91.48	-1.75	4.60
436	91.56	-1.76	4.60
437	91.86	-1.76	4.60
438	92	-1.76	4.60
439	92.3	-1.76	4.60
440	92.45	-1.77	4.60
441	92.47	-1.77	4.59
442	92.68	-1.77	4.59
443	92.89	-1.77	4.59
444	93.14	-1.77	4.59
445	93.33	-1.78	4.59
446	93.54	-1.78	4.59
447	93.5	-1.78	4.59
448	93.8	-1.79	4.59
449	93.9	-1.79	4.59
450	94.27	-1.79	4.58
451	94.37	-1.80	4.58
452	94.6	-1.80	4.58
453	94.53	-1.81	4.58
454	94.85	-1.81	4.58
455	95.01	-1.81	4.58
456	95.27	-1.81	4.58
457	95.48	-1.82	4.58
458	95.58	-1.82	4.58
459	95.66	-1.82	4.58
460	95.85	-1.82	4.58
461	96.13	-1.82	4.58
462	96.31	-1.82	4.58
463	96.59	-1.83	4.58

428	80.91	-2.20	4.366
429	80.87	-2.22	4.366
430	81.16	-2.22	4.366
431	81.27	-2.22	4.366
432	81.61	-2.22	4.366
433	81.76	-2.24	4.366
434	82	-2.24	4.366
435	81.92	-2.26	4.3845
436	82.24	-2.26	4.3845
437	82.38	-2.26	4.3845
438	82.63	-2.26	4.366
439	82.86	-2.26	4.366
440	83.02	-2.28	4.366
441	83.05	-2.28	4.366
442	83.24	-2.28	4.366
443	83.51	-2.28	4.366
444	83.67	-2.29	4.366
445	83.97	-2.28	4.366
446	84.02	-2.28	4.366
447	84.15	-2.29	4.366
448	84.28	-2.29	4.366
449	84.59	-2.29	4.366
450	84.74	-2.31	4.366
451	85.05	-2.29	4.366
452	85.05	-2.31	4.366
453	85.17	-2.31	4.3845
454	85.39	-2.31	4.3845
455	85.62	-2.33	4.3845
456	85.86	-2.31	4.3845
457	86.06	-2.33	4.3845
458	86.18	-2.33	4.3845
459	86.19	-2.35	4.3845
460	86.5	-2.35	4.366
461	86.64	-2.35	4.366
462	86.98	-2.35	4.366
463	87.1	-2.35	4.366

428	89.07	-1.97	4.64
429	89.21	-1.97	4.64
430	89.45	-1.97	4.64
431	89.67	-1.97	4.64
432	89.89	-1.97	4.64
433	90.03	-1.97	4.64
434	90.03	-1.97	4.64
435	90.29	-1.98	4.64
436	90.43	-1.97	4.64
437	90.75	-1.98	4.64
438	90.91	-1.98	4.64
439	91.14	-1.98	4.64
440	91.05	-1.98	4.64
441	91.4	-1.98	4.64
442	91.49	-1.98	4.64
443	91.83	-1.99	4.64
444	91.95	-1.99	4.64
445	92.12	-1.99	4.64
446	92.11	-1.99	4.64
447	92.37	-1.99	4.64
448	92.56	-1.99	4.64
449	92.82	-1.99	4.64
450	93.03	-2.00	4.64
451	93.09	-2.00	4.64
452	93.26	-2.01	4.64
453	93.4	-2.00	4.64
454	93.69	-2.01	4.64
455	93.86	-2.01	4.64
456	94.15	-2.01	4.64
457	94.11	-2.01	4.64
458	94.33	-2.02	4.64
459	94.46	-2.02	4.64
460	94.76	-2.02	4.64
461	94.95	-2.02	4.64
462	95.18	-2.02	4.64
463	95.21	-2.03	4.64

464	97.26	-2.56	4.51
465	97.34	-2.56	4.51
466	97.7	-2.58	4.51
467	97.78	-2.58	4.51
468	97.92	-2.59	4.51
469	98	-2.58	4.51
470	98.27	-2.59	4.51
471	98.49	-2.59	4.51
472	98.73	-2.59	4.51
473	98.88	-2.61	4.51
474	98.88	-2.61	4.50
475	99.15	-2.61	4.50
476	99.29	-2.62	4.50
477	99.59	-2.62	4.50
478	99.75	-2.64	4.50
479	99.97	-2.64	4.50
480	99.89	-2.66	4.50
481	100.21	-2.66	4.50
482	100.33	-2.66	4.50
483	100.63	-2.67	4.50
484	100.81	-2.67	4.50
485	100.97	-2.69	4.50
486	100.97	-2.69	4.50
487	101.21	-2.69	4.50
488	101.44	-2.69	4.50
489	101.68	-2.70	4.50
490	101.91	-2.70	4.50
491	101.95	-2.72	4.50
492	102.09	-2.72	4.50
493	102.25	-2.74	4.50
494	102.53	-2.74	4.50
495	102.7	-2.74	4.50
496	102.99	-2.75	4.50
497	102.94	-2.77	4.50
498	103.16	-2.77	4.50
499	103.3	-2.78	4.50

464	96.62	-1.83	4.58
465	96.78	-1.83	4.58
466	96.89	-1.83	4.58
467	97.22	-1.83	4.58
468	97.39	-1.84	4.58
469	97.64	-1.84	4.58
470	97.63	-1.85	4.57
471	97.82	-1.85	4.57
472	98.02	-1.85	4.57
473	98.23	-1.85	4.57
474	98.51	-1.86	4.57
475	98.65	-1.86	4.57
476	98.74	-1.87	4.57
477	98.83	-1.86	4.57
478	99.14	-1.87	4.57
479	99.26	-1.87	4.57
480	99.6	-1.87	4.57
481	99.65	-1.88	4.57
482	99.81	-1.88	4.56
483	99.87	-1.88	4.56
484	100.19	-1.88	4.56
485	100.35	-1.88	4.56
486	100.63	-1.88	4.56
487	100.75	-1.89	4.56
488	100.77	-1.89	4.56
489	101.01	-1.89	4.56
490	101.21	-1.89	4.56
491	101.47	-1.90	4.56
492	101.67	-1.90	4.56
493	101.84	-1.90	4.56
494	101.78	-1.91	4.56
495	102.1	-1.91	4.56
496	102.23	-1.91	4.56
497	102.58	-1.92	4.55
498	102.68	-1.92	4.55
499	102.91	-1.92	4.55

464	87.28	-2.35	4.366
465	87.27	-2.37	4.366
466	87.59	-2.37	4.366
467	87.69	-2.37	4.366
468	88.07	-2.37	4.366
469	88.18	-2.37	4.366
470	88.31	-2.37	4.366
471	88.37	-2.39	4.366
472	88.63	-2.39	4.366
473	88.84	-2.39	4.366
474	89.08	-2.39	4.366
475	89.31	-2.39	4.366
476	89.31	-2.41	4.366
477	89.48	-2.41	4.366
478	89.63	-2.42	4.366
479	89.93	-2.41	4.366
480	90.09	-2.42	4.366
481	90.37	-2.42	4.366
482	90.31	-2.42	4.366
483	90.55	-2.42	4.366
484	90.66	-2.44	4.366
485	90.97	-2.44	4.366
486	91.16	-2.42	4.366
487	91.37	-2.42	4.366
488	91.39	-2.44	4.366
489	91.58	-2.44	4.366
490	91.79	-2.44	4.366
491	91.98	-2.46	4.366
492	92.27	-2.44	4.366
493	92.38	-2.46	4.366
494	92.47	-2.46	4.366
495	92.58	-2.46	4.366
496	92.89	-2.46	4.366
497	93.01	-2.48	4.366
498	93.36	-2.46	4.366
499	93.42	-2.48	4.366

464	95.37	-2.03	4.64
465	95.6	-2.03	4.64
466	95.82	-2.03	4.64
467	96.12	-2.04	4.64
468	96.22	-2.04	4.64
469	96.34	-2.04	4.64
470	96.44	-2.04	4.64
471	96.73	-2.04	4.64
472	96.84	-2.04	4.64
473	97.2	-2.05	4.64
474	97.26	-2.05	4.64
475	97.37	-2.05	4.64
476	97.49	-2.05	4.64
477	97.79	-2.05	4.64
478	97.96	-2.05	4.64
479	98.26	-2.05	4.64
480	98.41	-2.05	4.64
481	98.4	-2.05	4.64
482	98.64	-2.05	4.64
483	98.82	-2.05	4.64
484	99.12	-2.06	4.64
485	99.27	-2.06	4.64
486	99.48	-2.06	4.64
487	99.43	-2.06	4.64
488	99.75	-2.06	4.64
489	99.83	-2.06	4.64
490	100.2	-2.07	4.64
491	100.31	-2.07	4.64
492	100.56	-2.07	4.64
493	100.51	-2.08	4.64
494	100.83	-2.07	4.64
495	100.98	-2.08	4.64
496	101.25	-2.08	4.64
497	101.46	-2.08	4.64
498	101.54	-2.08	4.64
499	101.65	-2.08	4.64

500	103.56	-2.78	4.50
501	103.76	-2.80	4.50
502	103.99	-2.80	4.50
503	104.01	-2.82	4.50
504	104.18	-2.83	4.50
505	104.42	-2.83	4.50
506	104.6	-2.83	4.50
507	104.9	-2.83	4.51
508	104.96	-2.85	4.50
509	105.11	-2.85	4.50
510	105.22	-2.86	4.50
511	105.52	-2.86	4.50
512	105.65	-2.86	4.50
513	105.99	-2.88	4.50
514	106.02	-2.88	4.50
515	106.13	-2.90	4.50
516	106.32	-2.90	4.50
517	106.55	-2.91	4.50
518	106.79	-2.91	4.50
519	107	-2.91	4.50
520	107.13	-2.93	4.50
521	107.13	-2.93	4.50
522	107.41	-2.94	4.50
523	107.59	-2.94	4.50
524	107.9	-2.94	4.50
525	108	-2.96	4.50
526	108.19	-2.96	4.50
527	108.19	-2.96	4.50
528	108.49	-2.98	4.50
529	108.61	-2.98	4.51
530	108.98	-2.98	4.51
531	109.06	-2.98	4.51
532	109.17	-2.99	4.51
533	109.28	-2.99	4.51
534	109.52	-3.01	4.51
535	109.72	-3.01	4.50

500	102.88	-1.93	4.55
501	103.19	-1.93	4.55
502	103.34	-1.93	4.55
503	103.65	-1.93	4.55
504	103.83	-1.93	4.55
505	103.89	-1.94	4.55
506	104.01	-1.95	4.55
507	104.21	-1.94	4.55
508	104.46	-1.95	4.55
509	104.66	-1.95	4.55
510	104.93	-1.95	4.55
511	104.88	-1.96	4.55
512	105.12	-1.97	4.55
513	105.25	-1.97	4.54
514	105.58	-1.97	4.54
515	105.72	-1.97	4.54
516	105.99	-1.98	4.54
517	105.96	-1.98	4.54
518	106.2	-1.98	4.54
519	106.37	-1.99	4.54
520	106.62	-1.99	4.54
521	106.85	-1.99	4.54
522	106.99	-1.99	4.54
523	107.06	-2.00	4.54
524	107.22	-2.00	4.54
525	107.5	-2.01	4.54
526	107.65	-2.01	4.54
527	107.98	-2.01	4.54
528	108.03	-2.01	4.54
529	108.14	-2.02	4.54
530	108.26	-2.02	4.54
531	108.57	-2.02	4.54
532	108.73	-2.02	4.54
533	109.01	-2.03	4.53
534	109.08	-2.03	4.53
535	109.15	-2.04	4.53

500	93.56	-2.48	4.366
501	93.63	-2.50	4.366
502	93.93	-2.50	4.366
503	94.1	-2.50	4.366
504	94.37	-2.50	4.366
505	94.5	-2.50	4.366
506	94.53	-2.52	4.366
507	94.75	-2.52	4.366
508	94.95	-2.52	4.366
509	95.22	-2.52	4.366
510	95.42	-2.52	4.366
511	95.61	-2.52	4.366
512	95.56	-2.53	4.366
513	95.89	-2.53	4.366
514	96.02	-2.53	4.366
515	96.34	-2.53	4.366
516	96.43	-2.55	4.366
517	96.7	-2.55	4.366
518	96.6	-2.57	4.366
519	96.92	-2.57	4.366
520	97.06	-2.57	4.366
521	97.36	-2.59	4.366
522	97.55	-2.59	4.366
523	97.67	-2.61	4.366
524	97.71	-2.61	4.366
525	97.95	-2.63	4.366
526	98.18	-2.63	4.366
527	98.37	-2.63	4.366
528	98.66	-2.63	4.366
529	98.69	-2.65	4.366
530	98.81	-2.65	4.366
531	98.96	-2.66	4.366
532	99.26	-2.66	4.366
533	99.4	-2.66	4.366
534	99.71	-2.66	4.366
535	99.74	-2.68	4.366

500	101.81	-2.08	4.64
501	102.09	-2.08	4.64
502	102.28	-2.09	4.64
503	102.55	-2.09	4.64
504	102.53	-2.09	4.64
505	102.76	-2.09	4.64
506	102.88	-2.09	4.64
507	103.18	-2.09	4.64
508	103.37	-2.09	4.64
509	103.61	-2.09	4.64
510	103.61	-2.10	4.64
511	103.78	-2.09	4.64
512	103.98	-2.10	4.64
513	104.19	-2.10	4.64
514	104.47	-2.10	4.64
515	104.61	-2.10	4.64
516	104.68	-2.10	4.64
517	104.79	-2.10	4.64
518	105.09	-2.10	4.64
519	105.24	-2.10	4.64
520	105.58	-2.11	4.64
521	105.62	-2.11	4.64
522	105.78	-2.11	4.64
523	105.85	-2.11	4.64
524	106.18	-2.12	4.64
525	106.31	-2.12	4.64
526	106.63	-2.12	4.64
527	106.73	-2.12	4.64
528	106.75	-2.12	4.64
529	106.99	-2.13	4.64
530	107.18	-2.13	4.64
531	107.46	-2.13	4.64
532	107.66	-2.13	4.64
533	107.86	-2.14	4.64
534	107.81	-2.14	4.64
535	108.13	-2.14	4.64

536	109.94	-3.02	4.50
537	110.16	-3.02	4.50
538	110.11	-3.04	4.50
539	110.36	-3.04	4.50
540	110.51	-3.06	4.50
541	110.82	-3.06	4.50
542	110.95	-3.07	4.50
543	111.18	-3.07	4.50
544	111.11	-3.09	4.50
545	111.42	-3.10	4.50
546	111.52	-3.10	4.50
547	111.85	-3.12	4.50
548	112.02	-3.12	4.50
549	112.18	-3.14	4.50
550	112.19	-3.14	4.50
551	112.45	-3.14	4.50
552	112.65	-3.14	4.50
553	112.86	-3.15	4.50
554	113.12	-3.15	4.51
555	113.18	-3.17	4.51
556	113.32	-3.17	4.51
557	113.46	-3.17	4.50
558	113.75	-3.17	4.50
559	113.92	-3.17	4.50
560	114.19	-3.18	4.50
561	114.17	-3.20	4.50
562	114.38	-3.20	4.51
563	114.53	-3.20	4.51
564	114.79	-3.20	4.51
565	115.01	-3.20	4.51
566	115.21	-3.22	4.51
567	115.26	-3.22	4.51
568	115.39	-3.23	4.51
569	115.67	-3.22	4.51
570	115.83	-3.23	4.53
571	116.13	-3.23	4.53

536	109.36	-2.04	4.53
537	109.55	-2.04	4.53
538	109.82	-2.05	4.53
539	109.99	-2.05	4.53
540	110.15	-2.05	4.53
541	110.15	-2.06	4.53
542	110.48	-2.06	4.53
543	110.6	-2.06	4.53
544	110.95	-2.07	4.53
545	111.05	-2.07	4.53
546	111.22	-2.07	4.53
547	111.22	-2.07	4.53
548	111.54	-2.08	4.53
549	111.68	-2.08	4.53
550	112	-2.08	4.53
551	112.14	-2.08	4.53
552	112.24	-2.09	4.53
553	112.38	-2.09	4.53
554	112.59	-2.09	4.53
555	112.85	-2.10	4.53
556	113.06	-2.10	4.53
557	113.29	-2.10	4.53
558	113.25	-2.10	4.53
559	113.52	-2.11	4.53
560	113.65	-2.11	4.53
561	113.96	-2.12	4.53
562	114.11	-2.11	4.53
563	114.38	-2.11	4.53
564	114.3	-2.12	4.53
565	114.58	-2.13	4.53
566	114.75	-2.13	4.53
567	115.03	-2.13	4.53
568	115.24	-2.13	4.53
569	115.39	-2.14	4.52
570	115.45	-2.14	4.52
571	115.64	-2.15	4.52

536	99.87	-2.68	4.366
537	100.05	-2.68	4.366
538	100.28	-2.70	4.366
539	100.55	-2.70	4.366
540	100.74	-2.70	4.366
541	100.84	-2.70	4.366
542	100.88	-2.72	4.366
543	101.18	-2.70	4.366
544	101.3	-2.72	4.366
545	101.64	-2.70	4.366
546	101.77	-2.72	4.366
547	101.94	-2.72	4.366
548	101.94	-2.72	4.366
549	102.31	-2.72	4.366
550	102.38	-2.74	4.366
551	102.73	-2.72	4.366
552	102.82	-2.72	4.366
553	102.97	-2.74	4.366
554	103.05	-2.74	4.366
555	103.3	-2.74	4.366
556	103.48	-2.74	4.366
557	103.73	-2.76	4.366
558	103.92	-2.76	4.366
559	103.94	-2.76	4.366
560	104.17	-2.76	4.366
561	104.31	-2.78	4.366
562	104.59	-2.78	4.3475
563	104.78	-2.78	4.3475
564	105.02	-2.78	4.3475
565	104.96	-2.78	4.3475
566	105.25	-2.78	4.3475
567	105.36	-2.79	4.3475
568	105.66	-2.79	4.3475
569	105.82	-2.79	4.3475
570	106.06	-2.79	4.3475
571	106.05	-2.79	4.3475

536	108.24	-2.14	4.64
537	108.59	-2.14	4.64
538	108.7	-2.15	4.64
539	108.94	-2.15	4.64
540	108.87	-2.15	4.64
541	109.18	-2.16	4.64
542	109.34	-2.16	4.64
543	109.64	-2.16	4.64
544	109.8	-2.16	4.64
545	109.92	-2.16	4.64
546	110.01	-2.16	4.64
547	110.2	-2.16	4.64
548	110.46	-2.17	4.64
549	110.64	-2.17	4.64
550	110.94	-2.17	4.64
551	110.9	-2.17	4.64
552	111.1	-2.17	4.64
553	111.24	-2.17	4.64
554	111.54	-2.18	4.64
555	111.69	-2.18	4.64
556	111.95	-2.19	4.64
557	111.96	-2.19	4.64
558	112.15	-2.19	4.64
559	112.35	-2.19	4.64
560	112.59	-2.19	4.64
561	112.85	-2.19	4.64
562	112.99	-2.19	4.64
563	113.05	-2.20	4.64
564	113.19	-2.20	4.64
565	113.44	-2.20	4.64
566	113.58	-2.20	4.64
567	113.92	-2.20	4.64
568	114	-2.20	4.64
569	114.11	-2.20	4.64
570	114.19	-2.20	4.64
571	114.52	-2.20	4.64

572	116.22	-3.23	4.53
573	116.34	-3.25	4.53
574	116.43	-3.25	4.53
575	116.74	-3.25	4.53
576	116.87	-3.25	4.53
577	117.2	-3.25	4.53
578	117.24	-3.26	4.53
579	117.36	-3.26	4.53
580	117.53	-3.26	4.53
581	117.77	-3.28	4.53
582	118	-3.28	4.53
583	118.24	-3.28	4.53
584	118.39	-3.30	4.53
585	118.37	-3.30	4.53
586	118.66	-3.30	4.53
587	118.8	-3.31	4.53
588	119.12	-3.31	4.53
589	119.23	-3.31	4.53
590	119.45	-3.33	4.53
591	119.42	-3.33	4.53
592	119.75	-3.33	4.53
593	119.85	-3.33	4.53
594	120.2	-3.34	4.53
595	120.29	-3.34	4.53
596	120.44	-3.36	4.53
597	120.54	-3.36	4.53
598	120.75	-3.38	4.53
599	120.96	-3.39	4.53
600	121.2	-3.39	4.53
601	121.41	-3.41	4.53
602	121.4	-3.42	4.53
603	121.64	-3.44	4.53
604	121.79	-3.44	4.53
605	122.08	-3.44	4.53
606	122.22	-3.46	4.53
607	122.49	-3.47	4.53

572	115.9	-2.15	4.52
573	116.09	-2.15	4.52
574	116.37	-2.16	4.52
575	116.43	-2.16	4.52
576	116.54	-2.16	4.52
577	116.7	-2.17	4.52
578	116.99	-2.17	4.52
579	117.15	-2.17	4.52
580	117.47	-2.18	4.52
581	117.47	-2.17	4.52
582	117.63	-2.18	4.52
583	117.82	-2.19	4.51
584	118.05	-2.19	4.51
585	118.31	-2.19	4.51
586	118.48	-2.19	4.51
587	118.62	-2.19	4.51
588	118.65	-2.20	4.51
589	118.95	-2.20	4.51
590	119.08	-2.20	4.51
591	119.43	-2.21	4.51
592	119.52	-2.21	4.51
593	119.68	-2.21	4.51
594	119.71	-2.21	4.51
595	120.06	-2.22	4.51
596	120.18	-2.22	4.51
597	120.5	-2.23	4.51
598	120.61	-2.23	4.51
599	120.67	-2.23	4.51
600	120.81	-2.23	4.51
601	121.03	-2.24	4.51
602	121.29	-2.24	4.51
603	121.47	-2.24	4.51
604	121.67	-2.24	4.51
605	121.64	-2.24	4.51
606	121.92	-2.25	4.51
607	122.04	-2.26	4.51

572	106.25	-2.81	4.3475
573	106.45	-2.81	4.3475
574	106.69	-2.81	4.3475
575	106.97	-2.81	4.3475
576	107.07	-2.81	4.3475
577	107.16	-2.81	4.366
578	107.28	-2.83	4.366
579	107.57	-2.83	4.366
580	107.72	-2.83	4.366
581	108.05	-2.83	4.366
582	108.09	-2.83	4.366
583	108.22	-2.85	4.366
584	108.33	-2.85	4.366
585	108.66	-2.85	4.366
586	108.78	-2.85	4.366
587	109.07	-2.85	4.366
588	109.2	-2.85	4.366
589	109.22	-2.87	4.366
590	109.44	-2.87	4.366
591	109.64	-2.87	4.366
592	109.91	-2.87	4.366
593	110.09	-2.87	4.366
594	110.26	-2.87	4.366
595	110.24	-2.89	4.366
596	110.54	-2.89	4.366
597	110.64	-2.89	4.366
598	110.98	-2.89	4.366
599	111.11	-2.89	4.366
600	111.32	-2.89	4.366
601	111.26	-2.90	4.366
602	111.61	-2.90	4.366
603	111.72	-2.90	4.366
604	112.03	-2.90	4.366
605	112.2	-2.92	4.366
606	112.35	-2.92	4.366
607	112.38	-2.92	4.366

572	114.64	-2.20	4.64
573	114.96	-2.21	4.64
574	115.06	-2.21	4.64
575	115.12	-2.21	4.64
576	115.3	-2.21	4.64
577	115.51	-2.21	4.64
578	115.78	-2.21	4.64
579	115.96	-2.21	4.64
580	116.13	-2.22	4.64
581	116.09	-2.22	4.64
582	116.38	-2.22	4.64
583	116.5	-2.22	4.64
584	116.81	-2.23	4.64
585	116.97	-2.23	4.64
586	117.19	-2.23	4.64
587	117.1	-2.23	4.64
588	117.43	-2.24	4.64
589	117.56	-2.24	4.64
590	117.86	-2.24	4.64
591	118.02	-2.24	4.64
592	118.17	-2.24	4.64
593	118.18	-2.25	4.64
594	118.42	-2.25	4.64
595	118.64	-2.25	4.64
596	118.87	-2.25	4.64
597	119.11	-2.26	4.64
598	119.13	-2.26	4.64
599	119.3	-2.26	4.64
600	119.45	-2.26	4.64
601	119.74	-2.27	4.64
602	119.9	-2.27	4.64
603	120.18	-2.27	4.64
604	120.16	-2.27	4.64
605	120.34	-2.27	4.64
606	120.51	-2.28	4.64
607	120.78	-2.28	4.64

608	122.41	-3.47	4.53
609	122.7	-3.49	4.53
610	122.85	-3.49	4.53
611	123.15	-3.50	4.53
612	123.33	-3.50	4.53
613	123.5	-3.50	4.53
614	123.52	-3.52	4.53
615	123.75	-3.52	4.53
616	123.97	-3.52	4.53
617	124.17	-3.54	4.53
618	124.47	-3.54	4.53
619	124.51	-3.55	4.53
620	124.64	-3.55	4.53
621	124.78	-3.57	4.53
622	125.08	-3.57	4.53
623	125.23	-3.58	4.53
624	125.55	-3.58	4.53
625	125.55	-3.60	4.53
626	125.72	-3.60	4.53
627	125.86	-3.60	4.53
628	126.11	-3.62	4.53
629	126.36	-3.60	4.53
630	126.54	-3.62	4.53
631	126.64	-3.62	4.53
632	126.74	-3.63	4.53
633	127	-3.63	4.53
634	127.14	-3.63	4.53
635	127.48	-3.63	4.53
636	127.56	-3.65	4.53
637	127.71	-3.66	4.53
638	127.8	-3.66	4.53
639	128.1	-3.66	4.53
640	128.21	-3.66	4.53
641	128.56	-3.68	4.53
642	128.62	-3.68	4.53
643	128.74	-3.70	4.53

608	122.37	-2.26	4.51
609	122.52	-2.26	4.51
610	122.74	-2.26	4.51
611	122.66	-2.26	4.51
612	122.97	-2.27	4.51
613	123.09	-2.27	4.51
614	123.39	-2.28	4.51
615	123.59	-2.28	4.51
616	123.73	-2.28	4.50
617	123.76	-2.28	4.50
618	123.96	-2.29	4.50
619	124.21	-2.29	4.50
620	124.42	-2.29	4.50
621	124.69	-2.29	4.50
622	124.71	-2.29	4.50
623	124.88	-2.29	4.50
624	125.02	-2.30	4.50
625	125.32	-2.30	4.50
626	125.48	-2.30	4.50
627	125.76	-2.31	4.50
628	125.72	-2.31	4.50
629	125.91	-2.31	4.50
630	126.08	-2.31	4.50
631	126.31	-2.32	4.50
632	126.56	-2.32	4.50
633	126.74	-2.32	4.50
634	126.82	-2.32	4.50
635	126.91	-2.33	4.50
636	127.2	-2.33	4.50
637	127.34	-2.33	4.50
638	127.68	-2.34	4.50
639	127.74	-2.34	4.50
640	127.89	-2.34	4.50
641	127.96	-2.35	4.50
642	128.27	-2.35	4.50
643	128.41	-2.36	4.50

608	112.61	-2.94	4.3845
609	112.85	-2.92	4.3845
610	113.05	-2.94	4.3845
611	113.31	-2.94	4.3845
612	113.33	-2.94	4.3845
613	113.48	-2.96	4.3845
614	113.63	-2.96	4.3845
615	113.93	-2.96	4.3845
616	114.11	-2.98	4.3845
617	114.39	-2.98	4.3845
618	114.38	-2.98	4.3845
619	114.56	-3.00	4.3845
620	114.73	-3.00	4.3845
621	114.97	-3.02	4.3845
622	115.19	-3.02	4.3845
623	115.42	-3.03	4.3845
624	115.46	-3.03	4.366
625	115.57	-3.05	4.366
626	115.82	-3.05	4.366
627	115.98	-3.07	4.366
628	116.31	-3.05	4.366
629	116.41	-3.07	4.366
630	116.57	-3.07	4.366
631	116.59	-3.09	4.366
632	116.92	-3.09	4.366
633	117.03	-3.09	4.366
634	117.41	-3.09	4.366
635	117.46	-3.09	4.366
636	117.59	-3.09	4.366
637	117.68	-3.09	4.366
638	117.93	-3.09	4.366
639	118.12	-3.11	4.366
640	118.38	-3.11	4.366
641	118.56	-3.11	4.366
642	118.61	-3.11	4.366
643	118.8	-3.11	4.366

608	120.98	-2.28	4.64
609	121.17	-2.28	4.64
610	121.21	-2.28	4.64
611	121.36	-2.28	4.64
612	121.61	-2.28	4.64
613	121.78	-2.28	4.64
614	122.08	-2.29	4.64
615	122.17	-2.29	4.64
616	122.31	-2.29	4.64
617	122.39	-2.29	4.64
618	122.71	-2.29	4.64
619	122.8	-2.30	4.64
620	123.15	-2.30	4.64
621	123.21	-2.30	4.64
622	123.33	-2.30	4.64
623	123.46	-2.31	4.64
624	123.72	-2.31	4.64
625	123.93	-2.31	4.64
626	124.18	-2.31	4.64
627	124.33	-2.31	4.64
628	124.36	-2.31	4.64
629	124.58	-2.32	4.64
630	124.72	-2.32	4.64
631	125.03	-2.32	4.64
632	125.18	-2.32	4.64
633	125.4	-2.33	4.64
634	125.32	-2.33	4.64
635	125.67	-2.33	4.64
636	125.74	-2.33	4.64
637	126.08	-2.34	4.64
638	126.22	-2.34	4.64
639	126.41	-2.34	4.64
640	126.42	-2.34	4.64
641	126.69	-2.34	4.64
642	126.89	-2.34	4.64
643	127.11	-2.34	4.64

644	128.89	-3.68	4.53
645	129.15	-3.68	4.53
646	129.36	-3.68	4.53
647	129.57	-3.70	4.53
648	129.74	-3.70	4.53
649	129.74	-3.70	4.53
650	130.02	-3.70	4.53
651	130.16	-3.71	4.53
652	130.5	-3.70	4.53
653	130.63	-3.71	4.53
654	130.83	-3.71	4.53
655	130.78	-3.73	4.53
656	131.11	-3.73	4.53
657	131.23	-3.73	4.53
658	131.55	-3.73	4.53
659	131.71	-3.73	4.51
660	131.83	-3.74	4.51
661	131.9	-3.74	4.51
662	132.14	-3.74	4.51
663	132.36	-3.74	4.51
664	132.6	-3.74	4.51
665	132.82	-3.74	4.51
666	132.85	-3.76	4.51
667	133.04	-3.76	4.51
668	133.17	-3.76	4.51
669	133.5	-3.76	4.51
670	133.66	-3.78	4.51
671	133.91	-3.78	4.51
672	133.86	-3.78	4.51
673	134.16	-3.78	4.51
674	134.27	-3.78	4.51
675	134.55	-3.79	4.51
676	134.77	-3.79	4.51
677	134.95	-3.79	4.51
678	134.98	-3.81	4.51
679	135.15	-3.81	4.51

644	128.73	-2.36	4.50
645	128.8	-2.36	4.50
646	128.88	-2.36	4.50
647	129.07	-2.36	4.50
648	129.28	-2.37	4.50
649	129.53	-2.37	4.50
650	129.76	-2.37	4.50
651	129.91	-2.37	4.50
652	129.87	-2.37	4.50
653	130.18	-2.37	4.50
654	130.3	-2.38	4.50
655	130.64	-2.38	4.50
656	130.73	-2.39	4.50
657	130.99	-2.39	4.50
658	130.89	-2.39	4.50
659	131.25	-2.39	4.50
660	131.35	-2.40	4.50
661	131.7	-2.40	4.50
662	131.86	-2.40	4.50
663	131.97	-2.40	4.50
664	132.03	-2.40	4.50
665	132.25	-2.41	4.50
666	132.48	-2.41	4.50
667	132.7	-2.41	4.50
668	132.96	-2.41	4.50
669	132.97	-2.42	4.50
670	133.13	-2.42	4.50
671	133.29	-2.42	4.50
672	133.57	-2.42	4.50
673	133.72	-2.43	4.50
674	134.01	-2.43	4.50
675	134	-2.43	4.50
676	134.21	-2.43	4.50
677	134.37	-2.44	4.50
678	134.67	-2.44	4.50
679	134.86	-2.45	4.50

644	118.98	-3.13	4.366
645	119.26	-3.13	4.366
646	119.42	-3.13	4.366
647	119.66	-3.11	4.366
648	119.63	-3.13	4.366
649	119.9	-3.13	4.366
650	120	-3.13	4.366
651	120.32	-3.15	4.366
652	120.46	-3.15	4.366
653	120.7	-3.15	4.366
654	120.66	-3.16	4.366
655	120.91	-3.16	4.366
656	121.09	-3.18	4.366
657	121.31	-3.18	4.366
658	121.57	-3.18	4.366
659	121.71	-3.20	4.366
660	121.76	-3.20	4.366
661	121.91	-3.22	4.366
662	122.19	-3.20	4.366
663	122.33	-3.22	4.366
664	122.64	-3.22	4.366
665	122.68	-3.22	4.366
666	122.8	-3.22	4.366
667	122.91	-3.24	4.366
668	123.23	-3.22	4.366
669	123.38	-3.24	4.366
670	123.69	-3.24	4.366
671	123.74	-3.24	4.366
672	123.84	-3.24	4.366
673	124.04	-3.26	4.366
674	124.26	-3.26	4.366
675	124.5	-3.26	4.366
676	124.7	-3.26	4.366
677	124.86	-3.26	4.366
678	124.84	-3.27	4.366
679	125.13	-3.27	4.366

644	127.33	-2.35	4.64
645	127.41	-2.35	4.64
646	127.51	-2.35	4.64
647	127.67	-2.35	4.64
648	127.95	-2.35	4.64
649	128.12	-2.35	4.64
650	128.42	-2.36	4.64
651	128.38	-2.36	4.64
652	128.61	-2.36	4.64
653	128.74	-2.36	4.64
654	129.01	-2.36	4.64
655	129.22	-2.36	4.64
656	129.45	-2.36	4.64
657	129.48	-2.36	4.64
658	129.6	-2.36	4.64
659	129.85	-2.36	4.64
660	130.03	-2.36	4.64
661	130.34	-2.37	4.64
662	130.44	-2.37	4.64
663	130.58	-2.38	4.64
664	130.65	-2.38	4.64
665	130.95	-2.38	4.64
666	131.08	-2.38	4.64
667	131.44	-2.38	4.64
668	131.5	-2.38	4.64
669	131.64	-2.39	4.64
670	131.74	-2.39	4.64
671	132.01	-2.39	4.64
672	132.17	-2.39	4.64
673	132.47	-2.39	4.64
674	132.59	-2.40	4.64
675	132.61	-2.40	4.64
676	132.85	-2.41	4.64
677	133.03	-2.41	4.64
678	133.3	-2.41	4.64
679	133.48	-2.41	4.64

680	135.44	-3.81	4.51
681	135.6	-3.82	4.51
682	135.89	-3.82	4.51
683	135.96	-3.84	4.51
684	136.08	-3.84	4.51
685	136.23	-3.86	4.53
686	136.52	-3.86	4.53
687	136.68	-3.87	4.53
688	136.98	-3.89	4.53
689	136.98	-3.89	4.53
690	137.14	-3.90	4.51
691	137.32	-3.90	4.51
692	137.57	-3.92	4.51
693	137.8	-3.92	4.51
694	138.01	-3.94	4.51
695	138.1	-3.94	4.51
696	138.18	-3.95	4.51
697	138.46	-3.95	4.51
698	138.6	-3.97	4.51
699	138.94	-3.97	4.51
700	139.02	-3.98	4.51
701	139.19	-4.00	4.51
702	139.26	-4.00	4.51
703	139.57	-4.00	4.51
704	139.7	-4.02	4.51
705	140.04	-4.02	4.51
706	140.13	-4.02	4.51
707	140.23	-4.03	4.51
708	140.38	-4.03	4.51
709	140.61	-4.05	4.51
710	140.85	-4.03	4.51
711	141.06	-4.05	4.51
712	141.25	-4.05	4.51
713	141.25	-4.06	4.51
714	141.52	-4.06	4.51
715	141.65	-4.06	4.51

680	135.04	-2.45	4.50
681	135.12	-2.45	4.51
682	135.26	-2.45	4.51
683	135.5	-2.45	4.51
684	135.66	-2.46	4.51
685	135.99	-2.46	4.51
686	136.07	-2.47	4.51
687	136.18	-2.46	4.51
688	136.31	-2.47	4.51
689	136.64	-2.47	4.51
690	136.74	-2.48	4.51
691	137.07	-2.48	4.50
692	137.14	-2.49	4.50
693	137.22	-2.49	4.50
694	137.4	-2.49	4.50
695	137.64	-2.50	4.50
696	137.87	-2.51	4.50
697	138.09	-2.51	4.50
698	138.24	-2.51	4.50
699	138.24	-2.51	4.50
700	138.53	-2.52	4.50
701	138.65	-2.52	4.50
702	139	-2.53	4.50
703	139.11	-2.53	4.50
704	139.3	-2.53	4.50
705	139.27	-2.54	4.50
706	139.62	-2.53	4.50
707	139.69	-2.54	4.50
708	140.04	-2.55	4.50
709	140.15	-2.55	4.50
710	140.31	-2.55	4.50
711	140.35	-2.56	4.49
712	140.61	-2.56	4.49
713	140.82	-2.57	4.49
714	141.07	-2.57	4.49
715	141.28	-2.58	4.49

680	125.27	-3.27	4.366
681	125.59	-3.27	4.366
682	125.72	-3.29	4.366
683	125.96	-3.29	4.3845
684	125.88	-3.29	4.3845
685	126.21	-3.29	4.3845
686	126.34	-3.29	4.3845
687	126.67	-3.31	4.3845
688	126.8	-3.31	4.3845
689	126.94	-3.31	4.3845
690	126.98	-3.33	4.3845
691	127.23	-3.33	4.3845
692	127.42	-3.33	4.3845
693	127.66	-3.35	4.3845
694	127.91	-3.33	4.3845
695	127.95	-3.35	4.3845
696	128.08	-3.35	4.3845
697	128.25	-3.37	4.3845
698	128.53	-3.37	4.3845
699	128.69	-3.37	4.3845
700	128.97	-3.37	4.3845
701	128.97	-3.39	4.3845
702	129.14	-3.39	4.3845
703	129.27	-3.40	4.3845
704	129.55	-3.40	4.3845
705	129.73	-3.40	4.3845
706	129.96	-3.40	4.3845
707	130.02	-3.40	4.3845
708	130.16	-3.42	4.3845
709	130.38	-3.42	4.3845
710	130.55	-3.42	4.3845
711	130.86	-3.42	4.3845
712	130.98	-3.42	4.3845
713	131.11	-3.42	4.3845
714	131.17	-3.44	4.3845
715	131.47	-3.44	4.3845

680	133.72	-2.41	4.64
681	133.66	-2.42	4.64
682	133.97	-2.42	4.64
683	134.08	-2.42	4.64
684	134.41	-2.42	4.64
685	134.54	-2.42	4.64
686	134.75	-2.43	4.64
687	134.72	-2.43	4.64
688	135	-2.43	4.64
689	135.16	-2.43	4.64
690	135.44	-2.43	4.64
691	135.65	-2.44	4.64
692	135.76	-2.44	4.64
693	135.82	-2.44	4.64
694	136.01	-2.44	4.64
695	136.28	-2.45	4.64
696	136.44	-2.45	4.64
697	136.74	-2.45	4.64
698	136.75	-2.45	4.64
699	136.91	-2.46	4.64
700	137.02	-2.46	4.64
701	137.34	-2.46	4.64
702	137.49	-2.46	4.64
703	137.75	-2.46	4.64
704	137.78	-2.47	4.64
705	137.93	-2.47	4.64
706	138.14	-2.47	4.64
707	138.35	-2.47	4.64
708	138.63	-2.48	4.64
709	138.75	-2.48	4.64
710	138.85	-2.49	4.64
711	138.92	-2.49	4.64
712	139.22	-2.49	4.64
713	139.32	-2.49	4.64
714	139.65	-2.50	4.64
715	139.75	-2.50	4.64

716	141.98	-4.06	4.51
717	142.11	-4.08	4.51
718	142.34	-4.08	4.51
719	142.29	-4.10	4.51
720	142.61	-4.11	4.51
721	142.72	-4.11	4.51
722	143.07	-4.11	4.51
723	143.23	-4.11	4.51
724	143.35	-4.13	4.51
725	143.42	-4.13	4.51
726	143.65	-4.14	4.53
727	143.86	-4.14	4.53
728	144.1	-4.14	4.53
729	144.34	-4.16	4.53
730	144.36	-4.16	4.53
731	144.54	-4.18	4.51
732	144.68	-4.18	4.51
733	144.98	-4.18	4.51
734	145.13	-4.19	4.51
735	145.4	-4.19	4.51
736	145.37	-4.21	4.51
737	145.6	-4.22	4.51
738	145.75	-4.22	4.51
739	146.02	-4.24	4.51
740	146.22	-4.24	4.51
741	146.39	-4.26	4.51
742	146.42	-4.26	4.51
743	146.61	-4.27	4.51
744	146.86	-4.27	4.51
745	147.02	-4.29	4.51
746	147.32	-4.29	4.51
747	147.39	-4.30	4.51
748	147.49	-4.30	4.53
749	147.62	-4.32	4.53
750	147.94	-4.32	4.53
751	148.05	-4.32	4.53

716	141.3	-2.58	4.49
717	141.49	-2.58	4.49
718	141.64	-2.58	4.49
719	141.93	-2.59	4.49
720	142.12	-2.60	4.49
721	142.36	-2.59	4.49
722	142.32	-2.60	4.49
723	142.57	-2.60	4.49
724	142.72	-2.60	4.49
725	142.99	-2.60	4.49
726	143.18	-2.61	4.49
727	143.41	-2.61	4.49
728	143.41	-2.62	4.49
729	143.61	-2.62	4.49
730	143.84	-2.62	4.49
731	144.04	-2.62	4.49
732	144.3	-2.63	4.49
733	144.4	-2.93	4.49
734	144.52	-2.94	4.49
735	144.64	-2.94	4.49
736	144.93	-2.94	4.49
737	145.04	-2.95	4.49
738	145.37	-2.95	4.49
739	145.39	-2.95	4.49
740	145.52	-2.95	4.49
741	145.68	-2.96	4.49
742	145.94	-2.96	4.49
743	146.13	-2.96	4.49
744	146.36	-2.96	4.49
745	146.47	-2.97	4.49
746	146.49	-2.97	4.49
747	146.76	-2.97	4.49
748	146.9	-2.97	4.49
749	147.21	-2.98	4.49
750	147.34	-2.98	4.49
751	147.51	-2.99	4.49

716	131.57	-3.44	4.3845
717	131.92	-3.44	4.3845
718	132	-3.46	4.3845
719	132.16	-3.46	4.3845
720	132.2	-3.46	4.3845
721	132.5	-3.46	4.3845
722	132.68	-3.48	4.3845
723	132.96	-3.48	4.3845
724	133.09	-3.48	4.366
725	133.16	-3.48	4.366
726	133.35	-3.48	4.366
727	133.54	-3.50	4.366
728	133.79	-3.50	4.366
729	134	-3.50	4.366
730	134.21	-3.50	4.366
731	134.16	-3.50	4.366
732	134.44	-3.52	4.366
733	134.58	-3.52	4.366
734	134.87	-3.52	4.366
735	135.01	-3.53	4.366
736	135.28	-3.53	4.366
737	135.23	-3.55	4.366
738	135.49	-3.55	4.366
739	135.66	-3.57	4.366
740	135.91	-3.57	4.366
741	136.13	-3.59	4.366
742	136.28	-3.59	4.366
743	136.34	-3.61	4.366
744	136.5	-3.63	4.366
745	136.74	-3.63	4.366
746	136.91	-3.64	4.366
747	137.21	-3.64	4.366
748	137.28	-3.64	4.366
749	137.4	-3.66	4.366
750	137.53	-3.66	4.366
751	137.81	-3.66	4.366

716	139.88	-2.50	4.64
717	139.9	-2.50	4.64
718	140.24	-2.50	4.64
719	140.36	-2.51	4.64
720	140.68	-2.51	4.64
721	140.79	-2.51	4.64
722	140.86	-2.51	4.64
723	141.02	-2.52	4.64
724	141.22	-2.52	4.64
725	141.46	-2.52	4.64
726	141.66	-2.53	4.64
727	141.85	-2.53	4.64
728	141.81	-2.53	4.64
729	142.11	-2.53	4.64
730	142.2	-2.53	4.64
731	142.52	-2.54	4.64
732	142.65	-2.54	4.64
733	142.91	-2.54	4.64
734	142.84	-2.55	4.64
735	143.13	-2.55	4.64
736	143.28	-2.56	4.64
737	143.53	-2.56	4.64
738	143.71	-2.57	4.64
739	143.88	-2.57	4.64
740	143.9	-2.57	4.64
741	144.12	-2.57	4.64
742	144.34	-2.58	4.64
743	144.56	-2.58	4.64
744	144.82	-2.58	4.64
745	144.86	-2.58	4.64
746	145	-2.59	4.64
747	145.16	-2.59	4.64
748	145.45	-2.60	4.64
749	145.59	-2.60	4.64
750	145.91	-2.60	4.64
751	145.9	-2.60	4.64

752	148.37	-4.34	4.53
753	148.4	-4.35	4.53
754	148.51	-4.37	4.53
755	148.7	-4.37	4.53
756	148.91	-4.38	4.53
757	149.2	-4.38	4.51
758	149.38	-4.38	4.51
759	149.5	-4.40	4.51
760	149.55	-4.42	4.51
761	149.81	-4.42	4.51
762	149.94	-4.43	4.51
763	150.3	-4.43	4.51
764	150.4	-4.45	4.51
765	150.56	-4.46	4.51
766	150.58	-4.46	4.51
767	150.9	-4.46	4.51
768	151.01	-4.46	4.51
769	151.34	-4.48	4.51
770	151.45	-4.48	4.51
771	151.56	-4.50	4.51
772	151.69	-4.50	4.51
773	151.9	-4.51	4.51
774	152.14	-4.50	4.51
775	152.34	-4.51	4.51
776	152.54	-4.53	4.51
777	152.52	-4.54	4.51
778	152.79	-4.54	4.51
779	152.91	-4.56	4.51
780	153.2	-4.56	4.51
781	153.35	-4.58	4.51
782	153.56	-4.58	4.51
783	153.49	-4.59	4.51
784	153.81	-4.61	4.51
785	153.95	-4.61	4.51
786	154.23	-4.62	4.51
787	154.39	-4.62	4.51

752	147.49	-2.99	4.49
753	147.79	-2.99	4.49
754	147.9	-2.99	4.49
755	148.26	-3.29	4.49
756	148.35	-3.00	4.49
757	148.53	-3.00	4.49
758	148.54	-3.01	4.49
759	148.82	-3.00	4.49
760	148.97	-3.01	4.49
761	149.24	-3.01	4.49
762	149.42	-3.02	4.49
763	149.46	-3.02	4.49
764	149.63	-3.02	4.49
765	149.77	-3.02	4.49
766	150.07	-3.03	4.49
767	150.24	-3.03	4.49
768	150.5	-3.44	4.49
769	150.46	-3.44	4.49
770	150.72	-3.44	4.49
771	150.82	-3.44	4.49
772	151.11	-3.44	4.49
773	151.27	-3.43	4.49
774	151.5	-3.42	4.49
775	151.5	-3.41	4.49
776	151.7	-3.40	4.49
777	151.93	-3.39	4.49
778	152.14	-3.38	4.49
779	152.38	-3.36	4.49
780	152.49	-3.35	4.49
781	152.58	-3.34	4.49
782	152.74	-3.33	4.49
783	153.01	-3.32	4.49
784	153.18	-3.31	4.49
785	153.47	-3.30	4.49
786	153.52	-3.30	4.49
787	153.66	-3.30	4.49

752	137.94	-3.68	4.366
753	138.27	-3.68	4.366
754	138.32	-3.68	4.366
755	138.42	-3.70	4.366
756	138.58	-3.70	4.366
757	138.83	-3.70	4.366
758	139.03	-3.72	4.3845
759	139.23	-3.72	4.366
760	139.38	-3.72	4.366
761	139.39	-3.74	4.366
762	139.66	-3.74	4.366
763	139.8	-3.76	4.366
764	140.1	-3.74	4.366
765	140.24	-3.76	4.366
766	140.44	-3.76	4.366
767	140.38	-3.77	4.366
768	140.72	-3.77	4.366
769	140.8	-3.77	4.366
770	141.13	-3.77	4.366
771	141.28	-3.77	4.366
772	141.47	-3.77	4.366
773	141.44	-3.79	4.366
774	141.72	-3.79	4.366
775	141.91	-3.79	4.366
776	142.17	-3.81	4.366
777	142.38	-3.79	4.366
778	142.46	-3.81	4.366
779	142.61	-3.81	4.366
780	142.76	-3.81	4.366
781	143.04	-3.81	4.366
782	143.21	-3.83	4.366
783	143.5	-3.83	4.366
784	143.49	-3.83	4.366
785	143.67	-3.85	4.366
786	143.8	-3.85	4.366
787	144.1	-3.85	4.366

752	146.05	-2.60	4.64
753	146.23	-2.61	4.64
754	146.46	-2.61	4.64
755	146.69	-2.61	4.64
756	146.89	-2.62	4.64
757	147	-2.62	4.64
758	147.06	-2.62	4.64
759	147.34	-2.62	4.64
760	147.48	-2.63	4.64
761	147.83	-2.64	4.64
762	147.92	-2.64	4.64
763	148.08	-2.64	4.64
764	148.1	-2.65	4.64
765	148.43	-2.65	4.64
766	148.53	-2.66	4.64
767	148.88	-2.67	4.64
768	148.96	-2.68	4.64
769	149.08	-2.68	4.64
770	149.18	-2.69	4.64
771	149.43	-2.70	4.64
772	149.66	-2.71	4.64
773	149.88	-2.72	4.64
774	150.08	-2.73	4.64
775	150.1	-2.73	4.64
776	150.31	-2.75	4.64
777	150.45	-2.76	4.64
778	150.75	-2.78	4.64
779	150.91	-2.79	4.64
780	151.14	-2.81	4.64
781	151.08	-2.83	4.64
782	151.38	-2.85	4.64
783	151.5	-2.87	4.64
784	151.81	-2.91	4.64
785	151.97	-2.94	4.64
786	152.17	-2.98	4.64
787	152.16	-3.03	4.64

788	154.53	-4.64	4.53
789	154.58	-4.66	4.53
790	154.8	-4.66	4.53
791	155.04	-4.67	4.53
792	155.24	-4.67	4.51
793	155.49	-4.69	4.51
794	155.5	-4.70	4.51
795	155.67	-4.72	4.51
796	155.81	-4.74	4.51
797	156.1	-4.75	4.51
798	156.27	-4.77	4.51
799	156.55	-4.78	4.51
800	156.51	-4.80	4.51
801	156.73	-4.82	4.51
802	156.93	-4.83	4.53
803	157.15	-4.85	4.53
804	157.38	-4.88	4.53
805	157.54	-4.90	4.53
806	157.61	-4.93	4.53
807	157.76	-4.96	4.53
808	158	-4.99	4.53
809	158.16	-5.04	4.53
810	158.45	-5.07	4.53
811	158.51	-5.14	4.53
812	158.61	-5.20	4.53
813	158.73	-5.28	4.53
814	159	-5.38	4.53
815	159.11	-5.49	4.53
816	159.4	-5.63	4.53
817	159.41	-5.79	4.53
818	159.43	-5.98	4.53
819	159.57	-6.22	4.53
820	159.75	-6.51	4.53
821	159.9	-6.83	4.53
822	160	-7.25	4.53
823	160.08	-7.71	4.53

788	153.77	-3.30	4.49
789	154.06	-3.31	4.49
790	154.23	-3.32	4.49
791	154.51	-3.33	4.49
792	154.6	-3.34	4.49
793	154.65	-3.35	4.49
794	154.91	-3.37	4.49
795	155.1	-3.38	4.49
796	155.39	-3.40	4.49
797	155.55	-3.42	4.49
798	155.72	-3.43	4.49
799	155.7	-3.44	4.49
800	156.02	-3.45	4.49
801	156.12	-3.46	4.49
802	156.48	-3.47	4.49
803	156.57	-3.47	4.49
804	156.73	-3.46	4.49
805	156.77	-3.45	4.49
806	157.06	-3.44	4.49
807	157.21	-3.43	4.49
808	157.51	-3.42	4.49
809	157.67	-3.40	4.49
810	157.71	-3.38	4.49
811	157.88	-3.36	4.49
812	158.08	-3.35	4.49
813	158.32	-3.33	4.49
814	158.49	-3.32	4.49
815	158.75	-3.29	4.49
816	158.69	-3.28	4.49
817	158.95	-3.26	4.49
818	159.06	-3.24	4.49
819	159.37	-3.23	4.49
820	159.51	-3.22	4.49
821	159.74	-3.20	4.49
822	159.74	-3.19	4.49
823	159.96	-3.18	4.49

788	144.27	-3.87	4.366
789	144.56	-3.87	4.366
790	144.6	-3.87	4.366
791	144.72	-3.89	4.3845
792	144.95	-3.89	4.3845
793	145.15	-3.90	4.3845
794	145.42	-3.90	4.3845
795	145.57	-3.92	4.3845
796	145.69	-3.92	4.3845
797	145.76	-3.94	4.3845
798	146.05	-3.96	4.3845
799	146.16	-3.98	4.3845
800	146.51	-3.98	4.3845
801	146.59	-4.00	4.3845
802	146.74	-4.01	4.3845
803	146.8	-4.03	4.3845
804	147.1	-4.05	4.3845
805	147.23	-4.07	4.3845
806	147.54	-4.07	4.403
807	147.67	-4.09	4.403
808	147.74	-4.11	4.403
809	147.87	-4.13	4.403
810	148.1	-4.14	4.403
811	148.31	-4.14	4.403
812	148.52	-4.16	4.403
813	148.74	-4.16	4.403
814	148.73	-4.20	4.4215
815	148.96	-4.20	4.4215
816	149.07	-4.22	4.4215
817	149.39	-4.24	4.4215
818	149.53	-4.26	4.4215
819	149.78	-4.26	4.4215
820	149.72	-4.27	4.4215
821	149.99	-4.29	4.4215
822	150.11	-4.31	4.4215
823	150.38	-4.33	4.4215

788	152.41	-3.08	4.64
789	152.64	-3.15	4.64
790	152.82	-3.22	4.64
791	153.08	-3.32	4.64
792	153.16	-3.42	4.64
793	153.26	-3.55	4.64
794	153.4	-3.70	4.64
795	153.69	-3.88	4.64
796	153.83	-4.09	4.64
797	154.14	-4.33	4.64
798	154.13	-4.60	4.64
799	154.29	-4.91	4.64
800	154.45	-5.27	4.64
801	154.71	-5.65	4.64
802	154.91	-6.08	4.64
803	155.14	-6.53	4.64
804	155.21	-7.01	4.64
805	155.28	-7.52	4.64
806	155.53	-8.05	4.64
807	155.7	-8.60	4.64
808	156	-9.17	4.64
809	156.11	-9.75	4.64
810	156.3	-10.35	4.64
811	156.31	-10.95	4.64
812	156.62	-11.57	4.64
813	156.7	-12.19	4.64
814	157.07	-12.83	4.64
815	157.15	-13.46	4.64
816	157.31	-14.11	4.64
817	157.37	-14.77	4.64
818	157.63	-15.42	4.64
819	157.8	-16.08	4.64
820	158.04	-16.74	4.64
821	158.22	-17.41	4.64
822	158.26	-18.08	4.64
823	158.44	-18.75	4.64

824	159.97	-8.26	4.53
825	160.17	-8.88	4.53
826	160.15	-9.60	4.53
827	160.39	-10.40	4.53
828	160.37	-11.28	4.53
829	160.43	-12.26	4.53
830	160.32	-13.31	4.54
831	160.54	-14.42	4.54
832	160.55	-15.60	4.54
833	160.77	-16.82	4.54
834	160.83	-18.06	4.54
835	160.88	-19.33	4.54
836	160.83	-18.06	4.54
837	160.98	-20.56	4.54
838	161.17	-21.74	4.54
839	161.44	-22.85	4.54
840	161.68	-23.86	4.54
841	161.96	-24.74	4.54
842	162.03	-25.47	4.54
843	162.37	-26.03	4.54
844	162.59	-26.40	4.54
845	162.99	-26.58	4.54
846	163.23	-26.58	4.54
847	162.99	-26.58	4.54
848	163.54	-26.40	4.54
849	163.56	-26.08	4.54
850	163.92	-25.63	4.54
851	164.11	-25.09	4.54
852	164.43	-24.48	4.54
853	164.7	-23.82	4.54
854	164.9	-23.15	4.54
855	164.98	-22.46	4.54
856	165.23	-21.79	4.54
857	165.51	-21.12	4.54
858	165.74	-20.46	4.54
859	165.51	-21.12	4.54

824	160.14	-3.17	4.49
825	160.37	-3.16	4.49
826	160.61	-3.15	4.49
827	160.71	-3.15	4.49
828	160.77	-3.15	4.49
829	160.94	-3.14	4.49
830	161.2	-3.14	4.49
831	161.37	-3.15	4.49
832	161.65	-3.15	4.49
833	161.69	-3.16	4.49
834	161.82	-3.17	4.49
835	161.95	-3.18	4.49
836	162.25	-3.20	4.49
837	162.4	-3.22	4.49
838	162.66	-3.24	4.49
839	162.71	-3.26	4.49
840	162.82	-3.28	4.49
841	163.02	-3.31	4.49
842	163.21	-3.33	4.49
843	163.49	-3.36	4.49
844	163.61	-3.39	4.49
845	163.74	-3.41	4.49
846	163.78	-3.43	4.49
847	164.08	-3.45	4.49
848	164.21	-3.47	4.49
849	164.55	-3.48	4.49
850	164.64	-3.49	4.49
851	164.78	-3.48	4.49
852	164.82	-3.47	4.49
853	165.12	-3.46	4.49
854	165.26	-3.44	4.49
855	165.56	-3.41	4.49
856	165.67	-3.38	4.49
857	165.76	-3.35	4.49
858	165.92	-3.31	4.49
859	166.11	-3.27	4.49

824	150.58	-4.33	4.4215
825	150.77	-4.35	4.4215
826	150.77	-4.37	4.4215
827	150.96	-4.40	4.4215
828	151.19	-4.40	4.4215
829	151.37	-4.44	4.4215
830	151.64	-4.44	4.4215
831	151.74	-4.46	4.4215
832	151.84	-4.48	4.44
833	151.96	-4.51	4.44
834	152.25	-4.53	4.44
835	152.37	-4.57	4.44
836	152.68	-4.59	4.44
837	152.72	-4.63	4.44
838	152.84	-4.66	4.44
839	152.98	-4.70	4.44
840	153.23	-4.72	4.44
841	153.4	-4.75	4.44
842	153.66	-4.79	4.44
843	153.79	-4.85	4.44
844	153.82	-4.90	4.44
845	154.06	-4.94	4.44
846	154.23	-5.00	4.44
847	154.5	-5.05	4.44
848	154.66	-5.12	4.44
849	154.85	-5.18	4.44
850	154.8	-5.27	4.44
851	155.11	-5.35	4.44
852	155.21	-5.44	4.44
853	155.54	-5.53	4.44
854	155.64	-5.62	4.44
855	155.86	-5.75	4.44
856	155.81	-5.88	4.44
857	156.11	-6.03	4.44
858	156.25	-6.18	4.44
859	156.51	-6.35	4.44

824	158.6	-19.43	4.64
825	158.86	-20.11	4.64
826	159.06	-20.79	4.64
827	159.37	-21.48	4.64
828	159.51	-22.16	4.64
829	159.74	-22.85	4.64
830	159.74	-23.54	4.64
831	159.96	-24.24	4.64
832	160.14	-24.93	4.64
833	160.37	-25.62	4.64
834	160.61	-26.32	4.64
835	160.71	-27.00	4.64
836	160.77	-27.69	4.64
837	160.94	-28.37	4.64
838	161.2	-29.05	4.64
839	161.37	-29.72	4.64
840	161.65	-30.37	4.64
841	161.69	-31.00	4.64
842	161.82	-31.64	4.64
843	161.95	-32.24	4.64
844	162.25	-32.83	4.64
845	162.4	-33.38	4.64
846	162.66	-33.91	4.64
847	162.71	-34.42	4.64
848	162.82	-34.90	4.64
849	163.02	-35.34	4.64
850	163.21	-35.75	4.64
851	163.49	-36.13	4.64
852	163.61	-36.47	4.64
853	163.74	-36.79	4.64
854	163.78	-37.06	4.64
855	164.08	-37.31	4.64
856	164.21	-37.52	4.64
857	164.55	-37.69	4.64
858	164.64	-37.82	4.64
859	164.78	-37.91	4.64

860	166.02	-19.82	4.54
861	166.08	-19.22	4.54
862	166.26	-18.62	4.54
863	166.42	-18.06	4.54
864	166.73	-17.54	4.54
865	166.93	-17.02	4.54
866	167.22	-16.53	4.54
867	167.25	-16.06	4.54
868	167.44	-15.63	4.54
869	167.63	-15.22	4.54
870	167.88	-14.82	4.54
871	168.13	-14.43	4.54
872	168.32	-14.08	4.54
873	168.41	-13.74	4.54
874	168.53	-13.42	4.54
875	168.79	-13.10	4.53
876	168.95	-12.82	4.53
877	169.26	-12.53	4.53
878	169.35	-12.27	4.53
879	169.49	-12.03	4.53
880	169.58	-11.79	4.53
881	169.87	-11.57	4.53
882	170.02	-11.34	4.53
883	170.32	-11.15	4.53
884	170.41	-10.96	4.53
885	170.49	-10.77	4.53
886	170.69	-10.59	4.53
887	170.9	-10.43	4.53
888	171.14	-10.26	4.53
889	171.33	-10.11	4.53
890	171.5	-9.95	4.53
891	171.49	-9.81	4.54
892	171.77	-9.66	4.54
893	171.9	-9.54	4.54
894	172.23	-9.41	4.54
895	172.35	-9.28	4.54

860	166.36	-3.23	4.49
861	166.56	-3.19	4.49
862	166.76	-3.15	4.49
863	166.72	-3.11	4.49
864	167.01	-3.07	4.49
865	167.11	-3.04	4.49
866	167.43	-3.00	4.49
867	167.54	-2.97	4.49
868	167.79	-2.94	4.49
869	167.71	-2.91	4.49
870	167.98	-2.88	4.49
871	168.14	-2.85	4.49
872	168.4	-2.82	4.49
873	168.6	-2.80	4.49
874	168.73	-2.73	4.49
875	168.79	-2.74	4.49
876	168.97	-2.74	4.49
877	169.21	-2.74	4.49
878	169.4	-2.74	4.49
879	169.66	-2.75	4.49
880	169.66	-2.75	4.49
881	169.83	-2.76	4.49
882	169.94	-2.76	4.49
883	170.23	-2.76	4.49
884	170.37	-2.76	4.49
885	170.64	-2.77	4.49
886	170.65	-2.77	4.49
887	170.79	-2.78	4.49
888	170.99	-2.78	4.49
889	171.2	-2.79	4.49
890	171.44	-2.79	4.49
891	171.57	-2.80	4.49
892	171.68	-2.80	4.49
893	171.75	-2.80	4.49
894	172.02	-2.81	4.49
895	172.15	-2.81	4.49

860	156.7	-6.53	4.44
861	156.78	-6.73	4.44
862	156.84	-6.97	4.44
863	157.05	-7.22	4.44
864	157.25	-7.47	4.44
865	157.44	-7.79	4.44
866	157.68	-8.10	4.44
867	157.66	-8.45	4.4215
868	157.8	-8.86	4.4215
869	157.9	-9.29	4.4215
870	158.18	-9.77	4.4215
871	158.32	-10.29	4.4215
872	158.56	-10.84	4.44
873	158.55	-11.47	4.44
874	158.67	-12.15	4.44
875	158.79	-12.88	4.44
876	158.97	-13.65	4.44
877	159.17	-14.47	4.44
878	159.29	-15.34	4.44
879	159.32	-16.21	4.44
880	159.34	-17.11	4.44
881	159.55	-17.98	4.44
882	159.6	-18.83	4.44
883	159.85	-19.59	4.44
884	159.9	-20.29	4.44
885	159.96	-20.89	4.44
886	159.91	-21.37	4.44
887	160.16	-21.68	4.44
888	160.2	-21.89	4.4215
889	160.47	-21.92	4.4215
890	160.49	-21.83	4.4215
891	160.6	-21.63	4.4215
892	160.68	-21.31	4.4215
893	160.91	-20.92	4.4215
894	161.13	-20.44	4.4215
895	161.42	-19.94	4.4215

860	164.82	-37.95	4.64
861	165.12	-37.96	4.64
862	165.26	-37.93	4.64
863	165.56	-37.84	4.64
864	165.67	-37.71	4.64
865	165.76	-37.54	4.64
866	165.92	-37.32	4.64
867	166.11	-37.06	4.64
868	166.36	-36.75	4.64
869	166.56	-36.38	4.64
870	166.76	-35.96	4.64
871	166.72	-35.47	4.64
872	167.01	-34.94	4.64
873	167.11	-34.35	4.64
874	167.43	-33.70	4.64
875	167.54	-33.01	4.64
876	167.79	-32.27	4.65
877	167.71	-31.50	4.65
878	167.98	-30.70	4.65
879	168.14	-29.89	4.65
880	168.4	-29.07	4.65
881	168.6	-28.26	4.65
882	168.73	-27.46	4.65
883	168.79	-26.67	4.65
884	168.97	-25.89	4.65
885	169.21	-25.14	4.65
886	169.4	-24.41	4.65
887	169.66	-23.70	4.65
888	169.66	-23.02	4.65
889	169.83	-22.36	4.65
890	169.94	-21.73	4.64
891	170.23	-21.12	4.64
892	170.37	-20.53	4.64
893	170.64	-19.96	4.64
894	170.65	-19.42	4.64
895	170.79	-18.89	4.64

896	172.55	-9.17	4.54
897	172.52	-9.06	4.54
898	172.83	-8.94	4.53
899	172.99	-8.83	4.53
900	173.31	-8.75	4.53
901	173.43	-8.64	4.53
902	173.54	-8.56	4.53
903	173.66	-8.48	4.53
904	173.87	-8.38	4.53
905	174.1	-8.30	4.53
906	174.33	-8.22	4.53
907	174.55	-8.16	4.53
908	174.55	-8.10	4.53
909	174.79	-8.02	4.53
910	174.92	-7.95	4.53
911	175.24	-7.87	4.54
912	175.38	-7.82	4.54
913	175.64	-7.76	4.54
914	175.61	-7.70	4.54
915	175.86	-7.65	4.54
916	176.02	-7.58	4.54
917	176.27	-7.55	4.54
918	176.49	-7.49	4.54
919	176.64	-7.46	4.54
920	176.71	-7.41	4.54
921	176.9	-7.36	4.54
922	177.16	-7.33	4.54
923	177.35	-7.28	4.54
924	177.64	-7.25	4.54
925	177.69	-7.22	4.54
926	177.83	-7.18	4.54
927	177.99	-7.14	4.54
928	178.25	-7.10	4.54
929	178.41	-7.07	4.54
930	178.71	-7.06	4.54
931	178.74	-7.02	4.54

896	172.48	-2.82	4.49
897	172.52	-2.82	4.49
898	172.68	-2.82	4.49
899	172.74	-2.83	4.49
900	173.04	-2.83	4.49
901	173.18	-2.83	4.49
902	173.49	-2.84	4.49
903	173.6	-2.84	4.49
904	173.66	-2.85	4.49
905	173.83	-2.85	4.49
906	174.04	-2.85	4.49
907	174.27	-2.86	4.50
908	174.46	-2.86	4.50
909	174.64	-2.87	4.50
910	174.62	-2.87	4.50
911	174.9	-2.88	4.50
912	175.01	-2.87	4.50
913	175.35	-2.88	4.50
914	175.45	-2.88	4.50
915	175.69	-2.89	4.50
916	175.65	-2.89	4.50
917	175.96	-2.90	4.50
918	176.1	-2.90	4.50
919	176.37	-2.91	4.50
920	176.57	-2.91	4.49
921	176.7	-2.92	4.49
922	176.76	-2.92	4.49
923	176.97	-2.92	4.49
924	177.21	-2.92	4.50
925	177.42	-2.93	4.50
926	177.67	-2.93	4.50
927	177.72	-2.94	4.50
928	177.89	-2.94	4.50
929	178.04	-2.94	4.50
930	178.34	-2.95	4.50
931	178.54	-2.95	4.50

896	161.64	-19.39	4.4215
897	161.7	-18.85	4.4215
898	162.01	-18.30	4.4215
899	162.22	-17.74	4.4215
900	162.56	-17.21	4.4215
901	162.813	-16.67	4.4215
902	163.088	-16.17	4.4215
903	163.363	-15.69	4.44
904	163.638	-15.23	4.44
905	163.913	-14.78	4.44
906	164.188	-14.36	4.44
907	164.463	-13.95	4.44
908	164.738	-13.56	4.44
909	165.013	-13.19	4.44
910	165.288	-12.86	4.44
911	165.563	-12.52	4.44
912	165.838	-12.23	4.44
913	166.113	-11.93	4.44
914	166.388	-11.66	4.44
915	166.663	-11.40	4.44
916	166.938	-11.14	4.44
917	167.213	-10.92	4.44
918	167.488	-10.69	4.44
919	167.763	-10.47	4.44
920	168.038	-10.27	4.44
921	168.313	-10.08	4.44
922	168.588	-9.90	4.44
923	168.863	-9.73	4.44
924	169.138	-9.56	4.44
925	169.413	-9.40	4.44
926	169.688	-9.25	4.44
927	169.963	-9.10	4.44
928	170.238	-8.95	4.44
929	170.513	-8.82	4.44
930	170.788	-8.70	4.44
931	171.063	-8.57	4.44

896	170.99	-18.39	4.64
897	171.2	-17.91	4.64
898	171.44	-17.45	4.64
899	171.57	-17.00	4.64
900	171.68	-16.57	4.64
901	171.75	-16.16	4.64
902	172.02	-15.77	4.64
903	172.15	-15.39	4.64
904	172.48	-15.03	4.64
905	172.52	-14.67	4.64
906	172.68	-14.34	4.64
907	172.74	-14.01	4.64
908	173.04	-13.70	4.64
909	173.18	-13.40	4.64
910	173.49	-13.11	4.64
911	173.6	-12.83	4.64
912	173.66	-12.57	4.64
913	173.83	-12.31	4.64
914	174.04	-12.06	4.64
915	174.27	-11.82	4.64
916	174.46	-11.59	4.64
917	174.64	-11.37	4.64
918	174.62	-11.15	4.64
919	174.9	-10.94	4.64
920	175.01	-10.74	4.64
921	175.35	-10.55	4.64
922	175.45	-10.37	4.64
923	175.69	-10.19	4.64
924	175.65	-10.01	4.64
925	175.96	-9.84	4.64
926	176.1	-9.68	4.64
927	176.37	-9.52	4.64
928	176.57	-9.37	4.64
929	176.7	-9.22	4.64
930	176.76	-9.07	4.64
931	176.97	-8.94	4.64

932	178.87	-6.99	4.54
933	179.05	-6.96	4.54
934	179.29	-6.93	4.54
935	179.53	-6.90	4.54
936	179.7	-6.88	4.54
937	179.8	-6.86	4.54
938	179.88	-6.83	4.54
939	180.16	-6.82	4.54
940	180.29	-6.80	4.54
941	180.63	-6.77	4.54
942	180.69	-6.75	4.54
943	180.83	-6.75	4.54
944	180.9	-6.74	4.54
945	181.2	-6.72	4.54
946	181.32	-6.70	4.54
947	181.62	-6.70	4.54
948	181.76	-6.69	4.54
949	181.82	-6.67	4.54
950	182	-6.66	4.54
951	182.22	-6.66	4.54
952	182.44	-6.62	4.54
953	182.64	-6.62	4.54
954	182.82	-6.61	4.54
955	182.82	-6.61	4.54
956	183.09	-6.59	4.53
957	183.22	-6.58	4.53
958	183.54	-6.56	4.53
959	183.68	-6.56	4.53
960	183.89	-6.56	4.53
961	183.87	-6.54	4.53
962	184.2	-6.54	4.53
963	184.32	-6.53	4.53
964	184.64	-6.53	4.53
965	184.82	-6.51	4.53
966	184.95	-6.51	4.53
967	185.02	-6.51	4.53

932	178.78	-2.95	4.50
933	178.78	-2.96	4.50
934	178.97	-2.96	4.50
935	179.15	-2.96	4.49
936	179.39	-2.97	4.49
937	179.63	-2.97	4.49
938	179.81	-2.98	4.49
939	179.89	-2.98	4.49
940	180.01	-2.99	4.49
941	180.3	-2.99	4.49
942	180.45	-2.99	4.50
943	180.75	-3.00	4.50
944	180.82	-3.01	4.50
945	180.97	-3.01	4.50
946	181.07	-3.02	4.50
947	181.38	-3.02	4.50
948	181.5	-3.02	4.50
949	181.82	-3.03	4.50
950	181.9	-3.03	4.50
951	181.99	-3.04	4.50
952	182.16	-3.04	4.50
953	182.39	-3.04	4.50
954	182.62	-3.04	4.50
955	182.83	-3.05	4.50
956	183	-3.05	4.50
957	182.99	-3.06	4.50
958	183.26	-3.06	4.50
959	183.42	-3.07	4.50
960	183.71	-3.07	4.50
961	183.82	-3.07	4.50
962	184.06	-3.08	4.50
963	184	-3.08	4.50
964	184.32	-3.09	4.50
965	184.42	-3.09	4.50
966	184.76	-3.10	4.50
967	184.9	-3.10	4.50

932	171.338	-8.45	4.44
933	171.613	-8.34	4.44
934	171.888	-8.23	4.44
935	172.163	-8.12	4.44
936	172.438	-8.03	4.4585
937	172.713	-7.94	4.4585
938	172.988	-7.84	4.4585
939	173.263	-7.77	4.4585
940	173.538	-7.68	4.4585
941	173.813	-7.60	4.4585
942	174.088	-7.51	4.4585
943	174.363	-7.44	4.44
944	174.638	-7.38	4.44
945	174.913	-7.31	4.44
946	175.188	-7.25	4.44
947	175.463	-7.18	4.44
948	175.738	-7.12	4.44
949	176.013	-7.07	4.44
950	176.288	-7.01	4.44
951	176.563	-6.96	4.44
952	176.838	-6.92	4.44
953	177.113	-6.86	4.44
954	177.388	-6.81	4.44
955	177.663	-6.77	4.44
956	177.938	-6.73	4.44
957	178.213	-6.70	4.44
958	178.488	-6.66	4.44
959	178.763	-6.62	4.44
960	179.038	-6.59	4.44
961	179.313	-6.55	4.44
962	179.588	-6.51	4.44
963	179.863	-6.49	4.44
964	180.138	-6.46	4.44
965	180.413	-6.44	4.44
966	180.688	-6.40	4.44
967	180.963	-6.38	4.44

932	177.21	-8.80	4.64
933	177.42	-8.67	4.64
934	177.67	-8.55	4.64
935	177.72	-8.42	4.64
936	177.89	-8.31	4.64
937	178.04	-8.19	4.64
938	178.34	-8.08	4.64
939	178.54	-7.97	4.64
940	178.78	-7.87	4.64
941	178.78	-7.76	4.64
942	178.97	-7.67	4.64
943	179.15	-7.57	4.64
944	179.39	-7.49	4.64
945	179.63	-7.39	4.64
946	179.81	-7.31	4.64
947	179.89	-7.23	4.64
948	180.01	-7.15	4.64
949	180.3	-7.07	4.64
950	180.45	-7.00	4.64
951	180.75	-6.93	4.64
952	180.82	-6.85	4.64
953	180.97	-6.79	4.64
954	181.07	-6.72	4.64
955	181.38	-6.65	4.64
956	181.5	-6.60	4.64
957	181.82	-6.54	4.64
958	181.9	-6.48	4.64
959	181.99	-6.42	4.64
960	182.16	-6.37	4.64
961	182.39	-6.32	4.64
962	182.62	-6.27	4.64
963	182.83	-6.22	4.64
964	183	-6.17	4.64
965	182.99	-6.12	4.64
966	183.26	-6.08	4.64
967	183.42	-6.03	4.64

968	185.25	-6.50	4.54
969	185.49	-6.50	4.53
970	185.71	-6.48	4.53
971	185.94	-6.48	4.53
972	185.98	-6.48	4.53
973	186.15	-6.46	4.53
974	186.31	-6.46	4.54
975	186.62	-6.46	4.54
976	186.8	-6.46	4.54
977	187.03	-6.46	4.54
978	187.02	-6.46	4.54
979	187.25	-6.46	4.54
980	187.41	-6.45	4.54
981	187.67	-6.46	4.54
982	187.88	-6.45	4.54
983	188.07	-6.46	4.54
984	188.1	-6.46	4.54
985	188.27	-6.46	4.54
986	188.51	-6.46	4.54
987	188.69	-6.46	4.54
988	188.98	-6.46	4.54
989	189.04	-6.46	4.54
990	189.17	-6.46	4.54
991	189.3	-6.46	4.54
992	189.58	-6.46	4.54
993	189.73	-6.46	4.54
994	190.03	-6.46	4.54
995	190.08	-6.46	4.54
996	190.17	-6.48	4.54
997	190.37	-6.46	4.54
998	190.57	-6.48	4.54
999	190.8	-6.48	4.54
1000	190.99	-6.48	4.54
1001	191.12	-6.50	4.54
1002	191.17	-6.50	4.54
1003	191.43	-6.50	4.54

968	185.05	-3.11	4.50
969	185.12	-3.10	4.50
970	185.35	-3.11	4.50
971	185.56	-3.11	4.49
972	185.76	-3.11	4.49
973	186.02	-3.11	4.49
974	186.04	-3.12	4.49
975	186.22	-3.12	4.50
976	186.35	-3.13	4.50
977	186.66	-3.13	4.50
978	186.8	-3.13	4.50
979	187.07	-3.14	4.50
980	187.05	-3.14	4.50
981	187.28	-3.15	4.50
982	187.45	-3.15	4.50
983	187.69	-3.16	4.50
984	187.91	-3.16	4.50
985	188.07	-3.17	4.50
986	188.13	-3.17	4.50
987	188.29	-3.18	4.50
988	188.53	-3.18	4.50
989	188.68	-3.18	4.50
990	188.98	-3.18	4.50
991	189.06	-3.19	4.50
992	189.18	-3.20	4.50
993	189.26	-3.20	4.51
994	189.59	-3.21	4.51
995	189.7	-3.21	4.51
996	190.02	-3.22	4.51
997	190.09	-3.22	4.51
998	190.19	-3.23	4.51
999	190.34	-3.24	4.51
1000	190.56	-3.24	4.51
1001	190.79	-3.25	4.51
1002	190.99	-3.25	4.51
1003	191.13	-3.26	4.51

968	181.238	-6.35	4.44
969	181.513	-6.33	4.44
970	181.788	-6.31	4.44
971	182.063	-6.29	4.44
972	182.338	-6.25	4.44
973	182.613	-6.23	4.44
974	182.888	-6.22	4.44
975	183.163	-6.22	4.44
976	183.438	-6.20	4.44
977	183.713	-6.18	4.44
978	183.988	-6.18	4.44
979	184.263	-6.16	4.44
980	184.538	-6.16	4.44
981	184.813	-6.14	4.44
982	185.088	-6.14	4.44
983	185.363	-6.12	4.44
984	185.638	-6.12	4.44
985	185.913	-6.11	4.44
986	186.188	-6.11	4.44
987	186.463	-6.09	4.44
988	186.738	-6.09	4.44
989	187.013	-6.07	4.44
990	187.288	-6.05	4.44
991	187.563	-6.05	4.44
992	187.838	-6.03	4.44
993	188.113	-6.03	4.44
994	188.388	-6.01	4.44
995	188.663	-6.01	4.44
996	188.938	-5.99	4.44
997	189.213	-5.99	4.44
998	189.488	-5.98	4.44
999	189.763	-5.98	4.44
1000	190.038	-5.98	4.44
1001	190.313	-5.96	4.44
1002	190.588	-5.96	4.44
1003	190.863	-5.96	4.44

968	183.71	-5.99	4.64
969	183.82	-5.95	4.64
970	184.06	-5.91	4.64
971	184	-5.86	4.64
972	184.32	-5.83	4.64
973	184.42	-5.79	4.64
974	184.76	-5.75	4.64
975	184.9	-5.72	4.64
976	185.05	-5.68	4.64
977	185.12	-5.65	4.64
978	185.35	-5.62	4.64
979	185.56	-5.58	4.64
980	185.76	-5.56	4.64
981	186.02	-5.53	4.64
982	186.04	-5.50	4.64
983	186.22	-5.47	4.64
984	186.35	-5.45	4.64
985	186.66	-5.42	4.64
986	186.8	-5.40	4.64
987	187.07	-5.38	4.64
988	187.05	-5.35	4.64
989	187.28	-5.34	4.64
990	187.45	-5.31	4.64
991	187.69	-5.30	4.64
992	187.91	-5.28	4.64
993	188.07	-5.27	4.64
994	188.13	-5.25	4.64
995	188.29	-5.23	4.64
996	188.53	-5.21	4.64
997	188.68	-5.20	4.64
998	188.98	-5.19	4.64
999	189.06	-5.17	4.64
1000	189.18	-5.17	4.64
1001	189.26	-5.17	4.64
1002	189.59	-5.17	4.64
1003	189.7	-5.17	4.64

1004	191.58	-6.51	4.54
1005	191.88	-6.51	4.54
1006	191.97	-6.53	4.54
1007	192.12	-6.53	4.54
1008	192.14	-6.54	4.53
1009	192.43	-6.54	4.53
1010	192.54	-6.54	4.54
1011	192.86	-6.54	4.53
1012	192.96	-6.56	4.53
1013	193.04	-6.58	4.53
1014	193.19	-6.56	4.53
1015	193.39	-6.58	4.53
1016	193.61	-6.58	4.53
1017	193.81	-6.58	4.53
1018	194.02	-6.58	4.53
1019	193.98	-6.59	4.54
1020	194.23	-6.59	4.54
1021	194.35	-6.59	4.54
1022	194.66	-6.59	4.54
1023	194.78	-6.61	4.54
1024	195	-6.61	4.54
1025	194.96	-6.61	4.53
1026	195.22	-6.61	4.54
1027	195.35	-6.61	4.54
1028	195.65	-6.61	4.54
1029	195.83	-6.61	4.54
1030	195.95	-6.62	4.54
1031	196.02	-6.61	4.53
1032	196.23	-6.62	4.54
1033	196.45	-6.62	4.53
1034	196.63	-6.62	4.53
1035	196.9	-6.61	4.54
1036	196.92	-6.62	4.54
1037	197.07	-6.62	4.54
1038	197.19	-6.62	4.53
1039	197.49	-6.62	4.53

1004	191.13	-3.27	4.51
1005	191.41	-3.27	4.51
1006	191.54	-3.28	4.51
1007	191.85	-3.28	4.51
1008	192	-3.29	4.51
1009	192.2	-3.29	4.51
1010	192.15	-3.30	4.51
1011	192.47	-3.31	4.50
1012	192.58	-3.31	4.50
1013	192.89	-3.32	4.50
1014	193.02	-3.32	4.50
1015	193.17	-3.33	4.50
1016	193.22	-3.34	4.50
1017	193.45	-3.35	4.50
1018	193.64	-3.35	4.50
1019	193.89	-3.36	4.50
1020	194.1	-3.36	4.50
1021	194.12	-3.37	4.50
1022	194.31	-3.37	4.51
1023	194.46	-3.38	4.51
1024	194.72	-3.38	4.51
1025	194.87	-3.39	4.51
1026	195.12	-3.39	4.51
1027	195.1	-3.40	4.51
1028	195.32	-3.41	4.51
1029	195.47	-3.41	4.52
1030	195.72	-3.42	4.52
1031	195.89	-3.42	4.52
1032	196.08	-3.43	4.52
1033	196.12	-3.43	4.52
1034	196.28	-3.44	4.52
1035	196.51	-3.44	4.52
1036	196.69	-3.45	4.52
1037	196.95	-3.46	4.52
1038	197.03	-3.46	4.52
1039	197.13	-3.47	4.52

1004	191.138	-5.96	4.44
1005	191.413	-5.96	4.44
1006	191.688	-5.96	4.44
1007	191.963	-5.96	4.44
1008	192.238	-5.96	4.44
1009	192.513	-5.94	4.44
1010	192.788	-5.94	4.44
1011	193.063	-5.94	4.44
1012	193.338	-5.94	4.44
1013	193.613	-5.94	4.44
1014	193.888	-5.94	4.44
1015	194.163	-5.93	4.44
1016	194.438	-5.93	4.44
1017	194.713	-5.93	4.44
1018	194.988	-5.93	4.44
1019	195.263	-5.92	4.44
1020	195.538	-5.92	4.44
1021	195.813	-5.92	4.44
1022	196.088	-5.92	4.44
1023	196.363	-5.92	4.44
1024	196.638	-5.91	4.44
1025	196.913	-5.91	4.44
1026	197.188	-5.91	4.44
1027	197.463	-5.91	4.44
1028	197.738	-5.91	4.44
1029	198.013	-5.90	4.44
1030	198.288	-5.90	4.44
1031	198.563	-5.90	4.44
1032	198.838	-5.90	4.44
1033	199.113	-5.90	4.44
1034	199.388	-5.89	4.44
1035	199.663	-5.89	4.44
1036	199.938	-5.89	4.44
1037	200.213	-5.89	4.44
1038	200.488	-5.88	4.44
1039	200.763	-5.88	4.44

1004	190.02	-5.17	4.64
1005	190.09	-5.16	4.64
1006	190.19	-5.16	4.64
1007	190.34	-5.16	4.64
1008	190.56	-5.16	4.64
1009	190.79	-5.16	4.64
1010	190.99	-5.16	4.64
1011	191.13	-5.15	4.64
1012	191.13	-5.15	4.64
1013	191.41	-5.15	4.64
1014	191.54	-5.15	4.64
1015	191.85	-5.14	4.64
1016	192	-5.14	4.64
1017	192.2	-5.13	4.64
1018	192.15	-5.13	4.64
1019	192.47	-5.13	4.64
1020	192.58	-5.12	4.64
1021	192.89	-5.12	4.64
1022	193.02	-5.11	4.64
1023	193.17	-5.11	4.64
1024	193.22	-5.11	4.64
1025	193.45	-5.11	4.64
1026	193.64	-5.11	4.64
1027	193.89	-5.11	4.64
1028	194.1	-5.10	4.64
1029	194.12	-5.10	4.64
1030	194.31	-5.10	4.64
1031	194.46	-5.10	4.64
1032	194.72	-5.09	4.64
1033	194.87	-5.09	4.64
1034	195.12	-5.09	4.64
1035	195.1	-5.08	4.64
1036	195.32	-5.08	4.64
1037	195.47	-5.08	4.64
1038	195.72	-5.08	4.64
1039	195.89	-5.07	4.64

1040	197.63	-6.62	4.53
1041	197.9	-6.62	4.53
1042	197.91	-6.64	4.53
1043	198.08	-6.64	4.53
1044	198.26	-6.64	4.53
1045	198.51	-6.64	4.53
1046	198.74	-6.64	4.53
1047	198.9	-6.66	4.53
1048	198.96	-6.66	4.53
1049	199.11	-6.66	4.53
1050	199.35	-6.66	4.53
1051	199.5	-6.66	4.53
1052	199.8	-6.66	4.53
1053	199.89	-6.67	4.53
1054	200.01	-6.67	4.53
1055	200.11	-6.67	4.53
1056	200.44	-6.67	4.53
1057	200.56	-6.67	4.53
1058	200.88	-6.67	4.53
1059	200.96	-6.67	4.53
1060	201.05	-6.69	4.53
1061	201.24	-6.69	4.53
1062	201.44	-6.69	4.53
1063	201.7	-6.69	4.53

1040	197.25	-3.48	4.52
1041	197.54	-3.48	4.52
1042	197.63	-3.49	4.52
1043	197.96	-3.49	4.52
1044	197.97	-3.50	4.52
1045	198.1	-3.51	4.52
1046	198.23	-3.52	4.52
1047	198.49	-3.52	4.52
1048	198.68	-3.53	4.52
1049	198.89	-3.54	4.52
1050	199.03	-3.54	4.52
1051	199.04	-3.56	4.52
1052	199.28	-3.56	4.52
1053	199.42	-3.58	4.52
1054	199.7	-3.58	4.51
1055	199.84	-3.59	4.51
1056	200.02	-3.60	4.51
1057	200.01	-3.62	4.51
1058	200.3	-3.62	4.51
1059	200.38	-3.64	4.51
1060	200.72	-3.65	4.51
1061	200.82	-3.66	4.51
1062	201.01	-3.67	4.50
1063	201.02	-3.69	4.50
1064	201.3	-3.70	4.50
1065	201.45	-3.71	4.50
1066	201.7	-3.73	4.50
1067	201.9	-3.74	4.50
1068	201.96	-3.76	4.50

1040	201.038	-5.88	4.44
1041	201.313	-5.88	4.44
1042	201.588	-5.88	4.44
1043	201.863	-5.87	4.44
1044	202.138	-5.87	4.44

1040	196.08	-5.07	4.64
1041	196.12	-5.07	4.64
1042	196.28	-5.07	4.64
1043	196.51	-5.06	4.64
1044	196.69	-5.06	4.64
1045	196.95	-5.06	4.64
1046	197.03	-5.06	4.64
1047	197.13	-5.05	4.64
1048	197.25	-5.05	4.64
1049	197.54	-5.05	4.64
1050	197.63	-5.04	4.64
1051	197.96	-5.11	4.64
1052	197.97	-5.05	4.64
1053	198.1	-5.05	4.64
1054	198.23	-5.05	4.64
1055	198.49	-5.04	4.64
1056	198.68	-5.04	4.64
1057	198.89	-5.04	4.64
1058	199.03	-5.04	4.64
1059	199.04	-5.04	4.64
1060	199.28	-5.03	4.64
1061	199.42	-5.03	4.64
1062	199.7	-5.03	4.64
1063	199.84	-5.03	4.64
1064	200.02	-5.03	4.64
1065	200.01	-5.02	4.64
1066	200.3	-5.02	4.64
1067	200.38	-5.02	4.64
1068	200.72	-5.02	4.64
1069	200.82	-5.02	4.64
1070	201.01	-5.01	4.64
1071	201.02	-5.01	4.64
1072	201.3	-5.01	4.64
1073	201.45	-5.01	4.64
1074	201.7	-5.01	4.64
1075	201.9	-5.00	4.64

1076	201.96	-5.00	4.64
1077	201.88	-5.00	4.64
