# Supplementary material for: Dermal formulation based on carbopol and Gum Arabic improves skin retention of indomethacin
Source: PLoS One. 2025 Jun 10;20(6):e0326051. doi: 10.1371/journal.pone.0326051 (PMC12151425; doi:10.1371/journal.pone.0326051)
Supplement: S3 Table — Raw data in Figs 1 and 3–6. (PDF) [file pone.0326051.s005.pdf]

Figure 1

Particle size	Mean (μm)	S.E.(μm)
Figure 1A	15.6	0.35
Figure 1B	0.0089	0.0018
Figure 1C	0.1097	0.0038

Figure 1D	Data is presented in a separate file

Figure 2

Data is presented in a other sheet

Figure 3

Figure 3A

Non-uniformity in IMC gel	Mean (mM)	S.E.(μM)
IMC-MP@GCgel	0.43	0.045
IMC-NP@GCgel	0.16	0.031

Figure 3B

IMC solubility in gel	Mean (mM)	S.E.(μM)
IMC-MP@GCgel	0.22	0.03
IMC-NP@GCgel	0.5	0.03

Figure 3C

Viscosity	Mean (Pa·s)	S.E.(Pa·s)
Vehicle	2.41	0.26
IMC-MP@GCgel	2.27	0.47
IMC-NP@GCgel	2.17	0.3

Figure 4

Figure 4A **Mean**

IMC con. (μmol/cm2)	IMC-MP@GCgel	IMC-NP@GCgel
1 h	0.423	0.785
2 h	0.773	1.461
3 h	1.195	2.282
6 h	2.245	4.153
24 h	2.801	4.322

Figure 4A **S.E.**

IMC con.	IMC-MP@GCgel	IMC-NP@GCgel
1 h	0.07	0.08
2 h	0.08	0.06
3 h	0.14	0.16
6 h	0.24	0.22
24 h	0.16	0.12

Figure 4B

AUC0-24h release	Mean (μmol·h/cm^2)	S.E. (μmol·h/cm2)
IMC-MP@GCgel	60.36	4.34
IMC-NP@GCgel	99.9	3.62

Figure 4C (×10^8 particles/mL)

Nanoparticle number	Mean (mM)	S.E.(μM)
IMC-MP@GCgel	N.D.	N.D.
IMC-NP@GCgel	7.75	0.7

Figure 4D

IMC particles	Mean(nm)	S.E(nm)
IMC-NP@Gcgel	154.7	10.1

Figure 5

Figure 5A **Mean**

IMC con. (μmol/cm2)	IMC-MP@GCgel	IMC-NP@GCgel
1 h	0.111	0.091
2 h	0.161	0.161
3 h	0.211	0.312
6 h	0.292	1.046
24 h	1.610	4.245

Figure 5A **S.E.**

IMC con.	IMC-MP@GCgel	IMC-NP@GCgel
1 h	0.051	0.061
2 h	0.061	0.048
3 h	0.082	0.087
6 h	0.091	0.111
24 h	0.211	0.201

Figure 5B

AUC0-24h penetration	Mean (μmol·h/cm^2	S.E. (μmol·h/cm^2)
IMC-MP@GCgel	16.7	3.38
IMC-NP@GCgel	49.2	3.26

Figure 6

Figure6A **Mean**

IMC con. (nmol/mL)	IMC-MP@GCgel	IMC-NP@GCgel
1 h	0.619	0.483
2 h	0.960	0.827
3 h	1.032	1.026
6 h	0.984	1.102
24 h	0.893	0.842

Figure 6A **S.E.**

IMC con. (nmol/mL)	IMC-MP@GCgel	IMC-NP@GCgel
1 h	0.048	0.033
2 h	0.024	0.075
3 h	0.136	0.133
6 h	0.042	0.103
24 h	0.063	0.060

Figure 6B

IMC con. (μmol/mg p	Mean	S.E.
IMC-MP@GCgel	0.26	0.13
IMC-NP@GCgel	6.82	0.9
