# Supplementary material for: Dermal formulation based on carbopol and Gum Arabic improves skin retention of indomethacin
Source: PLoS One. 2025 Jun 10;20(6):e0326051. doi: 10.1371/journal.pone.0326051 (PMC12151425; doi:10.1371/journal.pone.0326051)
Supplement: Attachment — Submitted filename: Review PONE-D-25-07074.docx [file pone.0326051.s006.docx]

Review Comments to the Author

**Draft Manuscript**: Dermal formulation based on carbopol and gum arabic improves skin retention of indomethacin

The study could contribute to pharmaceutical sciences and dermatological formulations by providing an improved method for delivering indomethacin through the skin. In this paper, authors evaluated penetration, retention, drug delivery and systemic absorption of indomethacin, using two different gel formulations, based on microparticles or nanoparticles.

The claim is well supported by the current literature.

The details of the methodology are sufficient to allow the experiments to be reproduced.

Experiments are appropriate and well-designed, although data analysis is not always rigorous, leading to conclusions that are not fully substantiated. Although in its current form the paper is unsuitable for publication, it shows good potential and authors are encouraged to resubmit a revised version.

The manuscript is overall well organized and written in a clear scientific English. A certificate of editing is available.

**Comments and Requests for revision:**

Line 80: incomplete sentence. Please, revise for clarity.

Lines 318-319: The claim that particles in IMP-NC are more evenly distributed than in IMP-MC is not sufficiently supported by Image 1D alone. Further justification or quantitative analysis is needed.

Line 335: a scale bar should be added to figure 1D.

Line 372: Instead of a multiple comparison, compare each parameter of the two formulations individually using a Student’s t-test or a non-parametric test if normality is not satisfied.

Line 376: The text (lines 365-366) states that no significant differences were observed in the reported parameters between the two formulations, but the statistical test you used is not specified. Please, clarify.

Line 385: ANOVA One-Way with Tukey-Kramer post hoc test is not suitable in this context, since measurements overtime are not independent (aliquots at different time points come from the same sample, thus they are dependent). A repeated-measures ANOVA should be used if normality is satisfied.

Line 391: The caption should specify that this is the in vitro approach.

Line 393: As above, ANOVA One-Way with Tukey-Kramer post hoc test is inappropriate, since also these measurements are not independent each other. Use repeated-measures ANOVA if normality is met.

Lines 395-397: You would put this sentence in the main text and discuss its implication, as it suggests that that the IMC (but not the carrier) can pass through the skin, at least in vitro.

Line 401: The caption is inconsistent with the graph (Fig. 6A), which shows a time scale rather than plasma IMC levels 6 hours after treatment. Please correct this inconsistency.
