# Supplementary material for: Dermal formulation based on carbopol and Gum Arabic improves skin retention of indomethacin
Source: PLoS One. 2025 Jun 10;20(6):e0326051. doi: 10.1371/journal.pone.0326051 (PMC12151425; doi:10.1371/journal.pone.0326051)
Supplement: Attachment — Submitted filename: 250502Response_to_Reviewers.docx [file pone.0326051.s009.docx]

**Response to Reviewer Comments**

We carefully revised our manuscript according to the suggestions of the reviewer 2, and details are as follows.

**<Q and A for Reviewer 2>**

**Q1. The authors have addressed all the comments raised in the first round of review. Although the request to adopt a different and more appropriate method of data analysis has been accepted and the changes have been implemented in the main text and/or figure caption, the statistical significance remains exactly the same as in the previous, incorrect analysis. The authors are kindly requested to check for any typos or inconsistencies in the revised version of the manuscript, or to demonstrate the validity of the results obtained from the newly performed analysis.**

A1. The reviewer’s comment is correct. We have appropriately corrected the symbols in Figures 4A and 5A. In addition, we have revised the corresponding descriptions in the manuscript, and carefully checked that the content is accurately reflected. Thank you very much for pointing this out (line 322, 424, 432, Figure 4A and 5A).

Thank you for great comments.
